# DNA hypo-methylation facilitates anti-inflammatory responses in severe ulcerative colitis

**DOI:** 10.1371/journal.pone.0248905

**Published:** 2021-04-01

**Authors:** Hagar Taman, Christopher G. Fenton, Endre Anderssen, Jon Florholmen, Ruth H. Paulssen

**Affiliations:** 1 Clinical Bioinformatics Research Group, Department of Clinical Medicine, UiT- The Arctic University of Norway, Tromsø, Norway; 2 Genomics Support Centre Tromsø (GSCT), Department of Clinical Medicine, UiT- The Arctic University of Norway, Tromsø, Norway; 3 Gastroenterology and Nutrition Research Group, Department of Clinical Medicine, UiT- The Arctic University of Norway, Tromsø, Norway; Toho University Graduate School of Medicine, JAPAN

## Abstract

Severe ulcerative colitis (UC) is a potentially life-threatening disease with a potential colorectal cancer (CRC) risk. The aim of this study was to explore the relationship between transcriptomic and genome-wide DNA methylation profiles in a well-stratified, treatment-naïve severe UC patient population in order to define specific epigenetic changes that could be responsible for the grade of disease severity. Mucosal biopsies from treatment-naïve severe UC patients (n = 8), treatment-naïve mild UC (n = 8), and healthy controls (n = 8) underwent both whole transcriptome RNA-Seq and genome-wide DNA bisulfite- sequencing, and principal component analysis (PCA), cell deconvolutions and diverse statistical methods were applied to obtain a dataset of significantly differentially expressed genes (DEGs) with correlation to DNA methylation for severe UC. DNA hypo-methylation correlated with approximately 80% of all DEGs in severe UC when compared to mild UC. Enriched pathways of annotated hypo-methylated genes revealed neutrophil degranulation, and immuno-regulatory interactions of the lymphoid system. Specifically, hypo-methylated anti-inflammatory genes found for severe UC were IL10, SIGLEC5, CD86, CLMP and members of inflammasomes NLRP3 and NLRC4. Hypo-methylation of anti-inflammatory genes during severe UC implies an interplay between the epithelium and lamina propria in order to mitigate inflammation in the gut. The specifically DNA hypo-methylated genes found for severe UC can potentially be useful biomarkers for determining disease severity and in the development of new targeted treatment strategies for severe UC patients.

## Introduction

Ulcerative colitis (UC) is an inflammatory disorder that affects the mucosa and submucosa of the colon and rectum and is a chronic disease with a relapsing course [1]. Disease severity is wide ranging with most UC patients manifesting a mild to moderate disease activity [2, 3]. However, between 15–30% of UC patients will experience at least one incident of acute severe colitis during the disease course, requiring hospitalization for immediate medical treatment [2, 4, 5]. Patients whose inflammation is more severe and more extensive are more likely to develop CRC [6]. In cases where medical therapy fails, colectomy is considered [2–5, 7]. The underlying causes of UC are still not completely understood. It has been suggested that UC is the result of a dysregulated immune response to environmental factors and commensal pathogens in a genetically predisposed host [8, 9]. Therefore, epigenetic mechanisms, such as DNA methylation have been implied to play a key role in disease development of UC [10–13]. Methylation of cytosine groups in DNA molecules can change the structure and interactions of a DNA sequence without changing the sequence [14]. In mammals, methylation primarily occurs in CpG dinucleotides and when occurring in CpG rich areas of promoters is linked to lasting stable repression of gene expression [15].

Epigenetic modifications, such as DNA hyper-methylation are believed to have a role in the immune dysfunction associated with IBD [12, 13]. However, less attention has been devoted to the role of DNA hypo-methylation for UC which represents one of the major DNA methylation states that refers to a relative decrease from an ordinary methylation level. UC by itself might induce hypo-methylation of DNA and a decrease in DNA methylation can have an impact on the predisposition to pathological states and UC development. Global DNA hypo-methylation has been suggested to contribute to neoplastic transformation which suggest that DNA hypo- methylation plays a previously unappreciated role in intestinal adenoma initiation [16].

Recently, whole transcriptomic and genome-wide DNA methylation profiles for treatment-naïve UC have been established for mild and moderate disease [17, 18]. This study focuses on the role of DNA hypo-methylation in a severe UC phenotype in comparison to a mild UC phenotype with the aim to identify DNA hypo-methylation patterns that might correlate with disease severity. This attempt makes it possible to identify biomarker groups that can help determine new potential personalized treatment targets for patients with severe UC and might improve the clinical outcome for this patient group.

## Materials and methods

### Patient material

Twenty-four mucosal biopsies were collected with a standardized sampling method from three patient groups, newly diagnosed treatment-naïve UC patients with severe disease activity (n = 8), newly diagnosed treatment-naïve UC patients with mild disease activity (n = 8), and normal control patients (n = 8). The biopsies were taken from the recto-sigmoid part of the colon. Subjects which underwent cancer screening, and showed normal colonoscopy and normal colonic histological examination, served as controls. Diagnosis of UC disease activity was based on established clinical, endoscopic and histological criteria as defined by the ECCO guidelines [19]. The inflammation grade was evaluated during colonoscopy using the UC disease activity index (UCDAI) [20]. Control biopsies showed normal colonoscopy, normal colon histology and immunohistochemistry, with a clinical and an endoscopic score of 0. TNF-α mRNA expression was detected by quantitative real-time polymerase chain reaction (qPCR) [21]. All patient characteristics are depicted in Table 1. The samples were taken from an established Biobank approved by the Norwegian Board of Health. The study was approved by the Regional Ethics Committee of North Norway and the Norwegian Social Science Data Services (REK Nord 2012/1349).

**Table 1:** Patients characteristics.

Characteristics	Control (n = 8)	UC mild (n = 8)	UC severe (n = 8)
Male/Female	5/3	6/2	6/2
Age mean ± SD	54.1 ± 22.3	39.6 ± 15.2	45.1 ± 24.4
TNF-α Level ± SD	4246 ± 1973	8400 ± 3280	31350 ± 26916
Endo Score mean ± SD	0	1.75 ± 0.46	2.38 ± 0.52
Clinical Score ± SD	0	7.75± 1.48	9.75 ± 2.12

SD, standard deviation; TNF, tumour necrosis factor

### DNA and RNA isolation

Genomic DNA and total RNA were isolated with the Allprep DNA/RNA Mini Kit from Qiagen (Cat no: 80204) and the QIAcube instrument (QIAGEN, Hilden, Germany), according to the manufacturer’s instructions. The quantity and quality of both DNA and RNA were assessed with Qubit 3 and Nanodrop One (Thermo Fisher Scientific, Wilmington, Delaware, USA), respectively. RNA integrity was evaluated with the Experion Automated Electrophoresis System (Bio-Rad, Hercules, CA, USA) and the RNA StdSens Analysis Kit (Bio-Rad, cat no: 700–7103), according to the manufacturer’s protocol. All RNA samples used for this analysis had a RIN value between 8.0–10.0. Both DNA and RNA were kept at -70°C until further use.

### Quantitative polymerase chain reaction (qPCR)

Quantitative polymerase chain reaction (qPCR) were used to measure TNF-α mRNA levels in all biopsies. RNA quantity was assessed with NanoVue Plus (GE Healthcare, UK). cDNA synthesis was performed with QuantiTect Reverse Transcription Kit (Qiagen, cat no: 205314), and the QuantiNova Probe PCR Kit (Qiagen, cat no: 208256). CFX Connect Real Time PCR Detection System (Bio-Rad, Hercules, CA, USA) was used for detection. The results were measured in copies/μg. Tissue samples with values <7000 copies/μg are considered non-inflamed, while tissue samples with >7000 copies/μg are considered inflamed [21].

### Library preparation and next generation sequencing

DNA libraries were prepared with the SeqCap Epi CpGiant Enrichment Kit (Roche, Switzerland). DNA was bisulfite converted using the EZ DNA Methylation-lightning Kit (Zymo Research, USA, cat no: D5030) prior to the hybridization step and according to the manufacturer’s instructions. The amount of input material was 1060 ng of genomic DNA per sample. DNA libraries quality were assessed using the Bioanalyzer 2100, and the Agilent DNA 1000 kit (cat no: 5067–1504, Agilent Technologies, Santa Clara, USA), according to the manufacturer’s instructions. DNA libraries generated fragments with an average size of 322 bp. DNA libraries were diluted to 2 nM prior to sequencing. Whole transcriptome libraries were prepared with the TruSeq Stranded Total RNA LT Sample Prep Kit from Illumina (cat no: RS-122-2203). The amount of input material was 1μg of total RNA. The Bioanalyzer 2100 and the Agilent DNA 1000 kit (cat no: 5067–1504, Agilent Technologies, Santa Clara, USA) were used to assess the quality of the RNA libraries. RNA libraries generated fragments with an average size of 301 bp, libraries were normalized to 10 nM and diluted to 4 nM prior to sequencing. Both DNA and RNA libraries were sequenced on the NextSeq 550 instrument, using a high output flow cell 150 cycles (cat no: FC-404-2002, Illumina, USA) and according to the manufacturer’s instruction. The libraries were sequenced using paired-end mode.

### Data analysis

Base calling, quality scoring and quality check were performed as a first step including quality check on the on-board computer of the NextSeq 550. The data analysis was carried out in the Bioconductor R framework (www.bioconductor.org). STAR-2.5.2b (https://github.com/alexdobin/STAR) was used to align raw Illumina reads to UCSC genome browser GRCH38p.11 (https://www.ncbi.nlm.nih.gov/grc/human/data). Htseq-count was used for generating the raw gene count matrix [22]. DESeq2 was used to Vst-normalize the gene count matrix [23], and compare severe UC vs mild UC in R (3.5.3) (https://doi.org/10.18129/B9.bioc.DESeq) [24]. Differentially expressed genes (DEGs) between severe UC vs mild UC transcripts were filtered with a read count > 30 and a corrected p < 0.05. P-values were corrected for multiple testing using the method of Benjamini and Hochberg [25].

Pathway enrichment was performed using ReactomePA bioconductor packages hypergeometric model (http://bioconductor.org/packages/release/bioc/html/ReactomePA.html). ReactomePA hypergeometric model assesses whether the number of selected genes associated with a reactome pathway is significantly larger than expected. P-values were corrected for multiple testing using the method of Benjamini and Hochberg [25]. Principal component analysis (PCA) of the transcriptome data was performed using the 1000 most variable genes [26]. Genes associated with the risk of IBD were downloaded from the genome-wide association studies (GWAS) catalogue, using the search term IBD (www.ebi.ac.uk/gwas) [27].

For DNA methylation analyses, the Bismark Bisulfite Mapper v0.16.0 (www.bioinformatics.bbsrc.ac.uk/projects/bismark/) was used to align reads to the same aforementioned genome build and calculate methylated and un-methylated DNA positional count matrices. Relative methylation is expressed as a number between 0–1 where 0 means 0% of C’s are methylated at that position and 1 means 100% or all C’s are methylated. The global methylation analysis mapped included more than 9 million cytosine sites genome- wide. In order to improve interpretation of the dataset, further analysis was restricted to genomic regions within the promoter regions of severe UC compared to mild UC DEGs. Significant differential methylation patterns from above DEGs were found using the globalTest function of the BiSeq Bioconductor package (https://www.bioconductor.org/packages/release/bioc/html/BiSeq.html). Only promoters with a global test p value less than 0.05 where kept. The promoter region was defined as 2000 bp upstream and 200 bp downstream of the transcription start site (TSS). Note that the same patients were used to generate both the methylation and the gene expression data. We could therefore correlate the average promoter relative methylation to the corresponding gene expression. Those promoter/gene pairs with correlations less than -0.6 were kept. A negative correlation occurs when methylation is high, and expression is low or vice versa. Global relative methylation patterns were analysed by principal component analysis (PCA).

Cell populations were estimated by absolute cell deconvolution using the RNA-Seq data. Samples raw counts per million were submitted to the absolute procedure of Monaco. This is a procedure specifically developed for deconvolution of human immune cell types from RNAseq data. Results were merged for T-cells, neutrophils, monocytes, and B-cell types to obtain four main types of immune cell populations [28]. The epithelial and stromal cell fractions were subsequently estimated based on the epithelial cell markers, epithelial cell adhesion molecule (EPCAM), cadherin 17 (CDH17), cadherin 1 (CDH1) and cadherin 18 (CDH18), and the stromal cell markers, endoglin (ENG), thy-1 cell surface antigen (THY1), actin alpha 2, smooth muscle (ACTA2) and collagen type II alpha 1 chain (COL2A1). Cell populations estimates were compared using ANOVA and Tukey’s range test [29].

## Results and discussion

In this study an integrative epigenome data set, combining genome-wide methylation data and whole-transcriptome data was established in order to gain insight into the molecular mechanisms of severe UC and to explore the epigenetic variation induced by severe inflammation of the colon. The chosen experimental design used in this study was to compare joined transcriptomic and DNA methylation data from each individual patient. This allows for rigorous analysis of the transcriptomic and DNA methylation status of UC patients irrespective of inter-individual differences in environmental or genetic background. In addition, the use of a thoroughly stratified patient group representing only treatment-naïve patients with severe UC for DNA methylation analysis offered a unique opportunity to investigate the DNA methylation state prior to prescription of any medication (Table 1). This is of importance, since UC medications such as immunosuppressive drugs have been shown to have short- and long-term side effects on immune response and can change DNA methylation status [30–33]. Genome-wide DNA methylation in treatment-naïve mild and moderate ulcerative colitis has been reported previously [18, 34]. In this study, we report specific DNA methylation patterns found for treatment-naïve severe UC.

**Fig 1:**
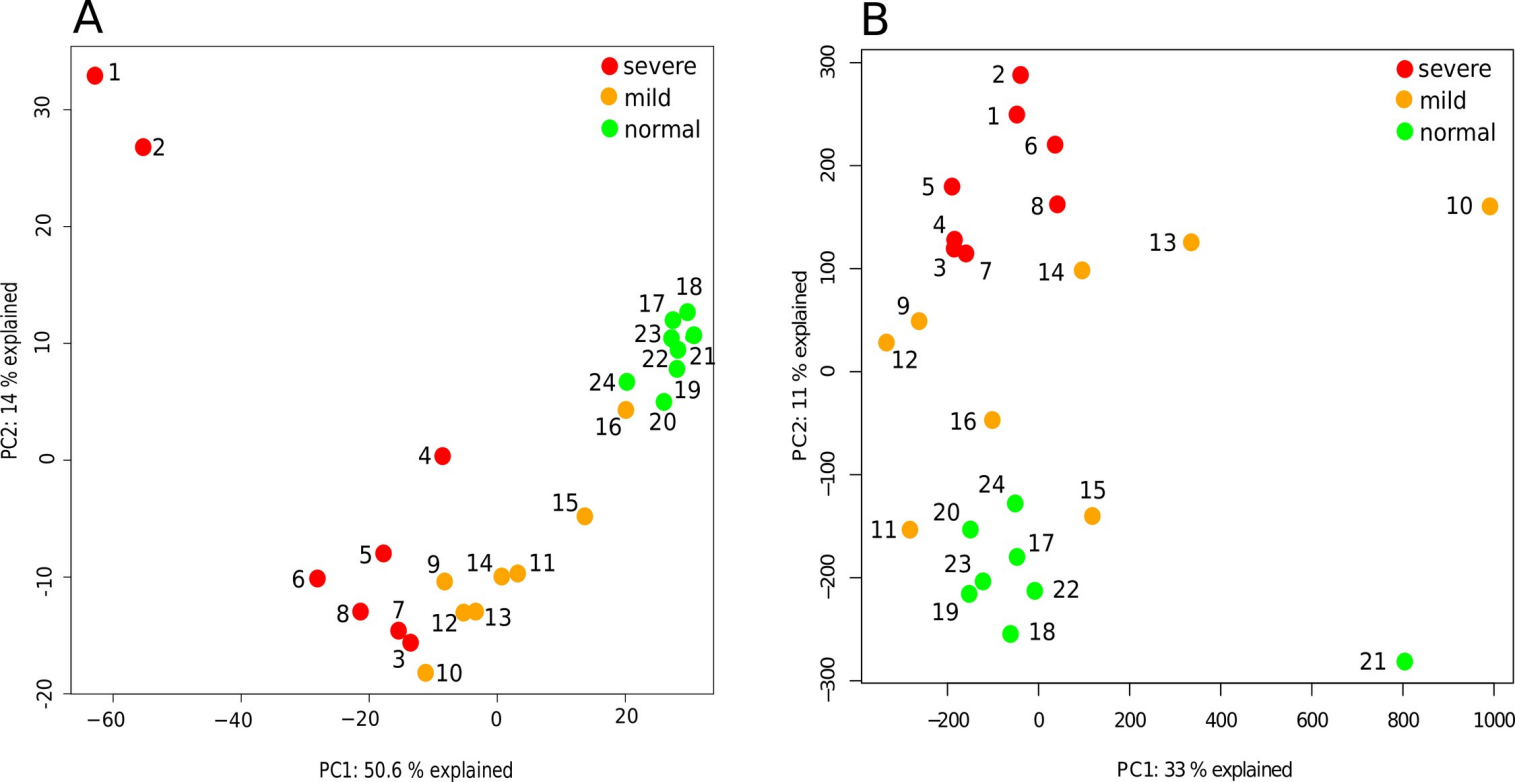
Principal component analysis (PCA). (A) PCA of gene expression data of the thousand most variable genes (transPCA). Unsupervised PCA analysis presenting the difference between severe UC (red, n = 8), mild UC (orange, n = 8) and control (green, n = 8). The first two components explain 51% and 13.5% of the variability in the gene expression data. (B) PCA depicting the global methylation (methPCA) of relative methylation counts (0–100%) for over 9 million cytosine positions including normal (green, n = 8), treatment-naïve mild UC (orange, n = 8) and severe UC (red, n = 8) patient tissue samples. The first two components explain 33% and 11% of the variability in the methylation data.

**Fig 2:**
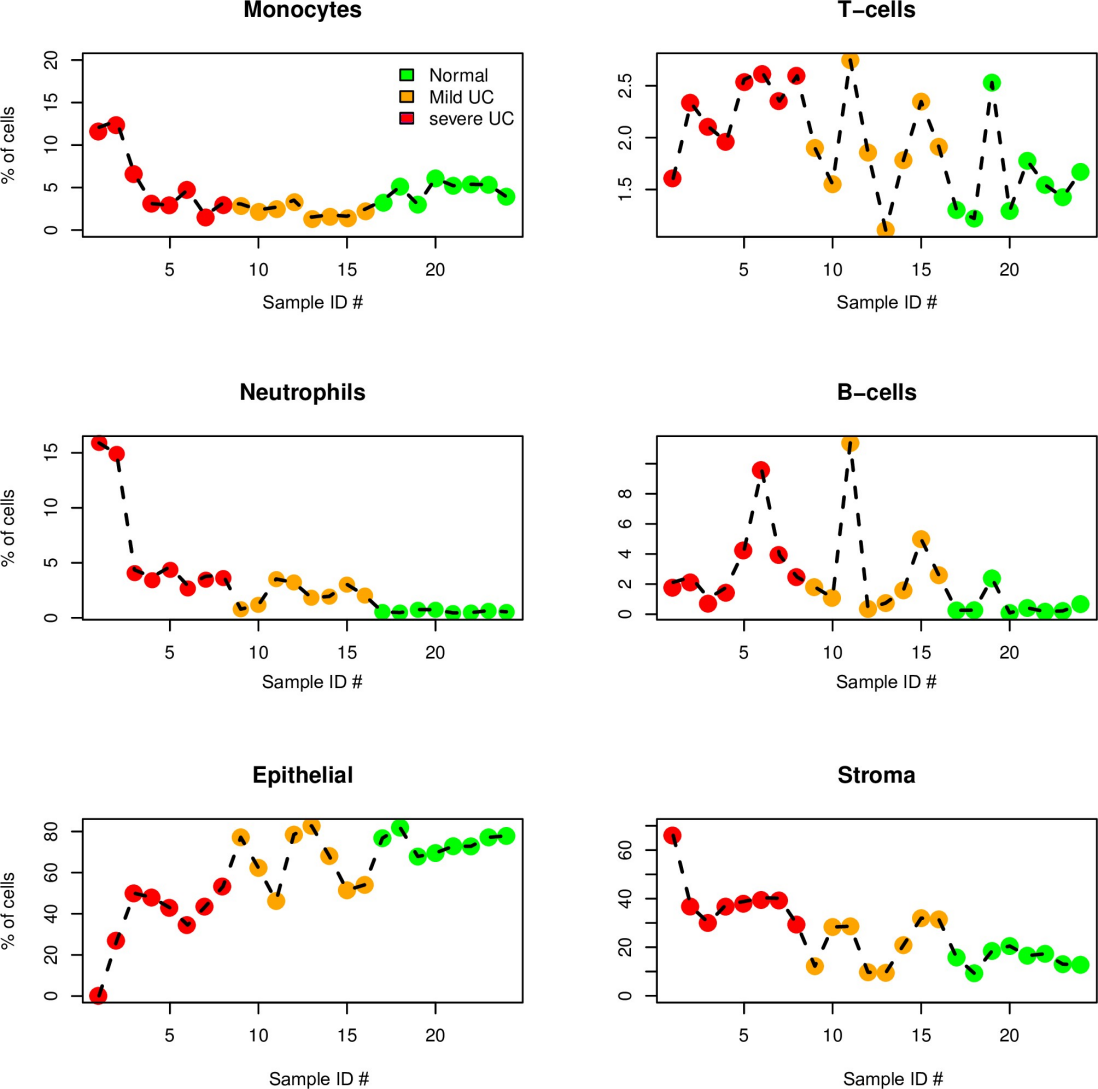
Cell fraction estimation between samples using cell deconvolution algorithm absolute deconvolution of human immune cell types. The fractions of different cell populations in severe and mild UC and control tissue samples were estimated from gene expression data, using absolute cell deconvolution as described in Materials and Methods. The deconvolutions were solved for the following cell types: epithelial cells, monocytes, T cells, neutrophils, B cells, and stroma cells. Each panel shows the estimated percentage of the indicated cell types (y-axis) across all 24 samples sorted according to sample ID numbers (y-axis). For ease of comparison, sample ID numbers are identical to those shown in PCA of methylation and gene expression data (Fig 1). Plot markers are colour coded according to sample group. The fractions of epithelial and stromal cells were estimated from the non-immune cell remainder and the expression levels of the stromal and epithelial marker genes. The epithelial markers (EPCAM, CDH1, CDH17 and CDH18) and stroma markers (ENG, THY1, ACTA2 and COL2A1) were used. Severe UC is indicated by red dots, mild UC is indicated by orange dots, and control is indicated by green dots. Statistical comparison of cell population estimates can be found in S4 Table.

Initial principal component analysis (PCA) revealed a clear separation of severe and mild UC patient phenotypes on both, the transcriptomic- and DNA methylation level (Fig 1). To prevent confusion, the different PCAs discussed in this study are designated transPCA representing transcriptomic data, and methPCA representing DNA methylation data. TransPCA of top thousand most variable differentially expressed genes resulted in a separation of severe and mild UC and control samples along the first principal component (PC1) with 50.6% explained variance, and 14% explained variance along the second principal component (PC2). A complete list of all DEGs is depicted in S1 Table. Differentially expressed IBD susceptibility genes (n = 47) are listed in S2 Table. Two of the UC samples (#1 and #2) in the transPCA separated from the severe patient sample group, probably indicating a different phenotype of severe UC (Fig 1A). Indeed, these extreme gene expressions may be related to high fractions of neutrophils and monocytes or loss of epithelial cells in these samples (Fig 2 and S4 Table).

**Fig 3:**
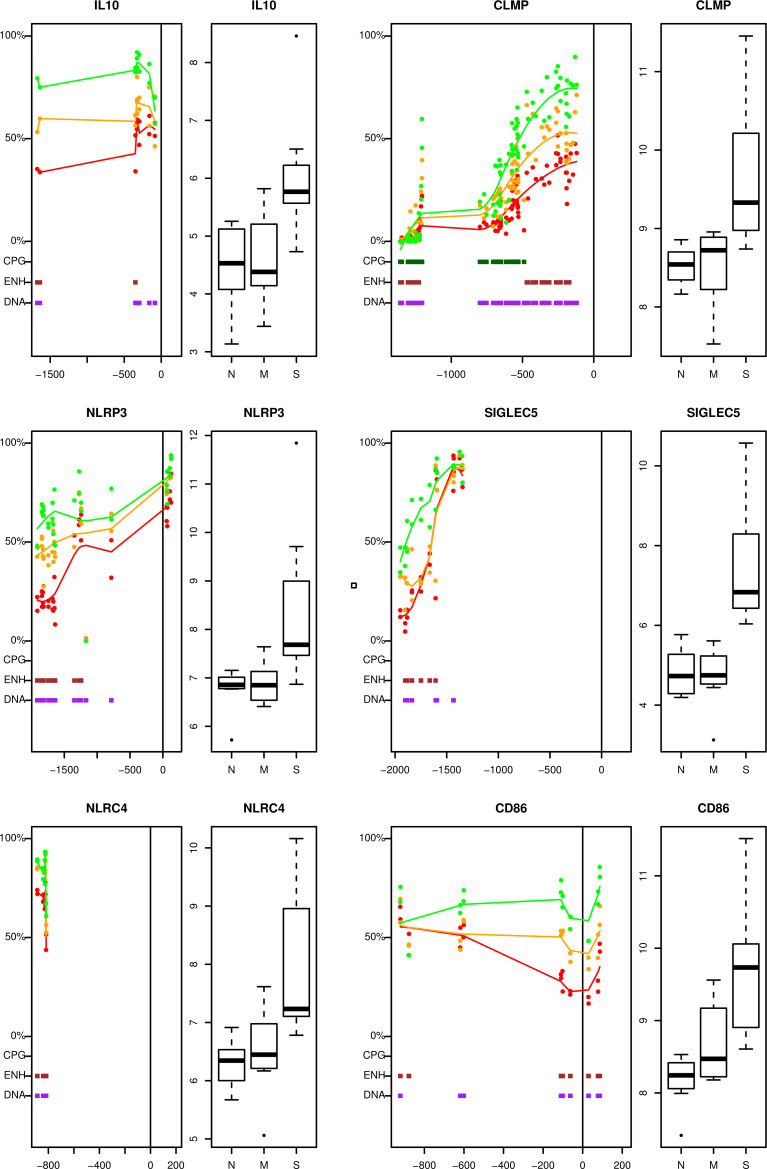
Selection of the most specifically expressed and hypo-methylated genes in severe ulcerative colitis. On the left of each individual illustration the differences in relative methylation levels between normal samples (green), mild UC (orange) and severe UC (red) is shown. Red, green, and orange lines represent the mean relative methylation for severe UC, mild UC and normal samples. The transcription start-site (TSS) is indicated as a vertical line. The x axis is numbered relative to the transcription start site, where minus indicated number of base pairs downstream for TSS (200 bp), and positive number of base pairs upstream from TSS (up to 2000 bp). UCSC genome browser mapped CPG sites (CPG) indicated in dark green, enhancer sites (ENH) indicated in brown, and DNAse1 sites (DNA) indicated in purple. On the right, boxplots of DESEQ2 log2 normalised values for the gene of interest in normal control (N), mild UC (M) and severe UC (S) are shown. Genes are indicated: interleukin 10 (IL10), CXADR- like membrane protein (CLMP), NLR family pyrin domain containing 3 (NLRP3), sialic acid binding Ig like lectin 5 (SIGLEC5), NLR family CARD domain containing 4 (NLRC4) and T-lymphocyte activation antigen CD86 (CD86).

PCA of global DNA methylation data (methPCA) depicts relative methylation counts [1–100%] for over 9 million cytosine positions for the whole genome of all patient samples, severe UC, mild UC, and normal controls (Fig 1B). The methPCA revealed a distinction between the patient groups along the first component with 33% explained variance. Severe UC samples showed a clear separation from both mild UC and control samples along the second component with an explained variance of 11%. Sample (#10) representing a mild phenotype of UC in the methPCA appeared to be an outlier, as a lower sequencing coverage was observed compared to all the other samples (Fig 1B). This was also the case for one normal sample (#21). These outliers were not removed from the dataset since the transcriptomic data of these samples did not show the same tendency. Further analysis revealed that 34, 8% of all significantly DEGs correlated with DNA methylation. Limiting correlation to r < -0.6 resulted in a total of 79 genes of which 77, 2% were hypo-methylated (n = 61) (Table 2 and S3 Table) and 22, 8%, were hyper-methylated (n = 18) (Table 3 and S3 Table). Approximately, 9% of the correlating genes showed DNA methylation at CpG sites in the neighbourhood of the transcription start site (TSS), whereas the remaining 91% of the genes showed methylation at *cis*-acting elements like enhancers and DNAse1 (Fig 3). This is somewhat different for mild UC, where approximately 30% of the genes showed DNA methylation at CpG sites and the remaining 70% of genes showed methylation at *cis-*acting elements [18]. There exists no common opinion on how many methylation sites are necessary for transcription regulation. That’s why correlation analysis was applied in this study. These observed changes nevertheless correlated well with expression changes but cannot explain the underlying molecular events that may cause the transcriptional changes [35]. Complete lists of methylated DEGs correlating with transcription and the respective profiles are depicted in S3 Table, S1 Fig and Fig 3.

**Table 2:** Hypo-methylated genes in treatment-naïve severe ulcerative colitis (UC).

Gene symbol	log2 FC >1.0 transcription	#c	% methyl	SD methyl
ADGRE3	1,55	82	9,92	25,04
ANGPTL2	1,02	114	12,71	18,31
C3AR1	1,91	27	12,06	23,4
CARD6	1,19	20	25,09	4,86
CASS6	1,13	72	14,72	15,65
CD300A	1,29	94	9,54	23,5
CD300E	3,15	30	12,09	19,97
CD48*	1,06	36	15,17	8,37
CD53	1,41	37	12,03	15,32
CD86	1,21	27	14,53	12,22
CD93	1,60	102	14,18	22,72
CFP	1,37	69	13,67	17,7
CLMP	1,35	213	11,13	16,77
CSF2RB	1,44	59	7,06	25,37
CSF3R	3,42	89	13,10	19,6
CST7	1,79	39	11,59	8,4
CTSK	1,59	41	8,98	17,65
CXCR2	2,42	48	8,67	22,98
DNAH17	1,35	90	6,79	15,57
DOK3*	1,31	107	7,22	21,64
FAM124B	1,15	71	19,16	6,2
GNAI2	1,08	109	5,02	18,99
GPSM3	1,18	54	4,29	35,51
IL10*	1,77	27	8,18	9,56
IL18R1*	1,19	36	6,91	15,51
IL1RN	2,98	67	7,36	18,99
ITGB2	1,23	98	20,99	10,13
ITPRIP	1,34	47	26,95	12,64
LAIR1	1,01	116	9,22	25,66
LILRA1	2,52	85	9,66	13,85
LILRB1	1,59	87	8,00	24,64
LILRB2	1,38	74	25,59	23,24
LINC00877	1,06	94	6,15	16,06
LST1	1,55	40	14,70	21,09
MYO1G	1,31	69	19,29	12,31
NFE2	2,93	40	14,94	9,4
NKG7	1,06	61	13,37	28,59
NLRC4	1,74	27	11,95	12,08
NLRP12	3,45	67	18,19	14,84
NLRP3	1,16	70	17,31	21,24
P2RY13	1,25	15	8,88	21,45
PLEKHO1	1,05	50	21,61	6,5
PPP1R18	1,43	147	11,27	13,71
PTPRC*	1,08	18	13,83	17,35
RHOH	1,10	45	21,01	4,74
SCARF1	1,45	97	19,1	23,68
SELPLG	1,38	78	11,57	6,3
SEMA4A	1,23	47	20,45	8,21
SIGLEC5	2,27	54	8,02	30,56
SLA	1,29	35	20,31	6,25
SLAMF1*	1,29	44	15,47	9,16
SLAMF7*	1,09	21	9,93	31,92
SLAMF8	1,60	46	11,27	20,1
SNX20	1,05	48	22,87	19,76
SPARC	1,76	72	13,47	23,71
SPI1	1,43	41	10,81	26,34
TIE1	1,58	52	9,44	14,43
TNFSF14	1,02	122	13,04	21,64
TNFSF8*	1,16	71	12,16	19,28
TREML2	1,72	48	7,52	25,03
WARS	1,25	31	3,28	9,94

#c indicates number of methylated cytosines; % methyl indicates % difference of DNA methylation severe UC vs. mild UC; SD indicates standard deviation; all results shown with p< 0.05.

*indicates IBD susceptibility genes.

**Table 3:** Hyper-methylated genes in treatment- naïve severe ulcerative colitis (UC).

Gene symbol	log2 FC >1.0 transcription	#c	% methyl	SD methyl
C2orf82	-1,24	54	-21,84	14,36
C2orf88	-1,22	13	-5,89	12,73
CES2	-1,08	293	-4,39	12,17
DRAIC	-1,43	45	-20,47	8,71
ENTPD5	-1,13	28	-22,12	7,49
MAGIX	-1,13	107	-19,44	12,01
MMP28	-1,32	122	-3,99	20,58
NGEF	-1,11	42	-15,9	14,13
P3H2	-1,26	21	-2,41	17,45
PFKFB2	-1,02	163	-4,59	29,1
PPARGC1A	-1,51	42	-6,09	15,36
PRKG2	-1,42	100	-21,46	11,761
PVRL3	-1,04	30	-22,71	10,86
SLC22A18AS	-1,45	115	-15,49	16,66
SLC51B	-1,17	33	-19,92	8,45
TMEM72	-1,61	58	-14,90	13,3
TRPM4	-1,07	109	-30,69	9,94
UGT1A8	-1,77	31	-13,65	16,15

#c indicates number of methylated cytosines; % methyl indicates % difference of DNA methylation severe UC vs. mild UC; SD indicates standard deviation; all results shown with p< 0.05.

The major DNA methylation event in treatment-naïve severe UC seems to be hypo-methylation. It is intriguing that approximately 80% of all significant DEGs which correlated to DNA methylation were hypo-methylated in severe UC compared to mild UC (S3 Table). A global hypo-methylation of mucosal DNA in UC compared to normal controls has been reported earlier and it has been suggested that these epigenetic changes in the mucosa might contribute to cancer development [36]. It is well-known that severe inflammation results in an impairment of the epithelial mucosal layer which is followed by diffusion of commensal bacteria and significantly increase of leukocyte infiltration into the gut [37]. This is confirmed by hypo-methylation of leukocyte–specific transcript 1 (LST1), leukocyte associated immunoglobulin-like receptor 1 (LAIR1), sialic acid binding Ig-like lectin 5 (SIGLEC5), and leukocyte surface antigen CD53 (CD53) (Table 1), decreased fractions of epithelial cells and increased fractions of neutrophils, T cells, and monocytes during severe UC compared to mild UC (Fig 2). Fractions of immune cell subtypes, stroma, and epithelial cells on the basis of the gene expression data using cell deconvolution, showed that severe UC differed from mild UC by increased proportions of monocytes (p = 0.03) and neutrophiles (p = 0.02) and a loss of stroma (p = 0.001) and epithelial cells (p = 0.001). No significant differences were found between mild UC and normal controls (S4 Table). In addition, pathway enrichment of significantly and differentially DNA methylated genes revealed their involvement in two pathways, neutrophil degranulation, and immuno-regulatory interaction between lymphoid and non-lymphoid cell (Table 4). For both pathways only hypo-methylated genes could be annotated.

**Table 4:** Reactome enriched pathways of methylated genes in severe ulcerative colitis (UC).

Enriched pathways for severe UC vs. mild UC, p_adj._ < 0.05	Gene symbol
Neutrophil degranulation (innate immune system)	ADGRE3, C3AR1, CD53, CD93, CD300A, CFP, CXCR2, DOK3*, ITGB2, LAIR1, LILRB2, PTPRC*, SIGLEC5
Immuno-regulatory interactions between a Lymphoid and a non-Lymphoid cell (adaptive immune system)	CD300A, CD300E, ITGB2, LAIR1, LILRA1, LILRB1, LILRB2, SIGLEC5, SLAMF7*, TREML2

*indicates IBD susceptibility genes.

Seven IBD susceptibility genes were identified, B-lymphocyte activation marker BLAST1 (CD48), interleukin 10 (IL10), protein tyrosine phosphatase receptor type C (PTPRC), Slam family members (SLAMF7 & SLAMF1), TNF superfamily member 8 (TNFSF8), and docking protein 3 (DOK3) which all were hypo-methylated and up-regulated in severe UC (S2 Table). The hypo-methylation of CD48, IL-10 and PTPRC has not been observed for mild UC [18] and seem to be a specific feature of severe UC. It is interesting to note that only four genes of the top DEGs were differentially methylated, colony stimulating factor 3 receptor (CSF3R) and NLR family pyrin domain containing 12 (NLRP12) which are hypo-methylated (Table 2), and transmembrane protein 72 (TMEM72) and UDP glucuronosyltransferase family 1 member A8 (UGT1A8) which are hyper-methylated (Table 3). UGT1A8 has been found to be hyper-methylated in mild UC in an earlier report [18]. A selection of the most specifically methylated DEGs in severe UC clearly show the differences in relative methylation levels for severe UC (S), mild UC (M), and normal samples (N) upstream from the transcription start site (TSS) and indicate UCSC genome browser mapped CpG sites and cis- elements, like enhancers and Dnase1 sites (Fig 3) The boxplots of DESEQ2 log2 normalised values for interleukin 10 (IL10), CXADR- like membrane protein (CLMP), NLR family pyrin domain containing 3 (NLRP3), sialic acid binding Ig like lectin 5 (SIGLEC5), NLR family CARD domain containing 4 (NLRC4) and T-lymphocyte activation antigen CD86 (CD86) showed a clear correlation of DNA methylation status and transcription, thereby clearly indicate specific alterations through hypo-methylation of these genes in severe UC.

The observed hypo-methylation of the NLR family pyrin domain containing NOD-like receptor family members (NLRP3 and NLRP12) in severe UC may maintain intestinal homeostasis and adapt responses against multiple intestinal insults [37–39]. In response to inflammation, hypo-methylation of NRLP inflammasomes may confer anti-inflammatory signals in order to improve severe colitis and to prevent further damage, thereby acting as a defence mechanism to mitigate inflammation. The NLRP3 inflammasome is expressed in both, gut epithelial (IEC) and immune cells (DCs, macrophages, B cells) and may therefore governing the balance of intestinal homeostasis depending on specific cell populations [40–42]. Hypo-methylation of NLRP12 and NLRC4 may regulate gut microbiota in order to supress intestinal inflammation and subsequent intestinal damage in severe UC [43–47]. It is interesting to note that the cassette of NLRs in severe UC is different from those found in mild UC, and that PRRs like Toll-receptors (TLR1, TRL2, TRL4, TRL6, TLR8 and TLR9) are all up-regulated, but not hypo-methylated in severe UC (S1 Table) [17, 18].

A similar interplay between the innate and adaptive immune system can be implied for IL10, a cytokine which has pleiotropic effects in immuno-regulation and inflammation which is expressed and hypo-methylated in severe UC but not in mild UC [17, 18, 48]. IL10 expression during severe UC might counteract excessive inflammatory immune responses by downregulating the function of antigen presenting cells (APCs), thus providing feedback regulation for pro-inflammatory T cells [49–52]. The increased expression of IL10 produced by T cells may also play a role in mediating tolerance against commensal bacteria, whereas the expression of IL10 in peripheral tissues may lead to down-modulation of the immune response. It has been recently shown that macrophages in the lamina propria preferentially induce IL10 producing cells while DCs promote the generation of Th17 cells [53–55]. It can be therefore believed that hypo-methylation and increased expression of IL10 counteracts severe inflammatory signals and aims to dampen severe intestinal inflammation. During severe UC, hypo-methylation of IL10 might also induce tolerogenic DCs that exhibit high expression of co-stimulatory molecules combined with highly expressed inhibitory leucocytes immunoglobulin like receptors (LILRs) and secrete IL10 resulting in the induction of T cells with regulatory capacities (Tregs) [56]. Many of these receptors (LILRA1, LILRB1 and LILRB2) are more hypo-methylated in severe UC than in mild UC (Table 2) [18]. LILRB receptors expressed on immune cells bind to MHC class I molecules on antigen-presenting cells (APCs, DCs) and transduces a negative signal that inhibits stimulation of an immune response. This suggests a role of these receptors in balancing the inflammatory response in face of bacterial infection in severe UC. Although other cells such as macrophages and B cells are also able to present antigens via MHC, DCs are the only cell type to activate naïve T cells and to induce antigen specific immune responses in all adaptive immune cells [57, 58]. An increase of cell fractions in monocytes, neutrophils, T-cells, B-cells and stroma cells were observed in all severe UC samples, whereas the cell fraction of epithelial cells was significantly decreased in all severe UC samples compared with mild UC (Fig 2 and S4 Table).

In concordance with enhanced fractions of T cells in severe UC (Fig 2) increased expression of CD86, a coactivator DC marker involved in T cell activation during microbial infection was observed in severe UC [59]. Other hypo-methylated genes of relevance for the defence of severe inflammation is CXADR-like membrane protein (CLMP) which stabilizes the gut vascular barrier localized between endothelial and epithelial cells in junctional complex involved in cell adhesion and which is required for normal intestinal homeostasis and development (Fig 2 and Table 2) [60].

All the above discussed defence mechanisms might prevent a complete collapse of a functional mucosal barrier during severe inflammation. It is therefore believed that the increase of protective genes and anti-inflammatory pathways induced by hypo-methylation are defence mechanisms, thereby counteracting and alleviating severe inflammation in the gut. Nonetheless, the study is not without limitations, the sample size used here can be considered low due to low number of patients with a severe UC phenotype, but still show sufficiently separation in the PCA (Fig 1). In addition, due to the heterogeneity of the tissue biopsies it is difficult to account NLRP inflammasomes to specific and distinct cell type and single-cell sequencing might overcome this problem. However, the strength of this study lies within the study design where a treatment-naïve patient group with severe UC have been used in order to compare joint transcriptomic and DNA methylation data from each individual patient. This matching of data reduces the chances of introducing influential variable and inter-individual differences and avoid confounding effects of prior medications while highlighting lasting changes to the regulatory patterns underlying the disease that may be of clinical utility.

## Conclusion

Hypo-methylation of genes with anti-inflammatory character during severe UC implies a functional interplay between the epithelium and lamina propria to mitigate inflammation in the gut. The specifically DNA hypo-methylated genes found for severe UC can potentially be useful biomarkers for determining disease severity and in the development of new targeted treatment strategies for patients with severe UC.

## Supporting information

S1 Fig Hypo-methylated genes in severe ulcerative colitis. On the left of each individual illustration the differences in relative methylation levels between normal samples (green), mild UC (orange) and severe UC (red) is shown. Red, green and orange lines represent the mean relative methylation for severe UC, mild UC and normal samples. The transcription start site (TSS) is indicated as a vertical line. The x axis is numbered relative to the transcription start site, where minus indicated number of base pairs downstream for TSS (200 bp), and positive number of base pairs upstream from TSS The regions upstream (up to 2000 bp). UCSC genome browser mapped CPG sites (CPG) indicated in dark green, enhancer sites (ENH) indicated in brown, and DNAse1 sites (DNA) indicated in purple. On the right, boxplots of DESEQ2 log2 normalised values for the gene of interest in normal control (N), mild UC (M) and severe UC (S) are shown. Genes are indicated by the respective gene symbol.
(PDF)

S1 Table List of significantly differentially expressed genes (DEGs). (XLSX)

S2 Table Differentially expressed IBD susceptibility genes in severe UC. (DOCX)

S3 Table List of DEGs correlating to DNA methylation with r > - 0.6. (XLSX)

S4 Table Comparison of cell population estimates. (XLSX)
