# Supplementary material for: DNA hypo-methylation facilitates anti-inflammatory responses in severe ulcerative colitis
Source: PLoS One. 2021 Apr 1;16(4):e0248905. doi: 10.1371/journal.pone.0248905 (PMC8016308; doi:10.1371/journal.pone.0248905)
Supplement: S1 Fig — On the left of each individual illustration the differences in relative methylation levels between normal samples (green), mild UC (orange) and severe UC (red) is shown. Red, green and orange lines represent the mean relative methylation for severe UC, mild UC and normal samples. The transcription start site (TSS) is indicated as a vertical line. The x axis is numbered relative to the transcription start site, where minus indicated number of base pairs downstream for TSS (200 bp), and positive number of base pairs upstream from TSS The regions upstream (up to 2000 bp). UCSC genome browser mapped CPG sites (CPG) indicated in dark green, enhancer sites (ENH) indicated in brown, and DNAse1 sites (DNA) indicated in purple. On the right, boxplots of DESEQ2 log2 normalised values for the gene of interest in normal control (N), mild UC (M) and severe UC (S) are shown. Genes are indicated by the respective gene symbol. (PDF) [file pone.0248905.s001.pdf]

S4 Fig. Hypo-methylated genes in severe ulcerative colitis. On the left of each individual illustration the differences in relative methylation levels between normal samples (green), mild UC (orange) and severe UC (red) is shown. Red, green and orange lines represent the mean relative methylation for severe UC, mild UC and normal samples.

The transcription start site (TSS) is indicated as a vertical line. The x axis is numbered relative to the transcription start site, where minus indicated number of base pairs downstream for TSS (200 bp), and positive number of base pairs upstream from TSS The regions upstream (up to 2000 bp). UCSC genome browser mapped CPG sites (CPG) indicated in dark green, enhancer sites (ENH) indicated in brown, and DNAse1 sites (DNA) indicated in purple. On the right, boxplots of DESEQ2 log2 normalised values for the gene of interest in normal control (N), mild UC (M) and severe UC (S) are shown. Genes are indicated by the respective gene symbol.

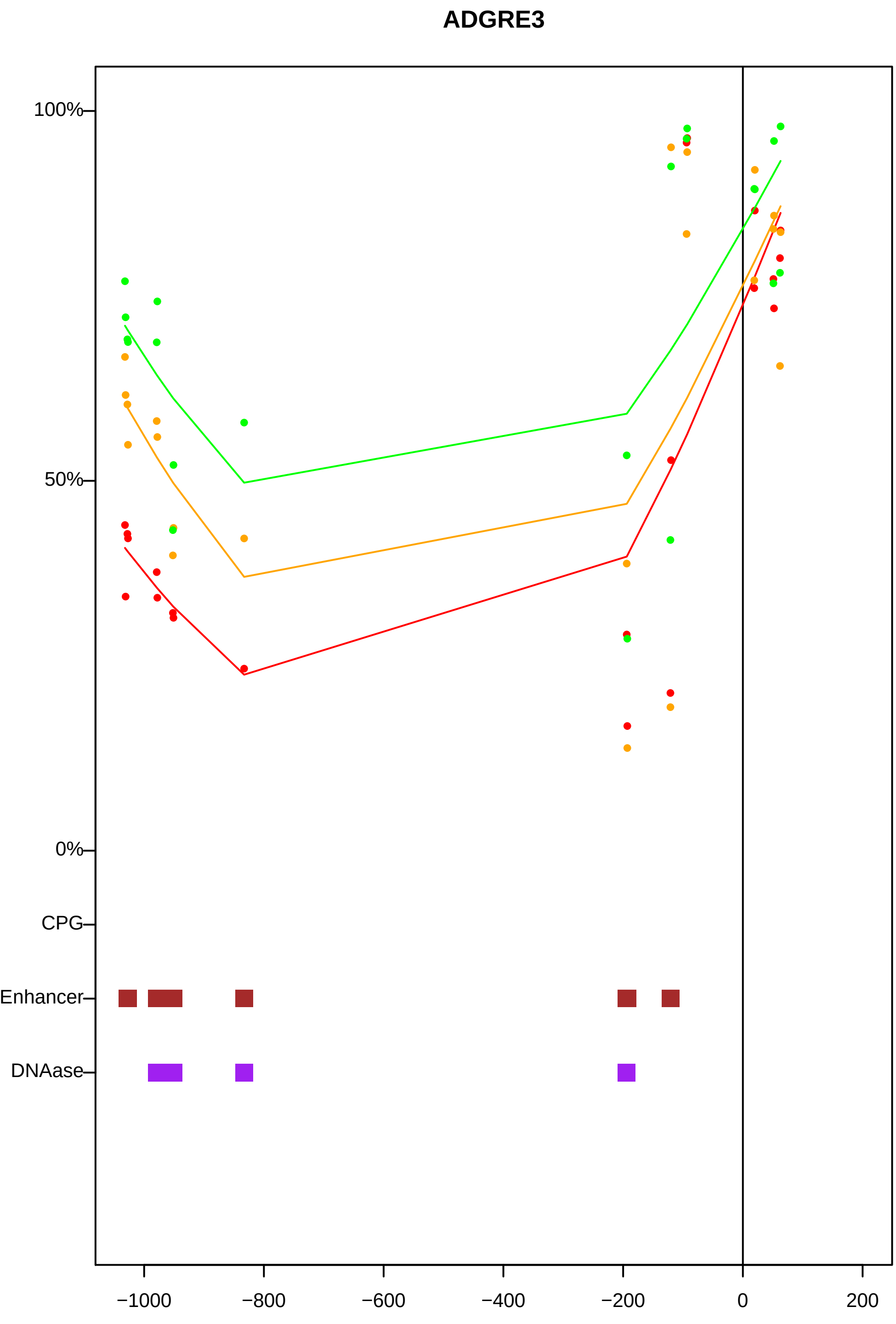
ADGRE3
100%
50%
0%
CPG
Enhancer
DNAase
−1000
−800
−600
−400
−200
0
200

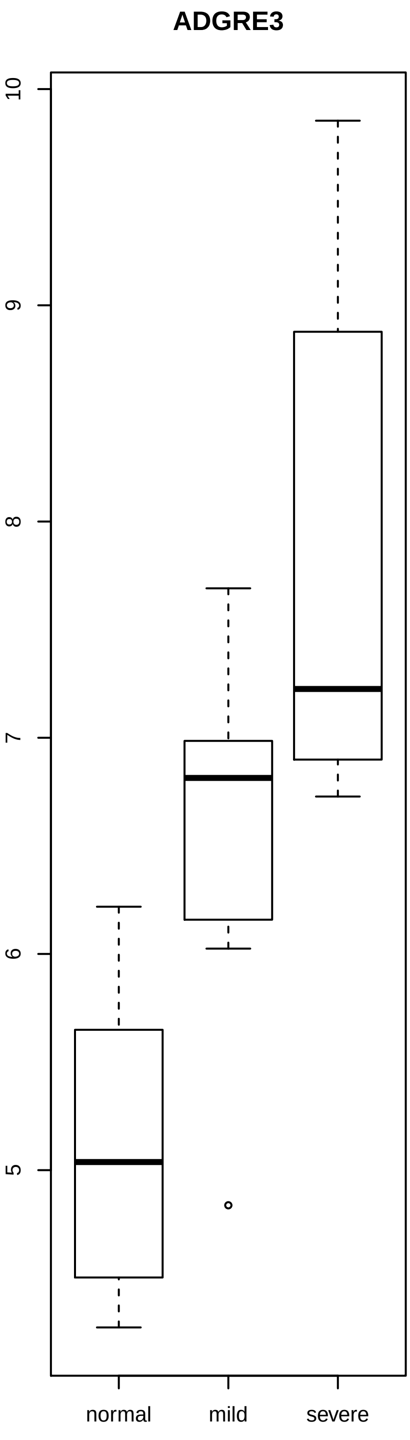
ADGRE3
10
9
8
7
6
5
normal
mild
severe

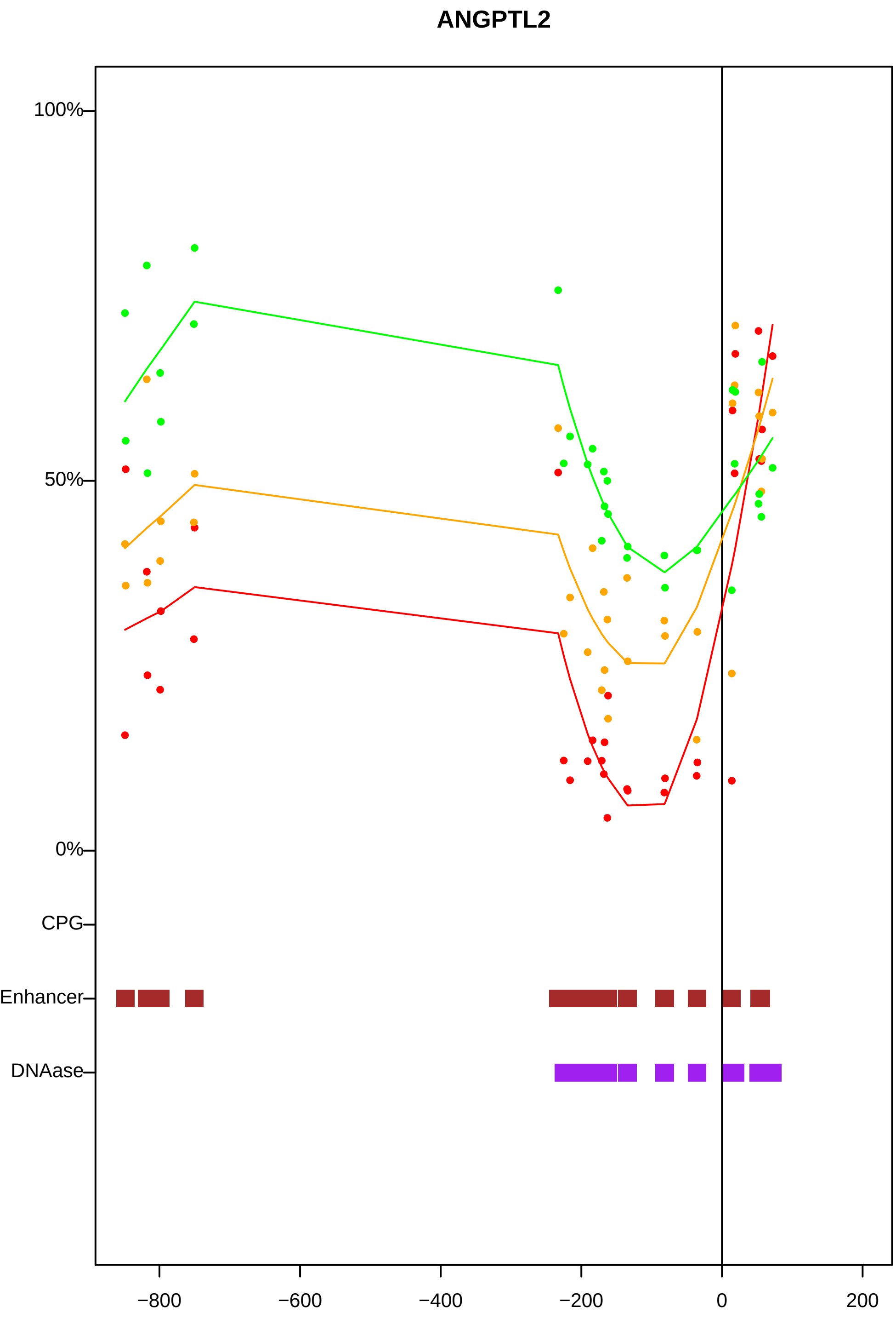

ANGPTL2
100%
50%
0%
CPG
Enhancer
DNAase
−800
−600
−400
−200
0
200

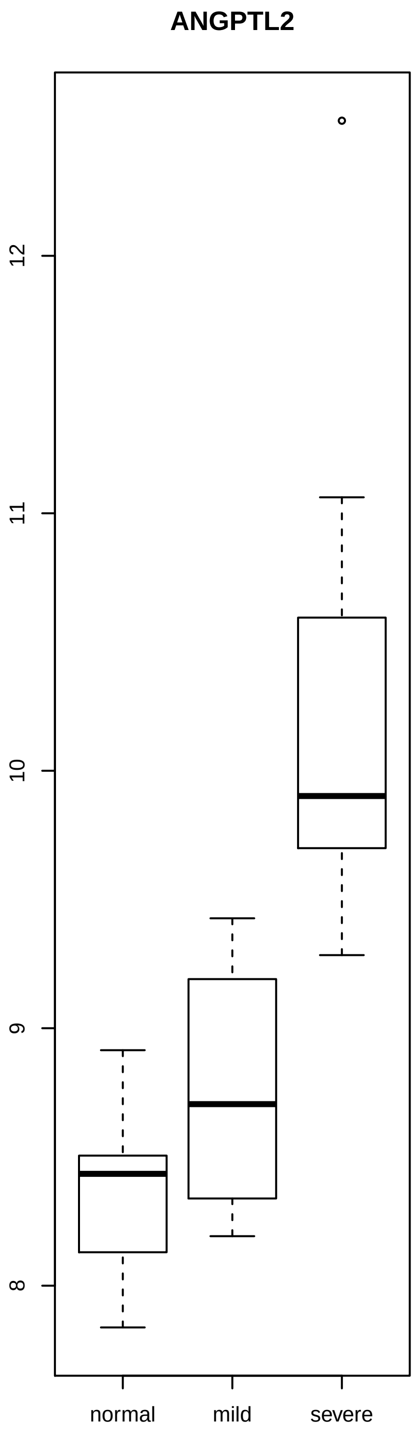

ANGPTL2
12
11
10
9
8
normal
mild
severe

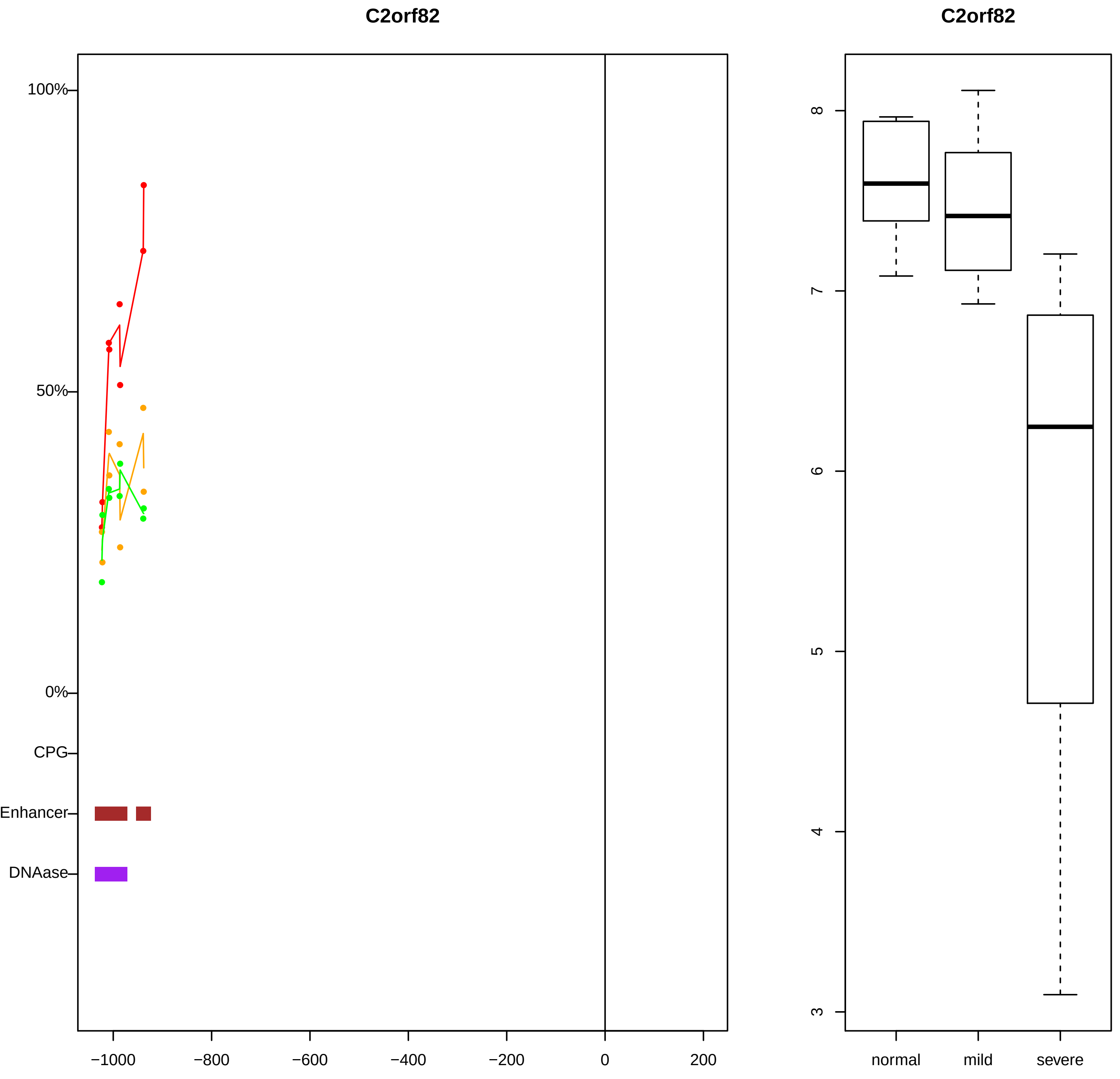

C2orf82
100%
50%
0%
CPG
Enhancer
DNAase
−1000
−800
−600
−400
−200
0
200
C2orf82
8
7
6
5
4
3
normal
mild
severe

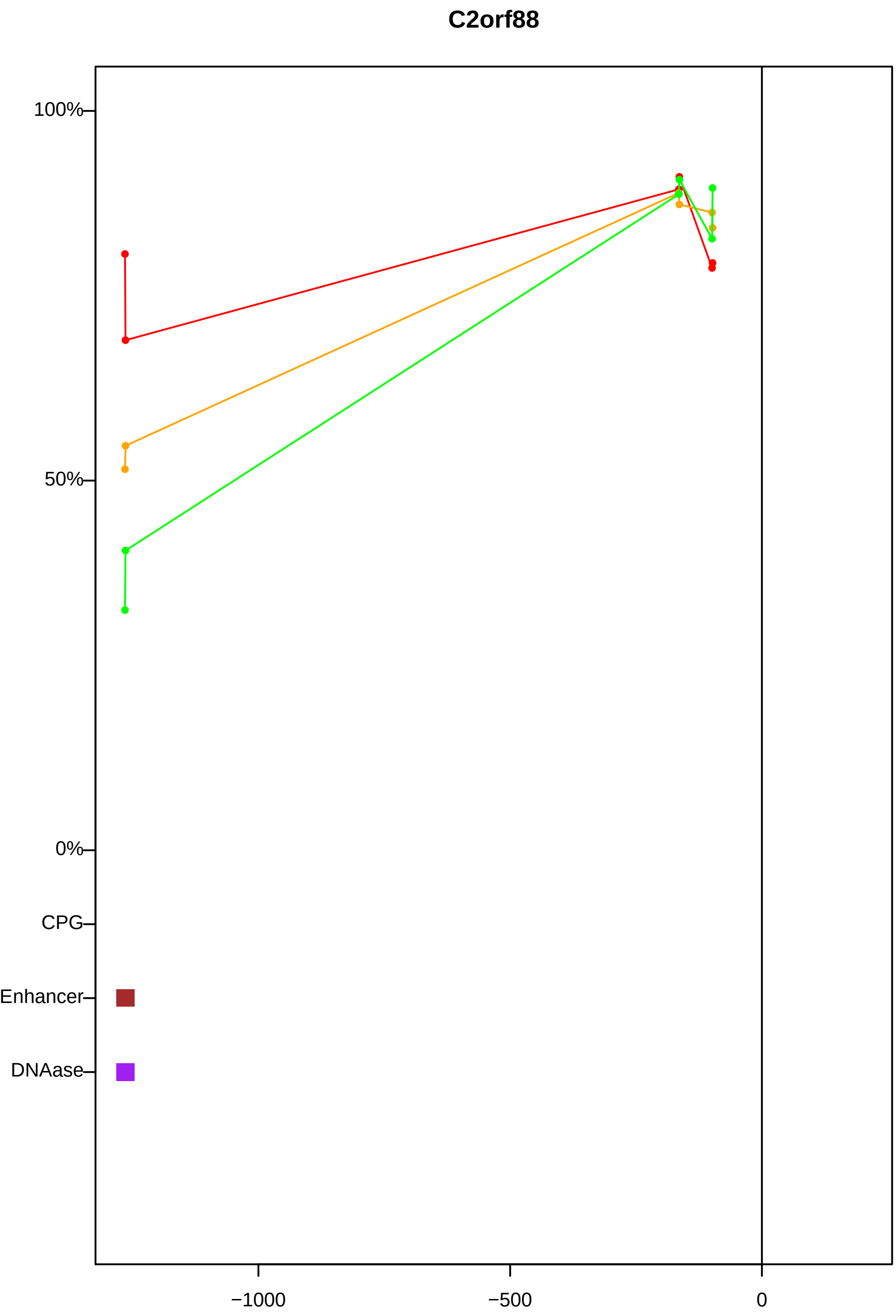

C2orf88
100%
50%
0%
CPG
Enhancer
DNAase
−1000
−500
0

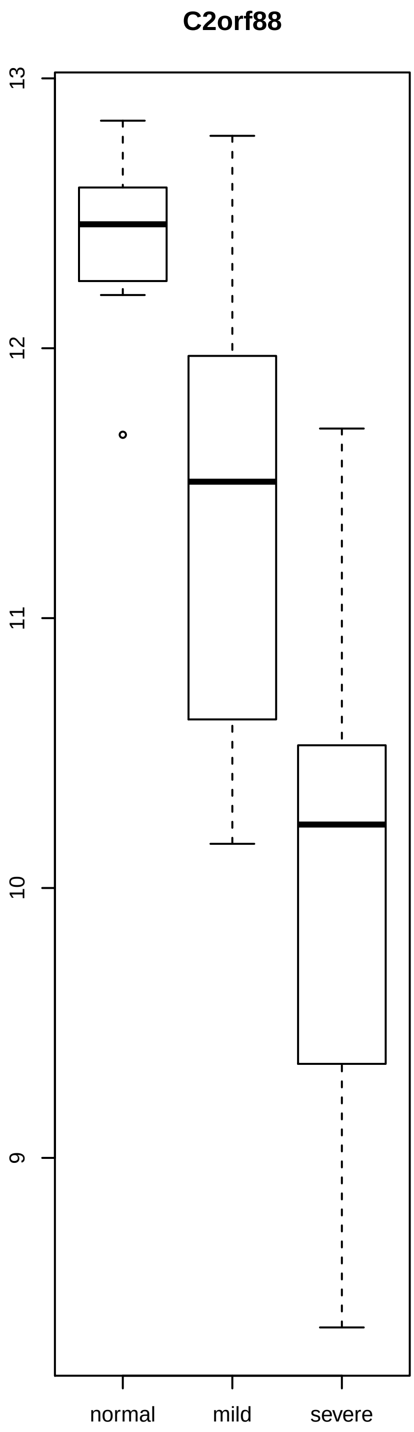

C2orf88
13
12
11
10
9
normal
mild
severe

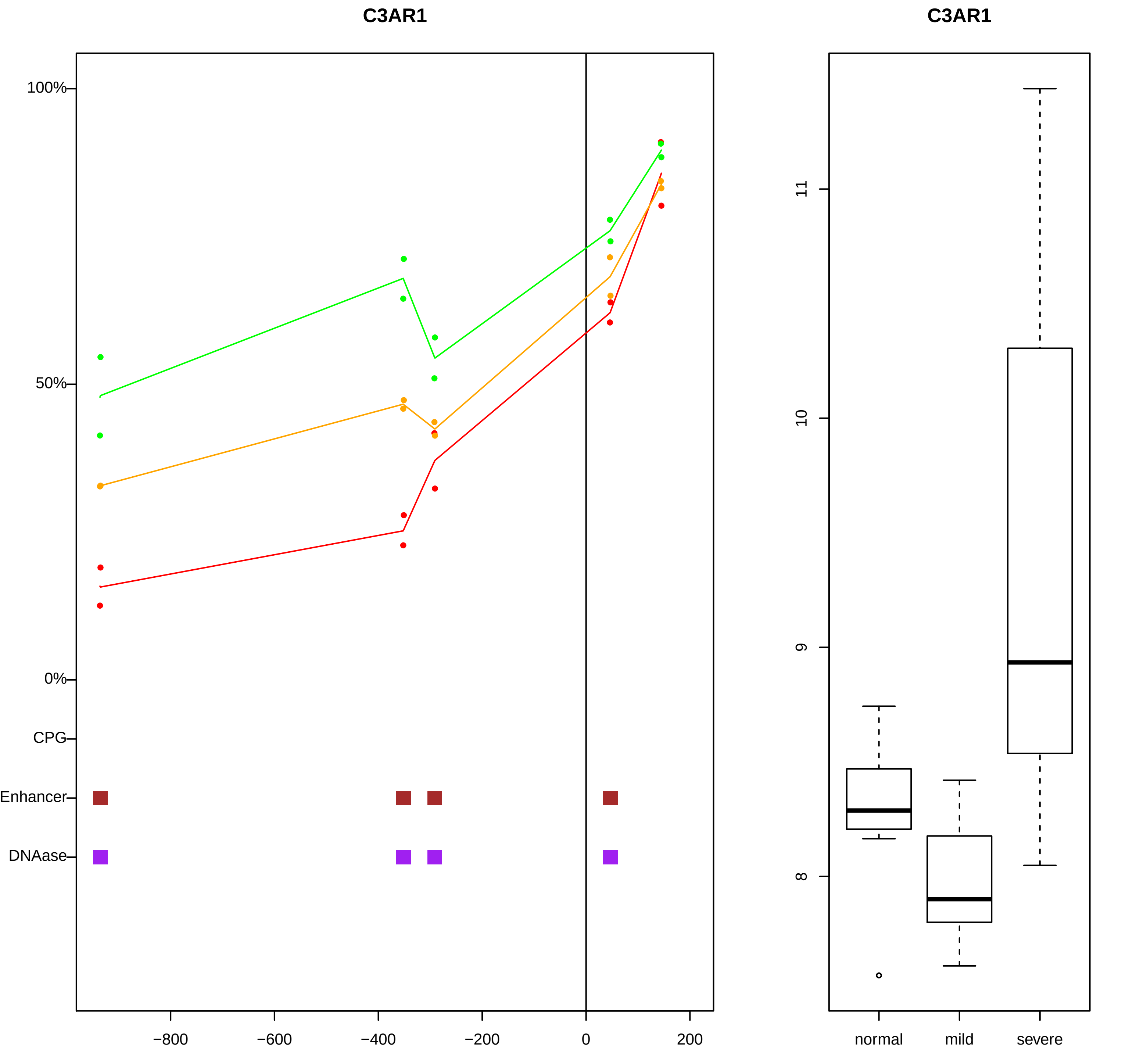

C3AR1
100%
50%
0%
CPG
Enhancer
DNAase
−800
−600
−400
−200
0
200
C3AR1
11
10
9
8
normal
mild
severe

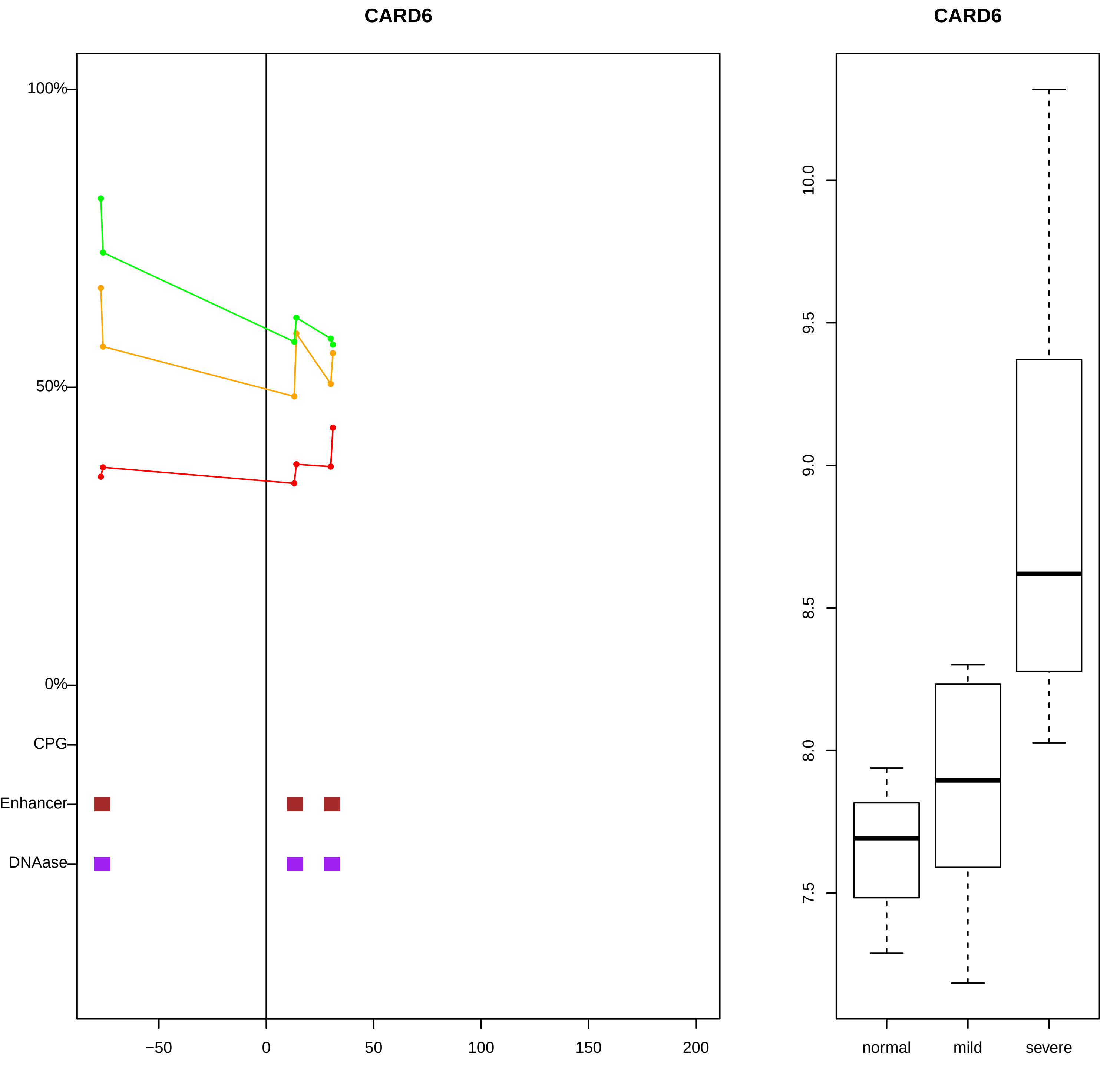
CARD6
100%
50%
0%
CPG
Enhancer
DNAase
−50
0
50
100
150
200
CARD6
10.0
9.5
9.0
8.5
8.0
7.5
normal
mild
severe

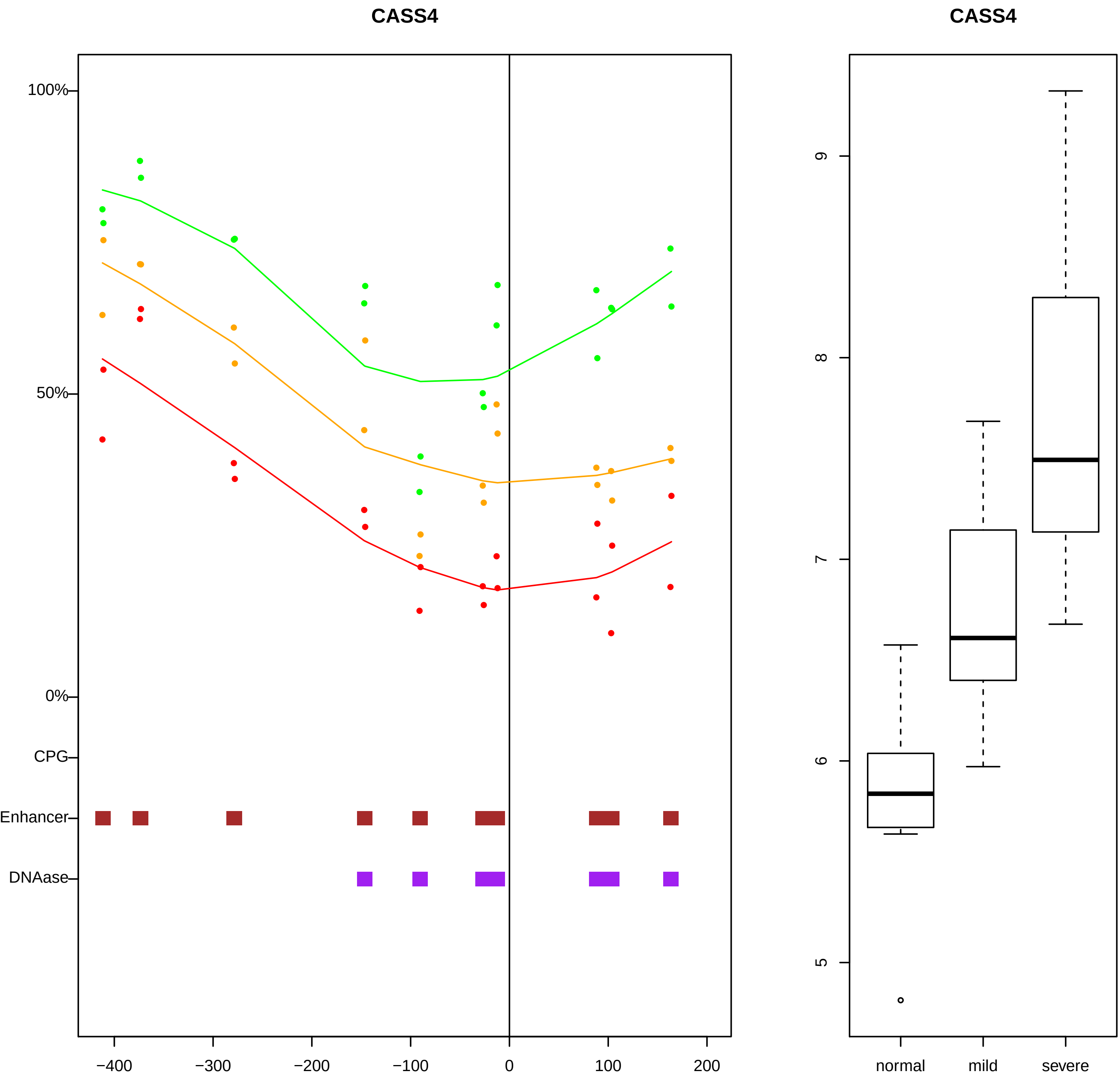
CASS4
100%
50%
0%
CPG
Enhancer
DNAase
−400
−300
−200
−100
0
100
200
CASS4
9
8
7
6
5
normal
mild
severe

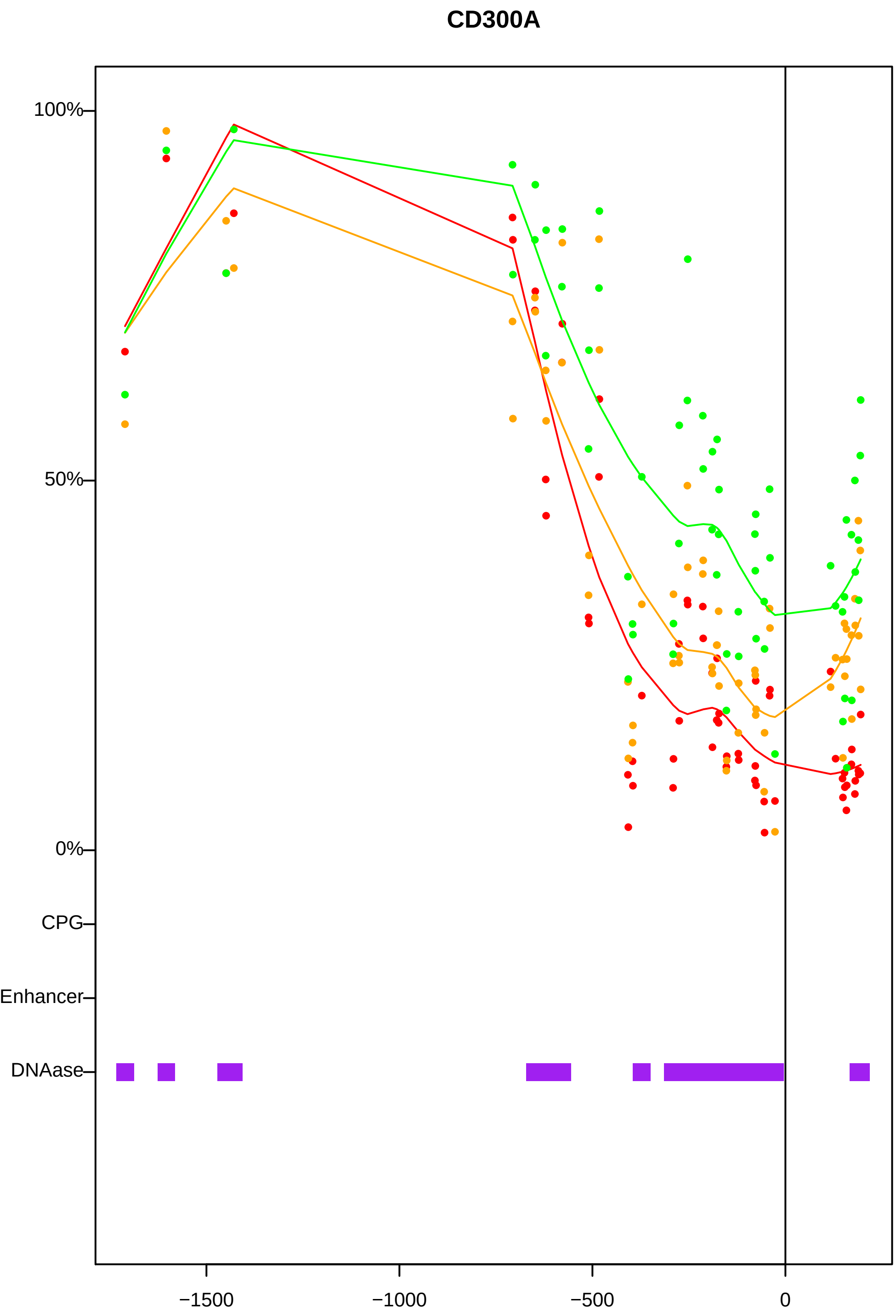
CD300A
100%
50%
0%
CPG
Enhancer
DNAase
−1500
−1000
−500
0
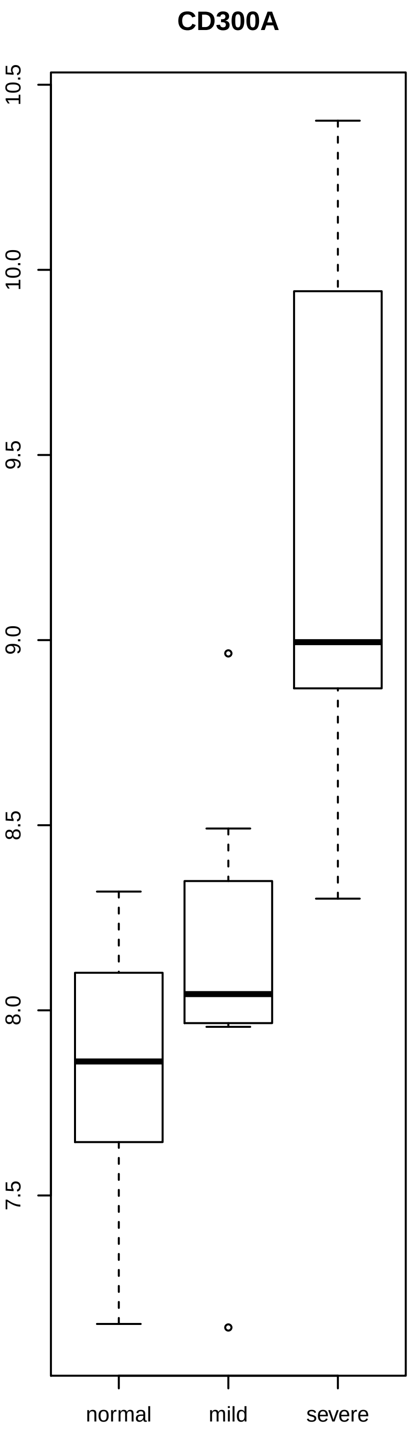
CD300A
10.5
10.0
9.5
9.0
8.5
8.0
7.5
normal
mild
severe

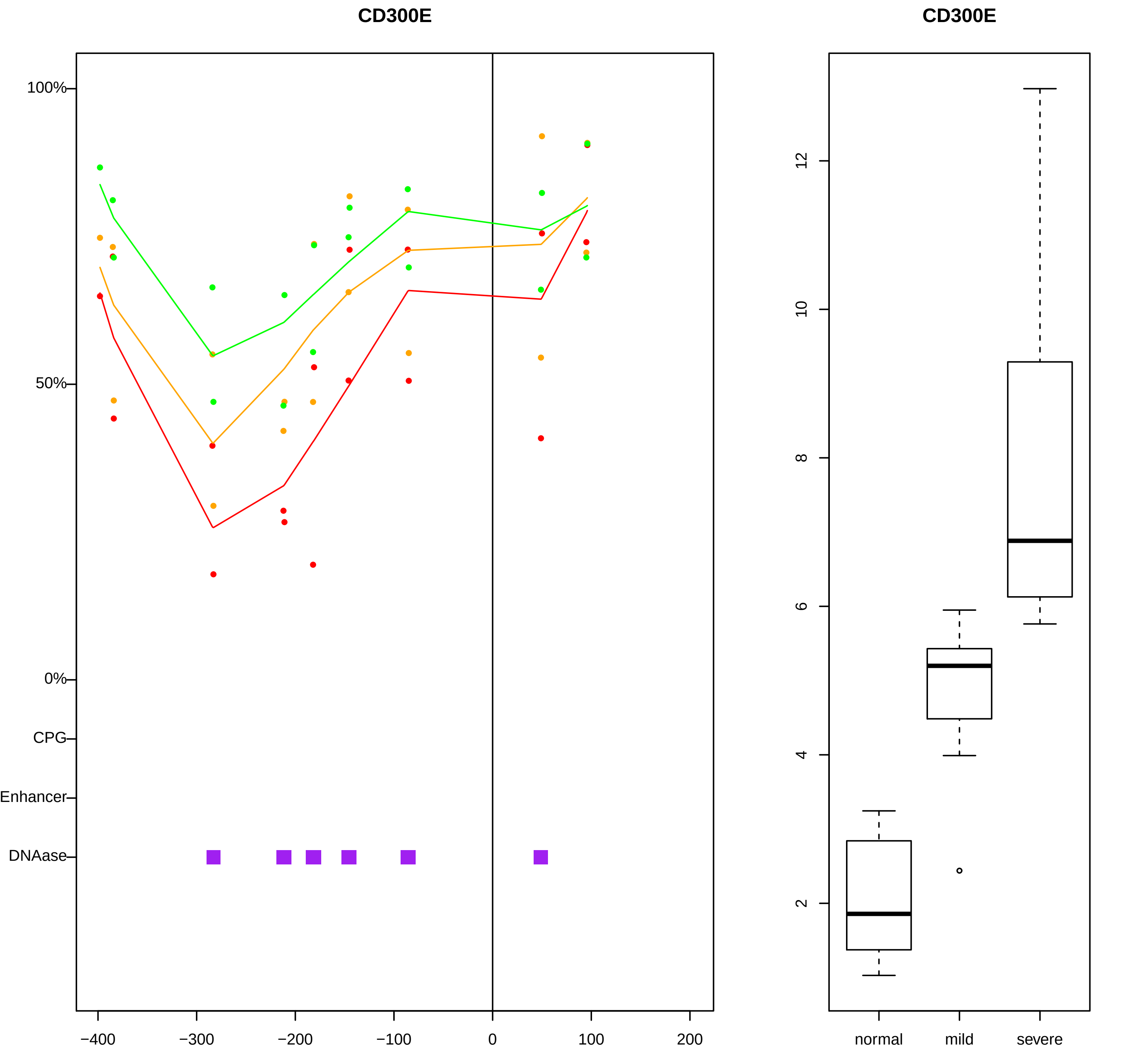
CD300E
100%
50%
0%
CPG
Enhancer
DNAase
−400
−300
−200
−100
0
100
200
CD300E
12
10
8
6
4
2
normal
mild
severe

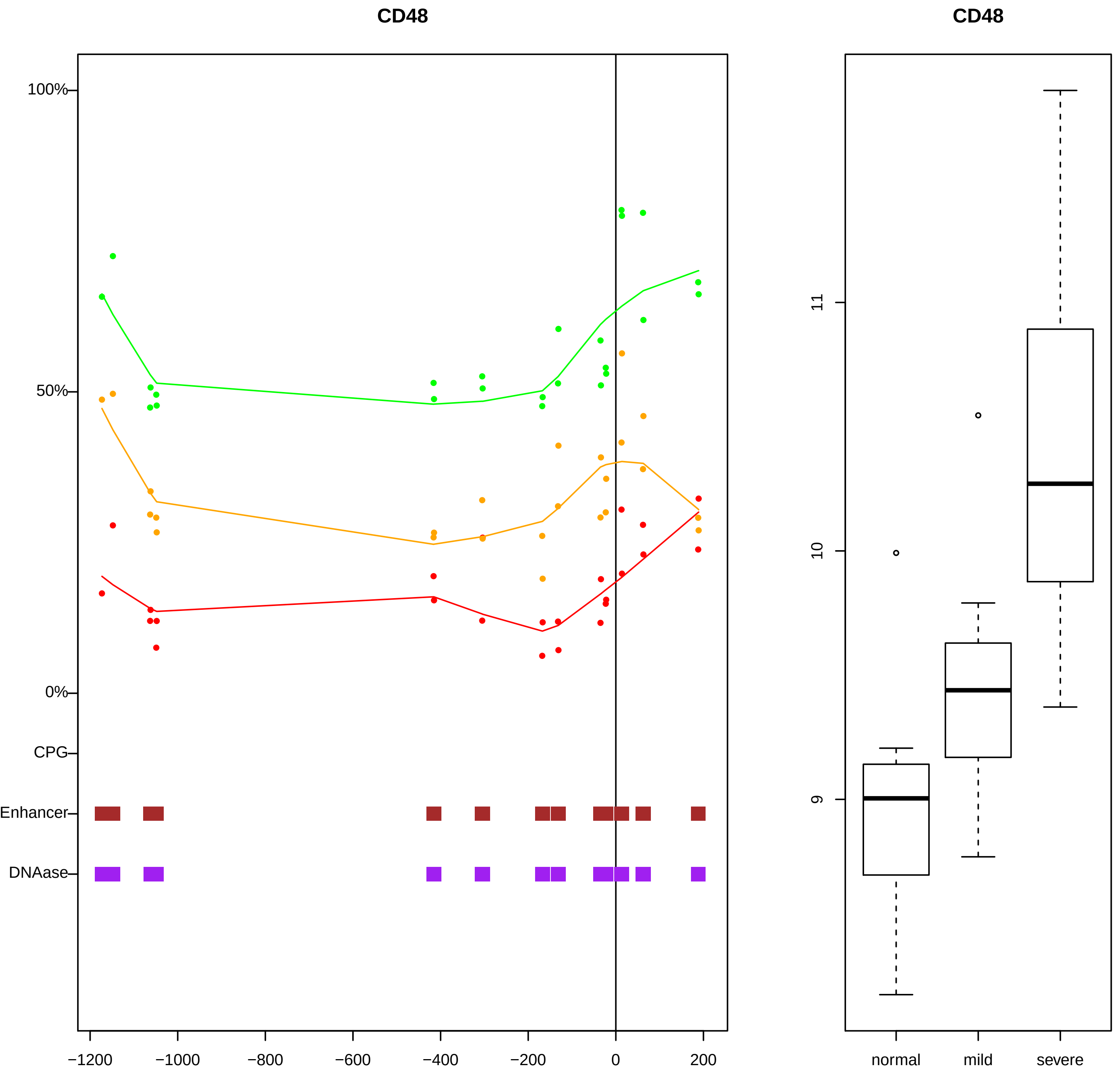

CD48
100%
50%
0%
CPG
Enhancer
DNAase
−1200
−1000
−800
−600
−400
−200
0
200
CD48
11
10
9
normal
mild
severe

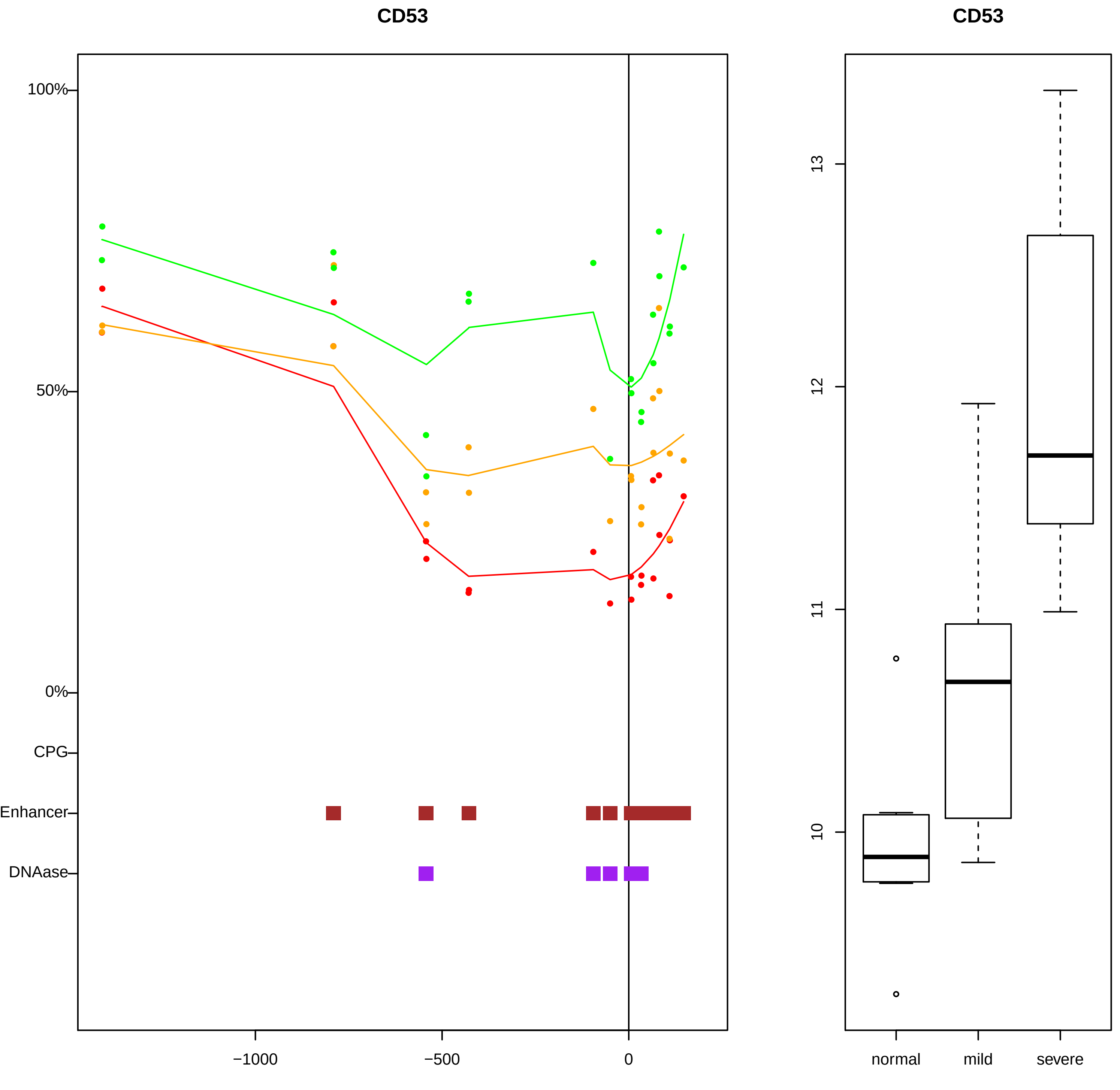
CD53
100%
50%
0%
CPG
Enhancer
DNAase
−1000
−500
0
CD53
13
12
11
10
normal
mild
severe

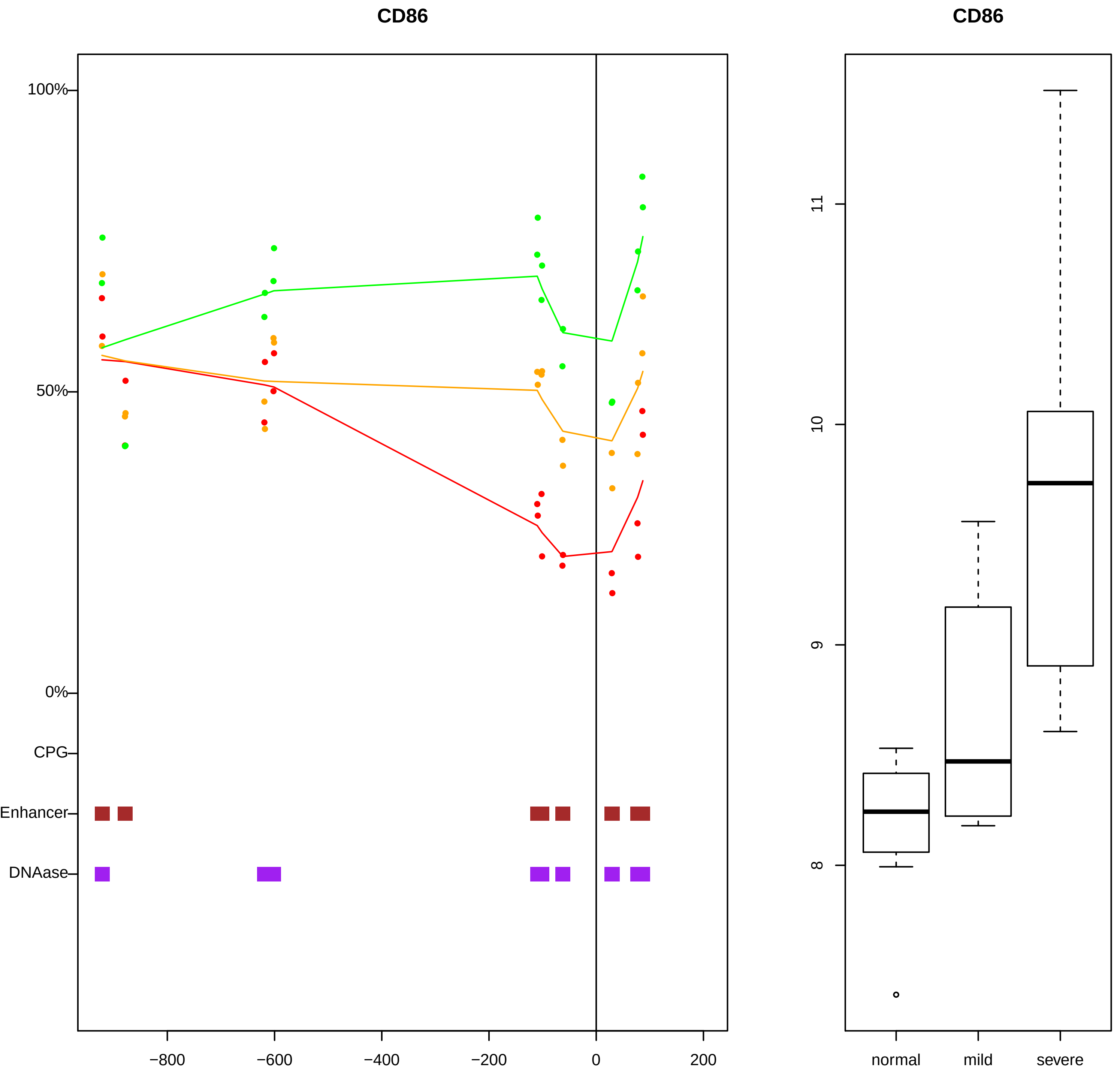
CD86
100%
50%
0%
CPG
Enhancer
DNAase
−800
−600
−400
−200
0
200
CD86
11
10
9
8
normal
mild
severe

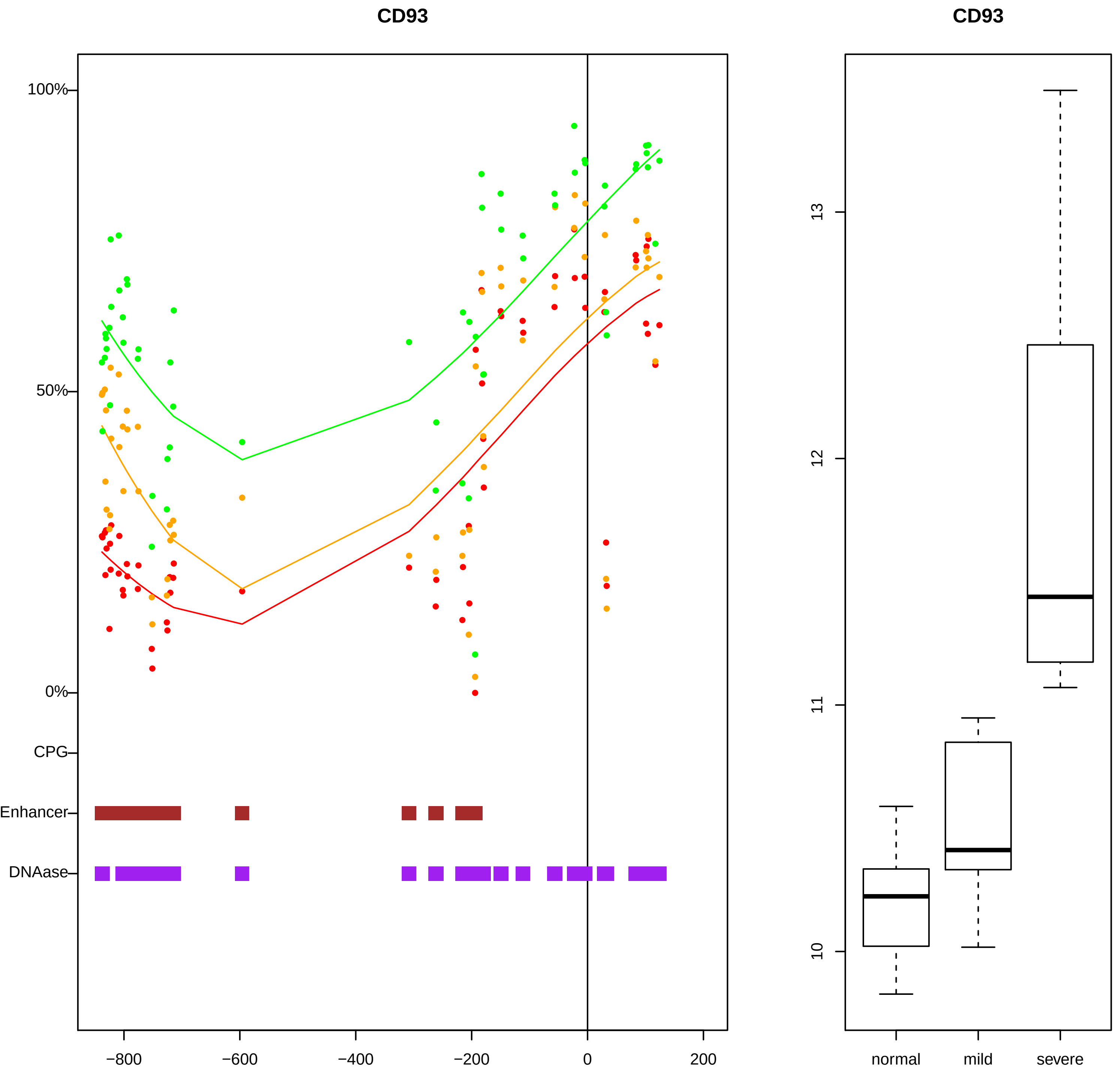
CD93
100%
50%
0%
CPG
Enhancer
DNAase
−800
−600
−400
−200
0
200
CD93
13
12
11
10
normal
mild
severe

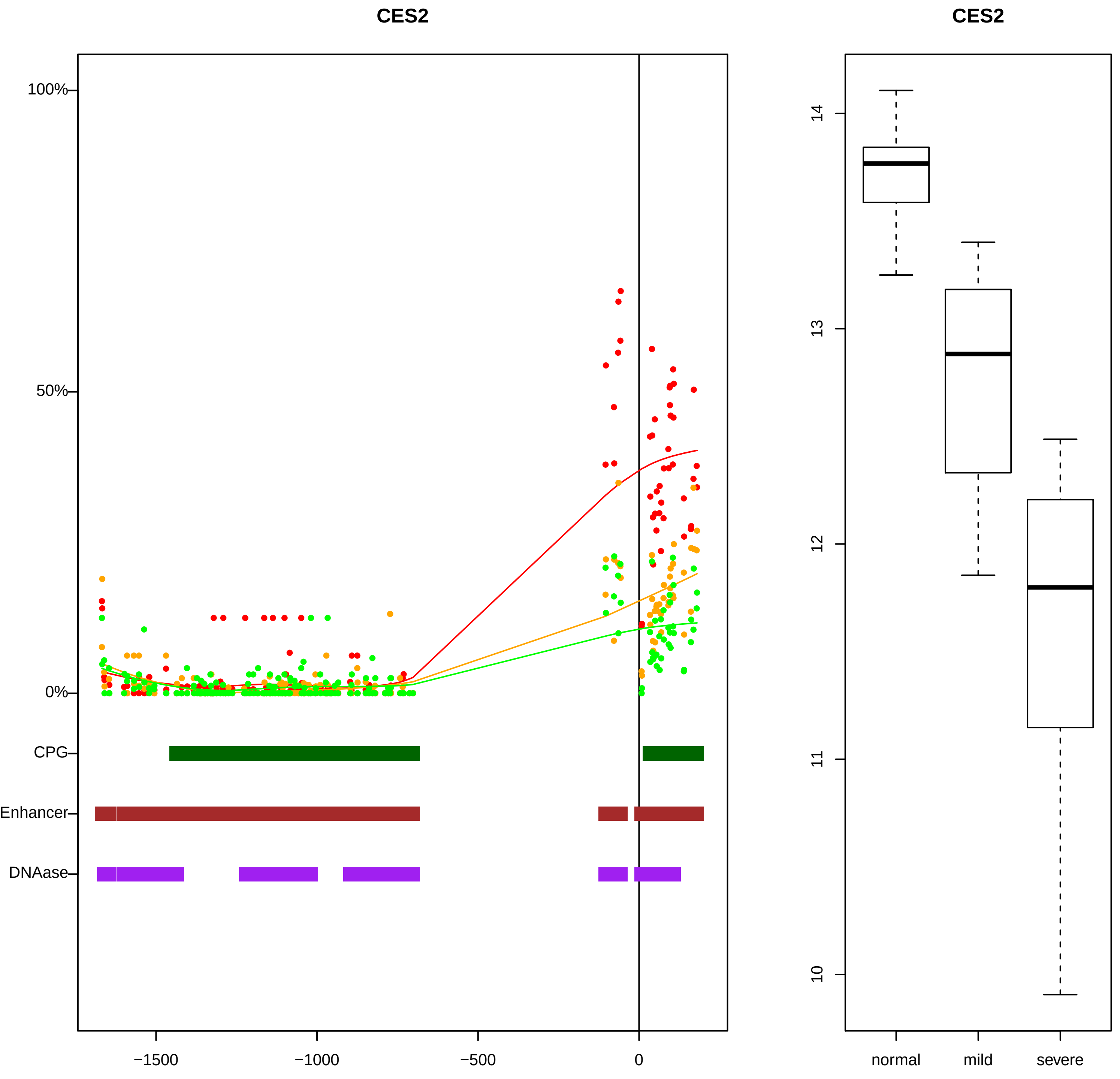

CES2
100%
50%
0%
CPG
Enhancer
DNAase
−1500
−1000
−500
0
CES2
14
13
12
11
10
normal
mild
severe

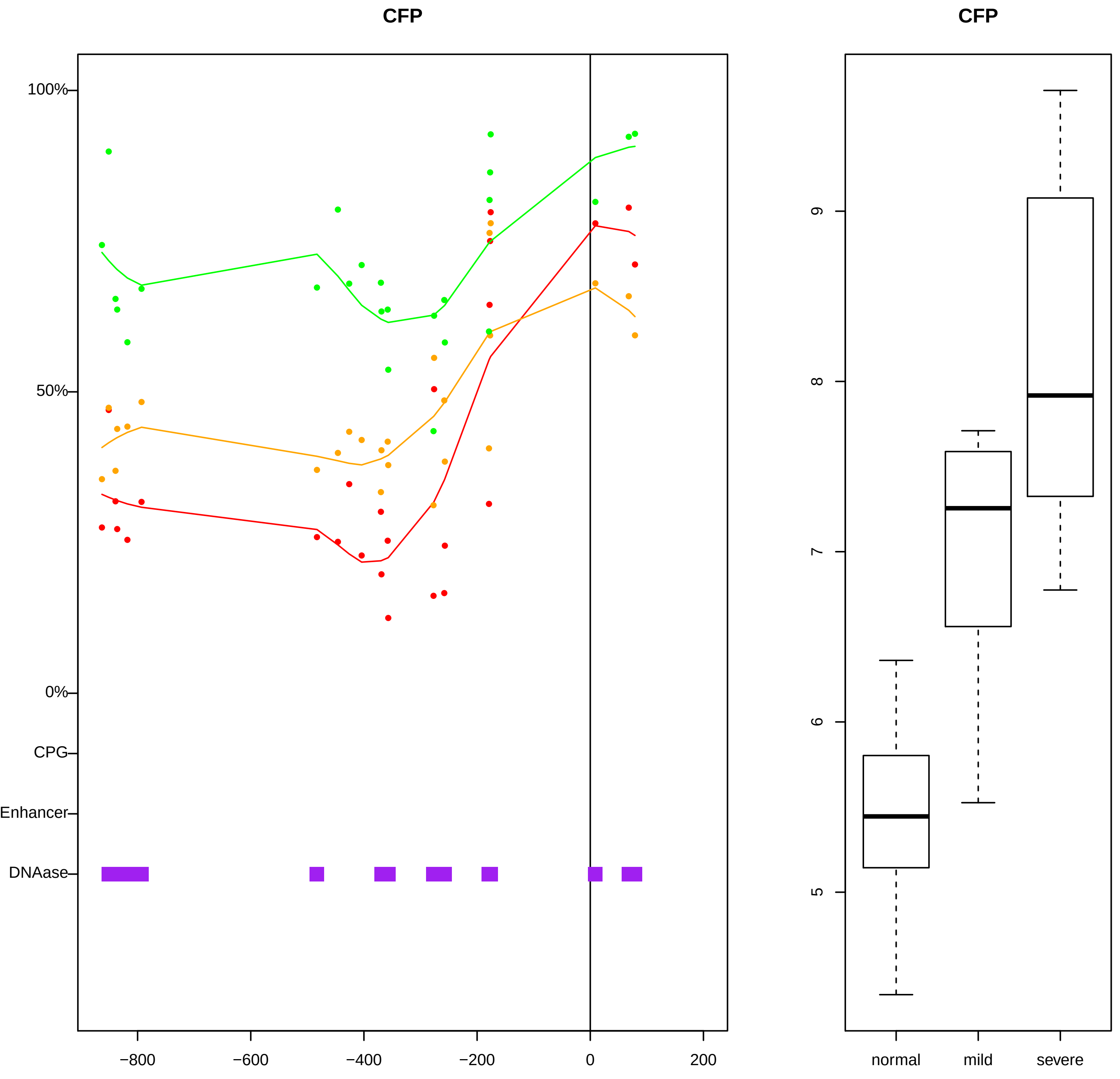
CFP
100%
50%
0%
CPG
Enhancer
DNAase
−800
−600
−400
−200
0
200
CFP
9
8
7
6
5
normal
mild
severe

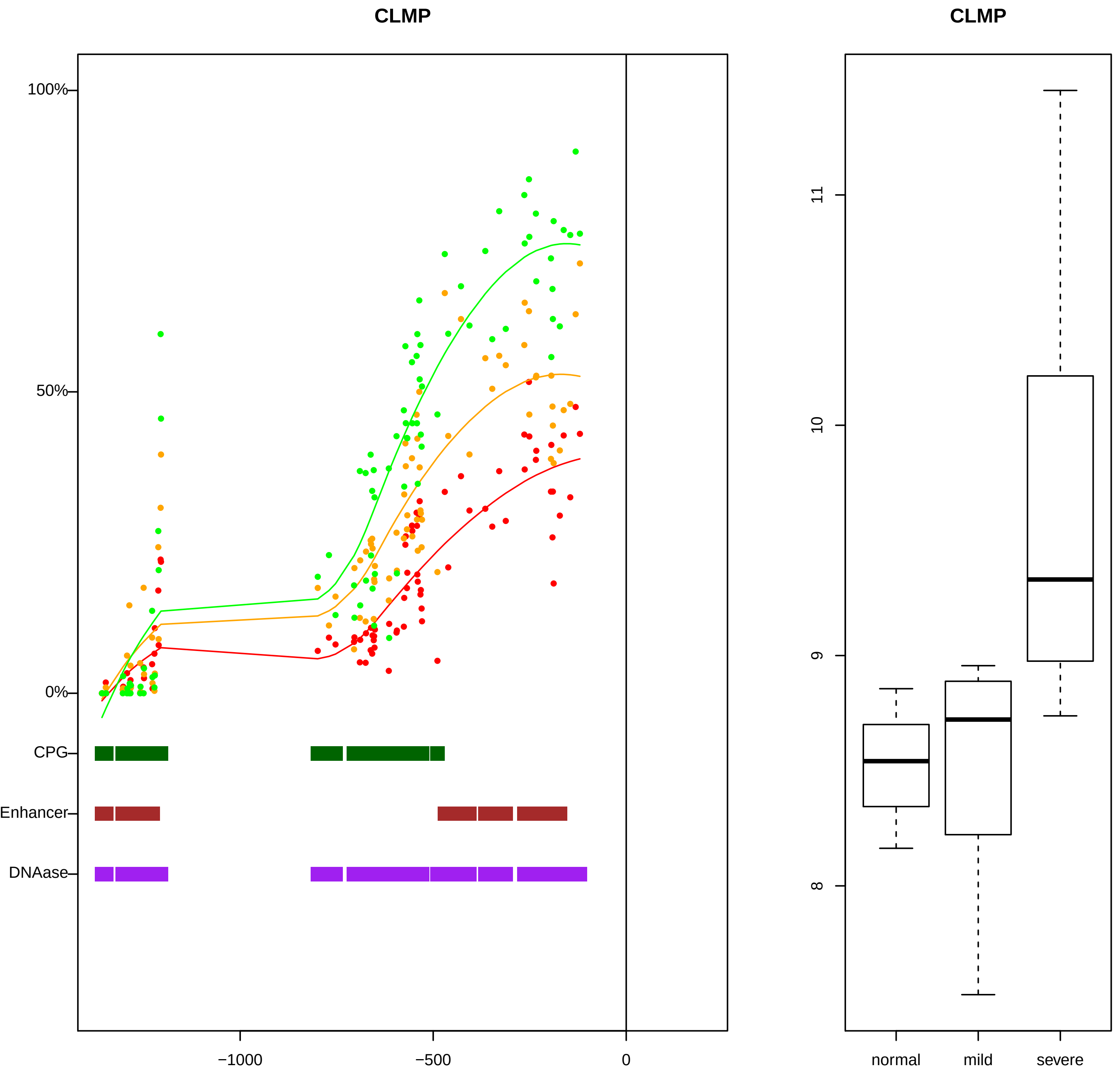
CLMP
100%
50%
0%
CPG
Enhancer
DNAase
−1000
−500
0
CLMP
11
10
9
8
normal
mild
severe

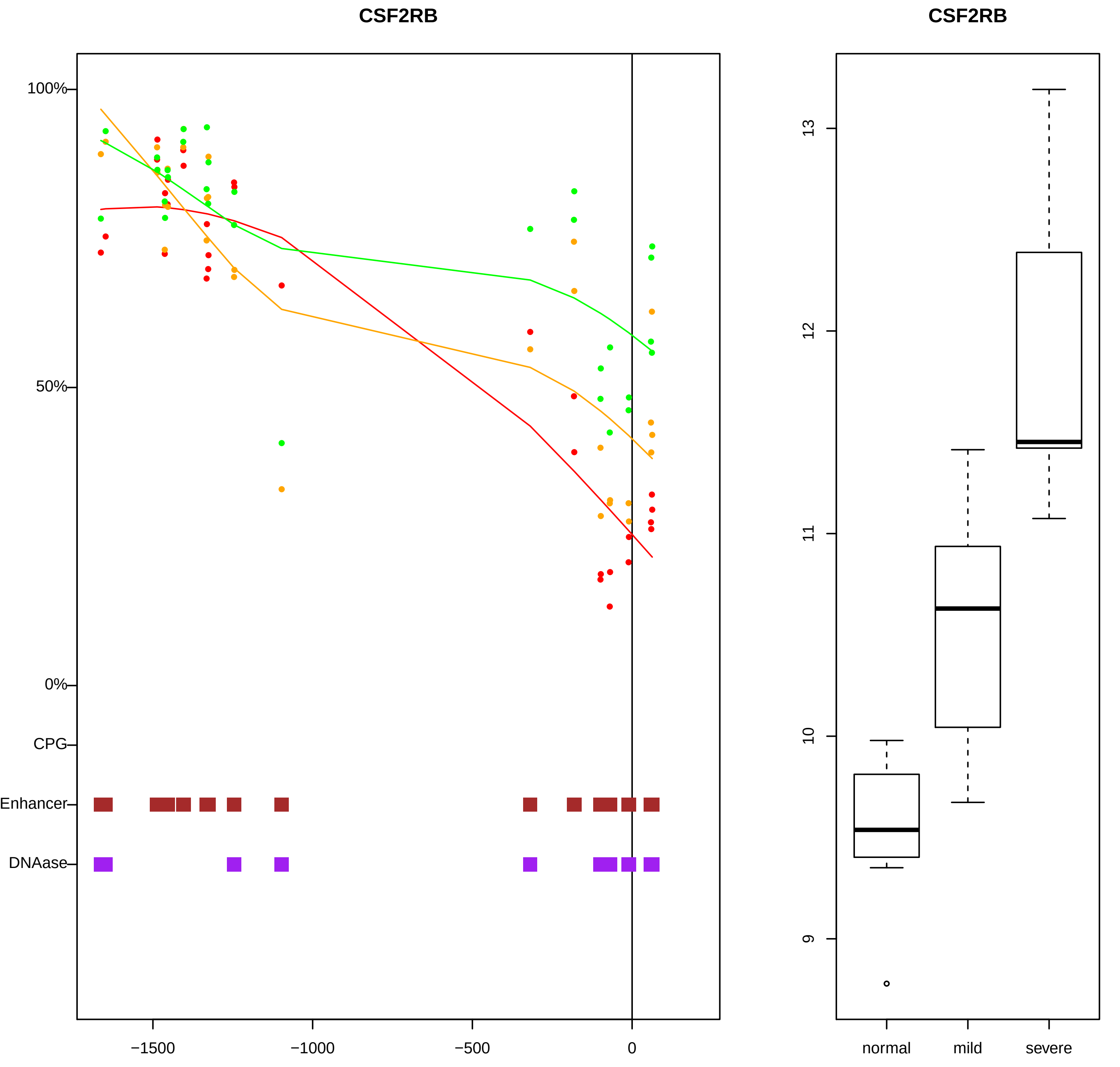

CSF2RB
100%
50%
0%
CPG
Enhancer
DNAase
−1500
−1000
−500
0
CSF2RB
13
12
11
10
9
normal
mild
severe

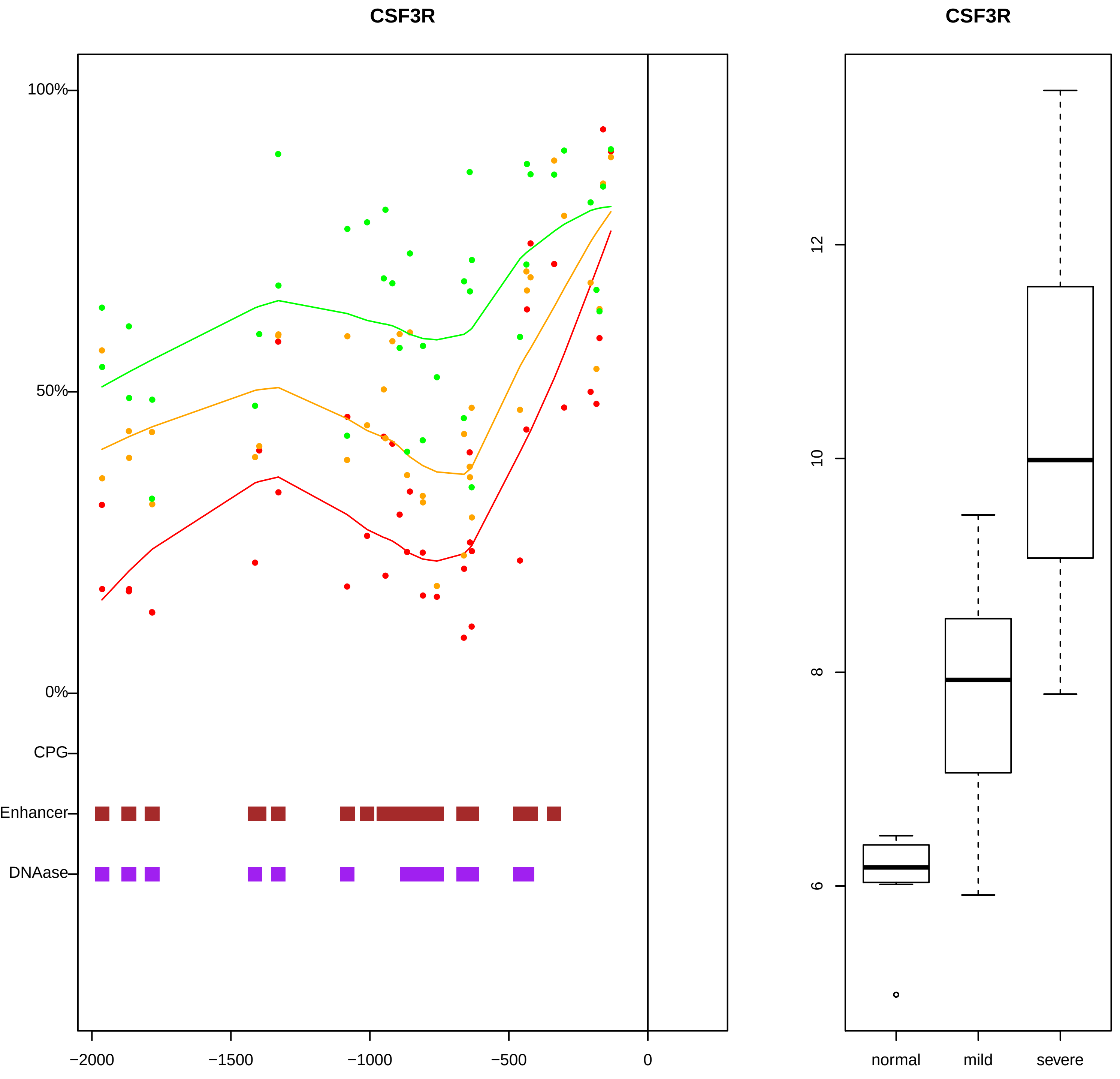

CSF3R
100%
50%
0%
CPG
Enhancer
DNAase
−2000
−1500
−1000
−500
0
CSF3R
6
8
10
12
normal
mild
severe

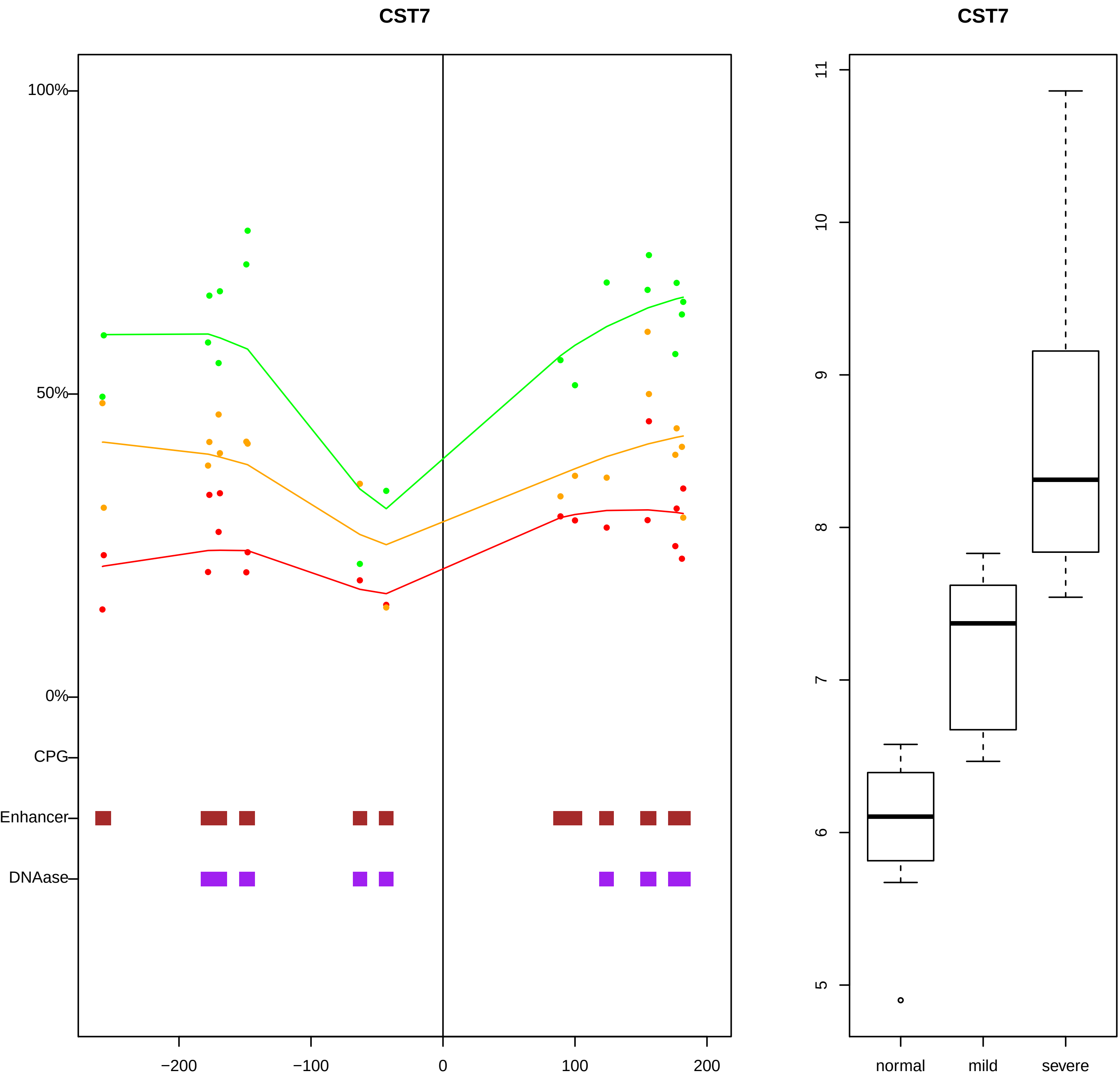

CST7
100%
50%
0%
CPG
Enhancer
DNAase
−200
−100
0
100
200
CST7
11
10
9
8
7
6
5
normal
mild
severe

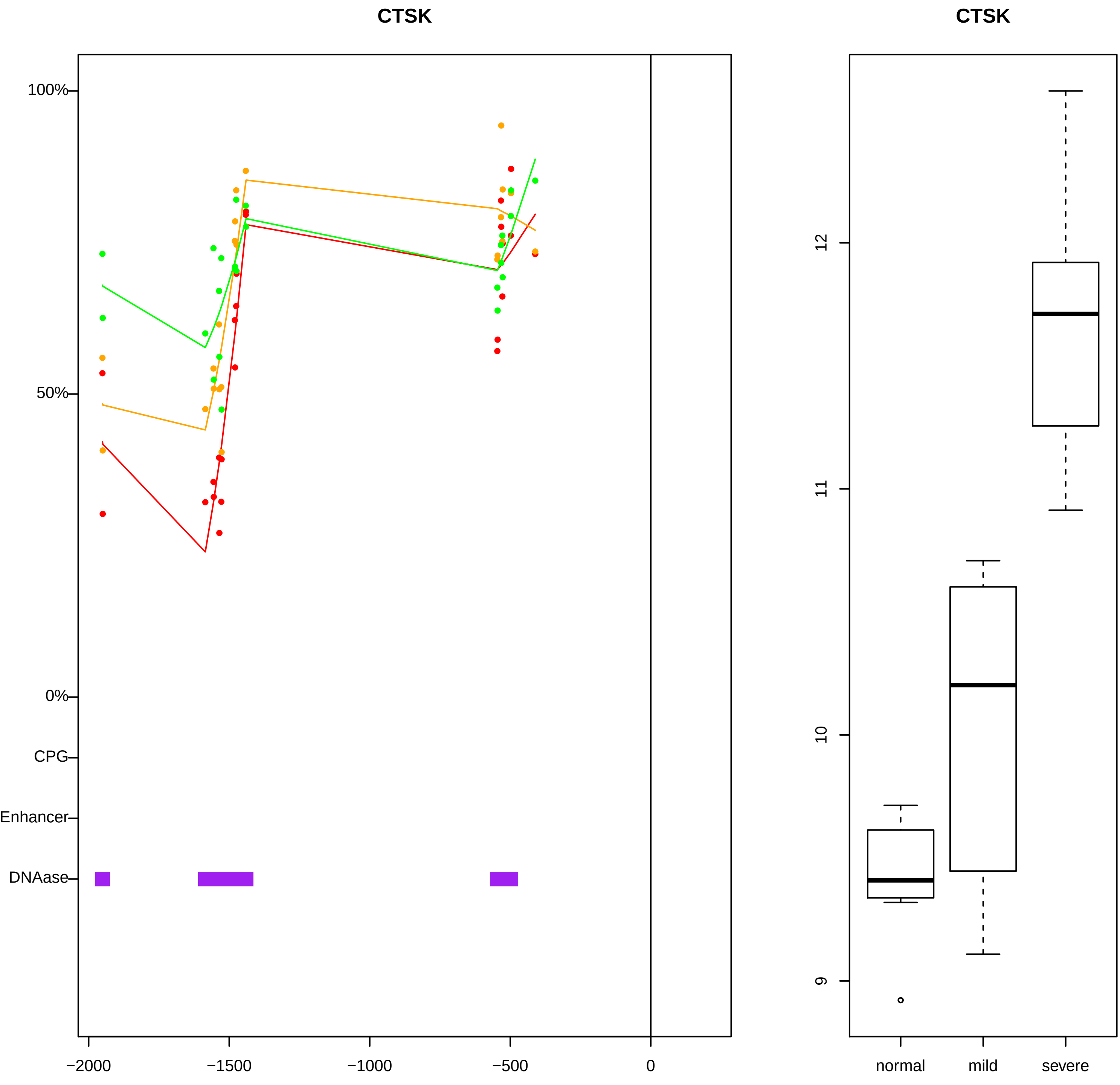

CTSK
100%
50%
0%
CPG
Enhancer
DNAase
−2000
−1500
−1000
−500
0
CTSK
12
11
10
9
normal
mild
severe

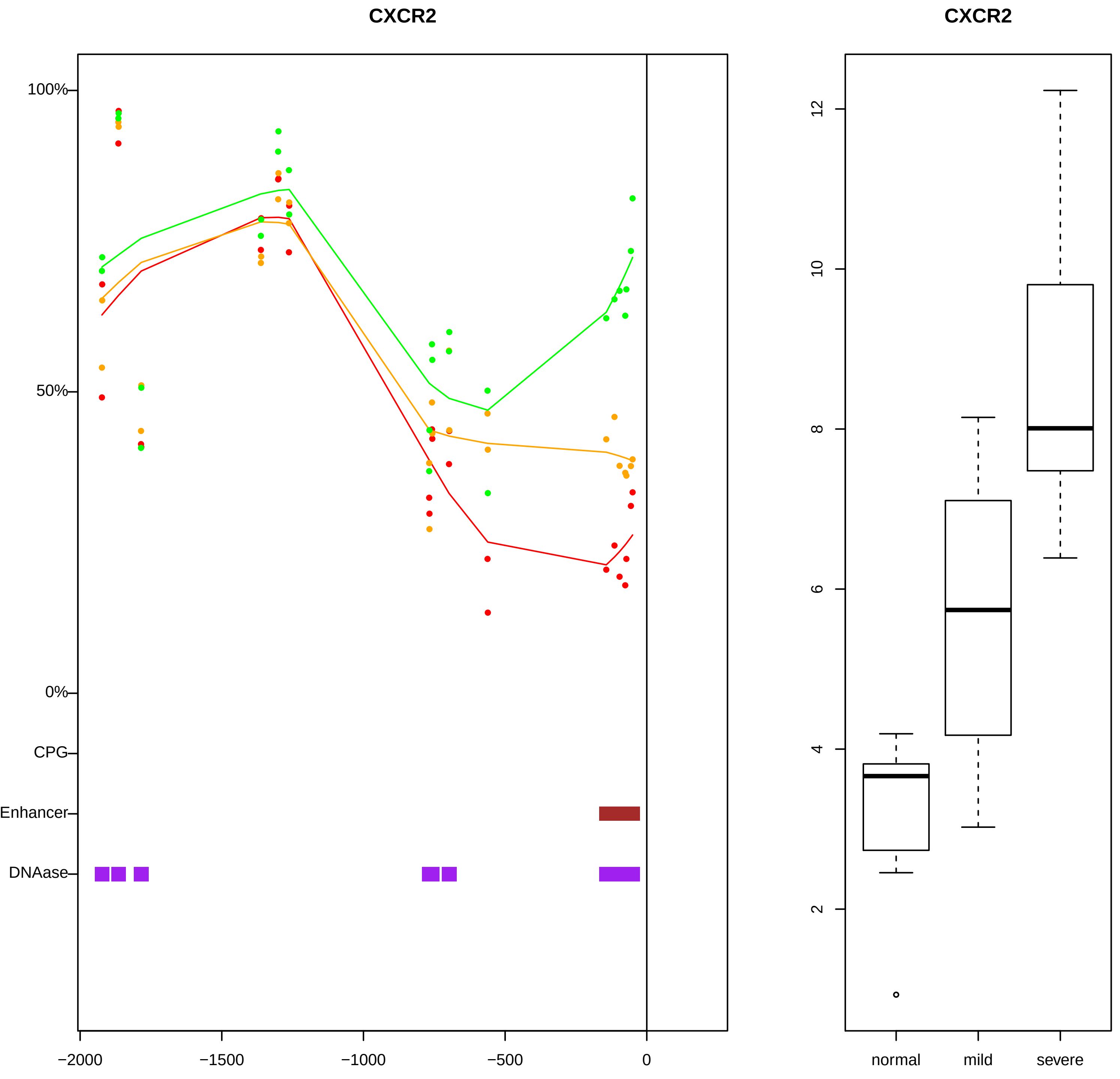
CXCR2
100%
50%
0%
CPG
Enhancer
DNAase
−2000
−1500
−1000
−500
0
CXCR2
12
10
8
6
4
2
normal
mild
severe

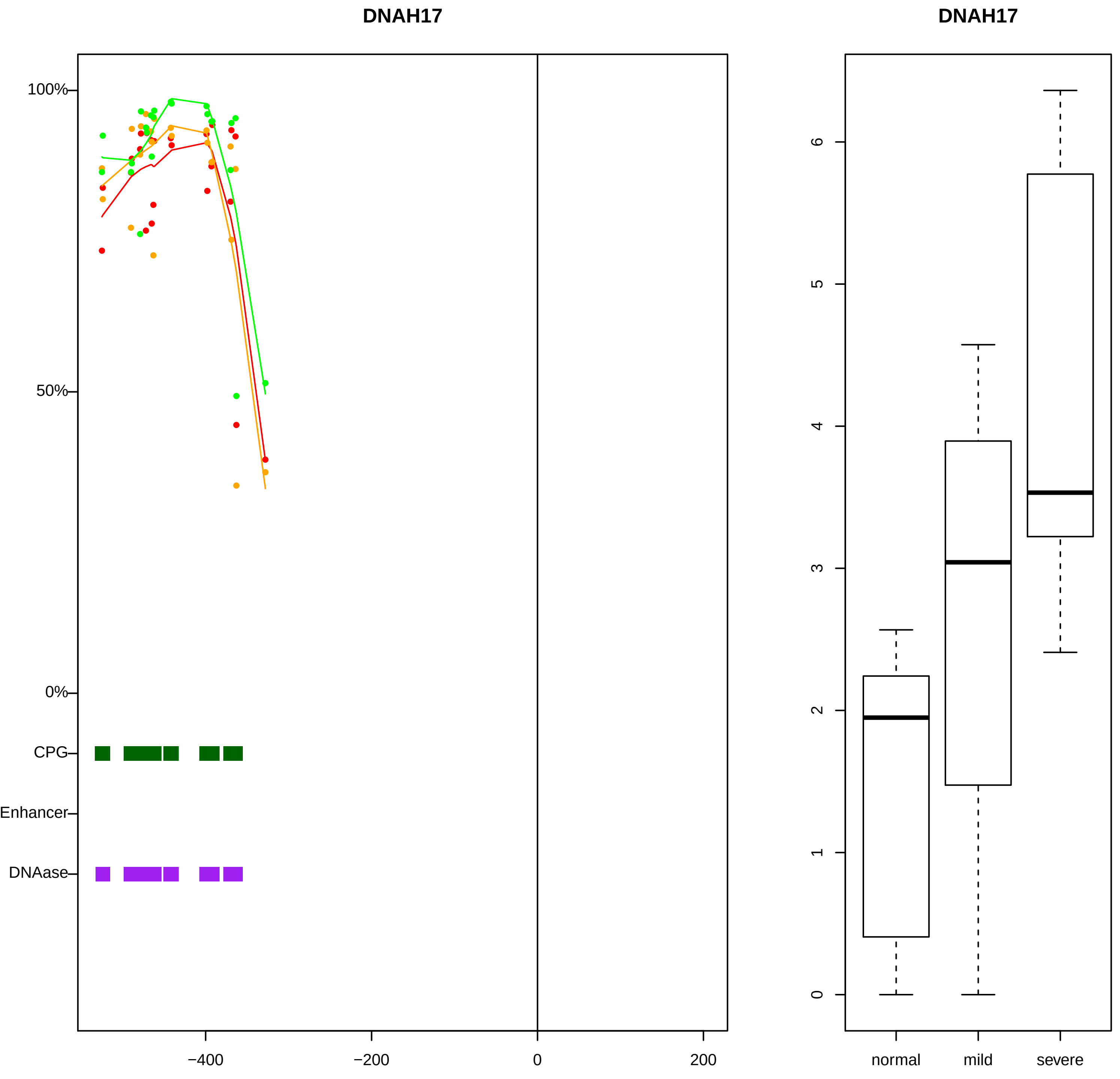
DNAH17
100%
50%
0%
CPG
Enhancer
DNAase
−400
−200
0
200
DNAH17
0
1
2
3
4
5
6
normal
mild
severe

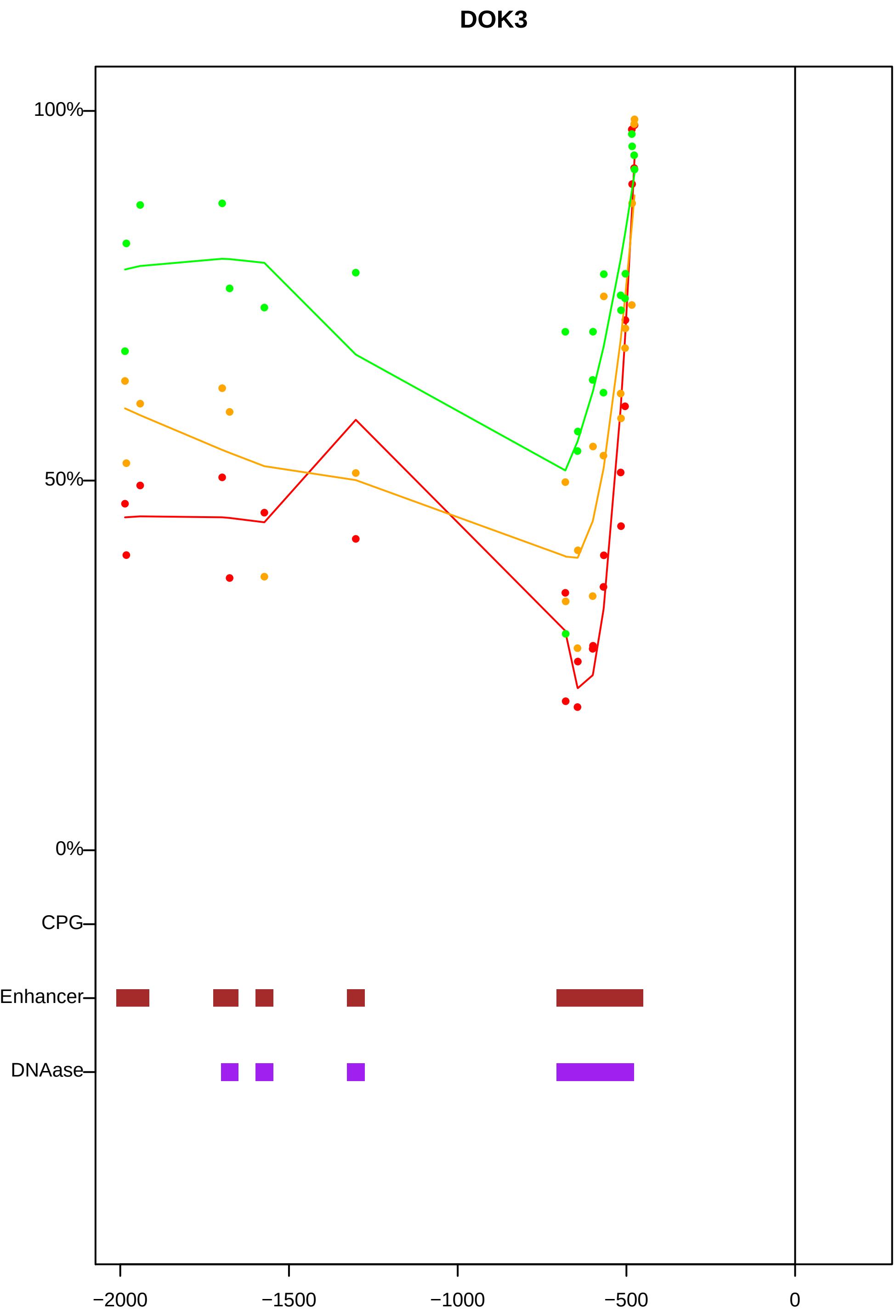

DOK3
100%
50%
0%
CPG
Enhancer
DNAase
−2000
−1500
−1000
−500
0

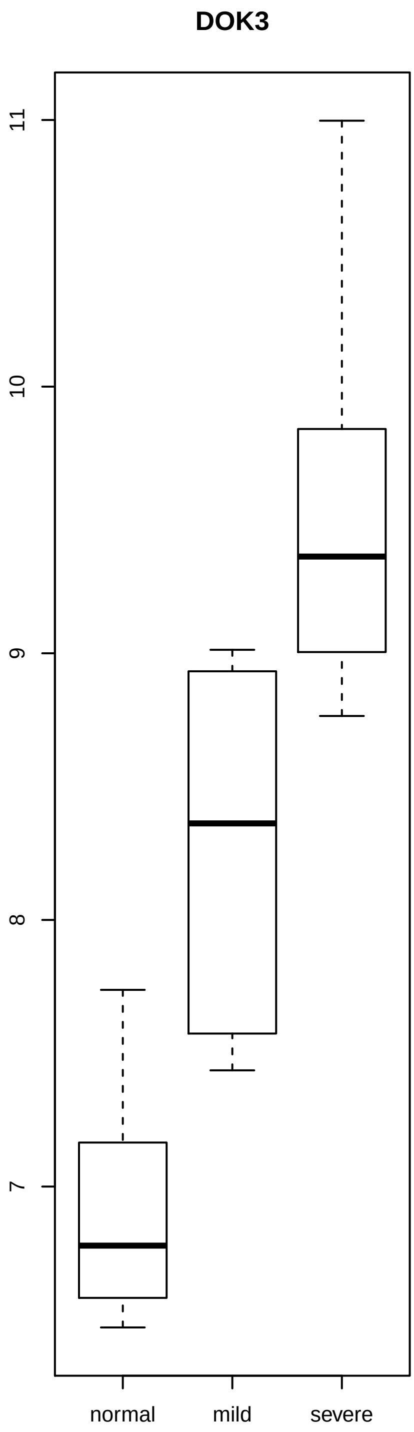

DOK3
11
10
9
8
7
normal
mild
severe

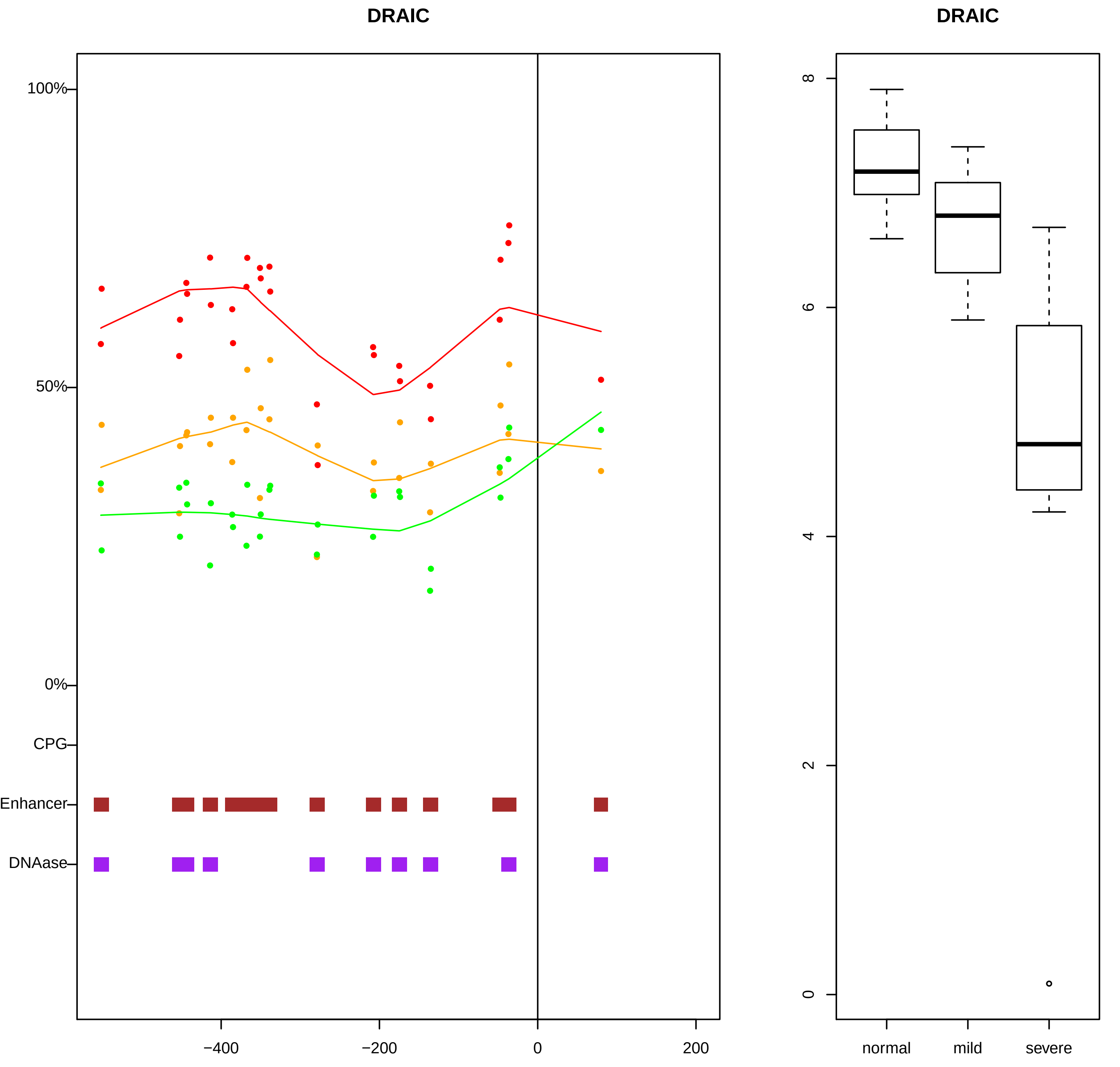
DRAIC
100%
50%
0%
CPG
Enhancer
DNAase
−400
−200
0
200
DRAIC
0
2
4
6
8
normal
mild
severe

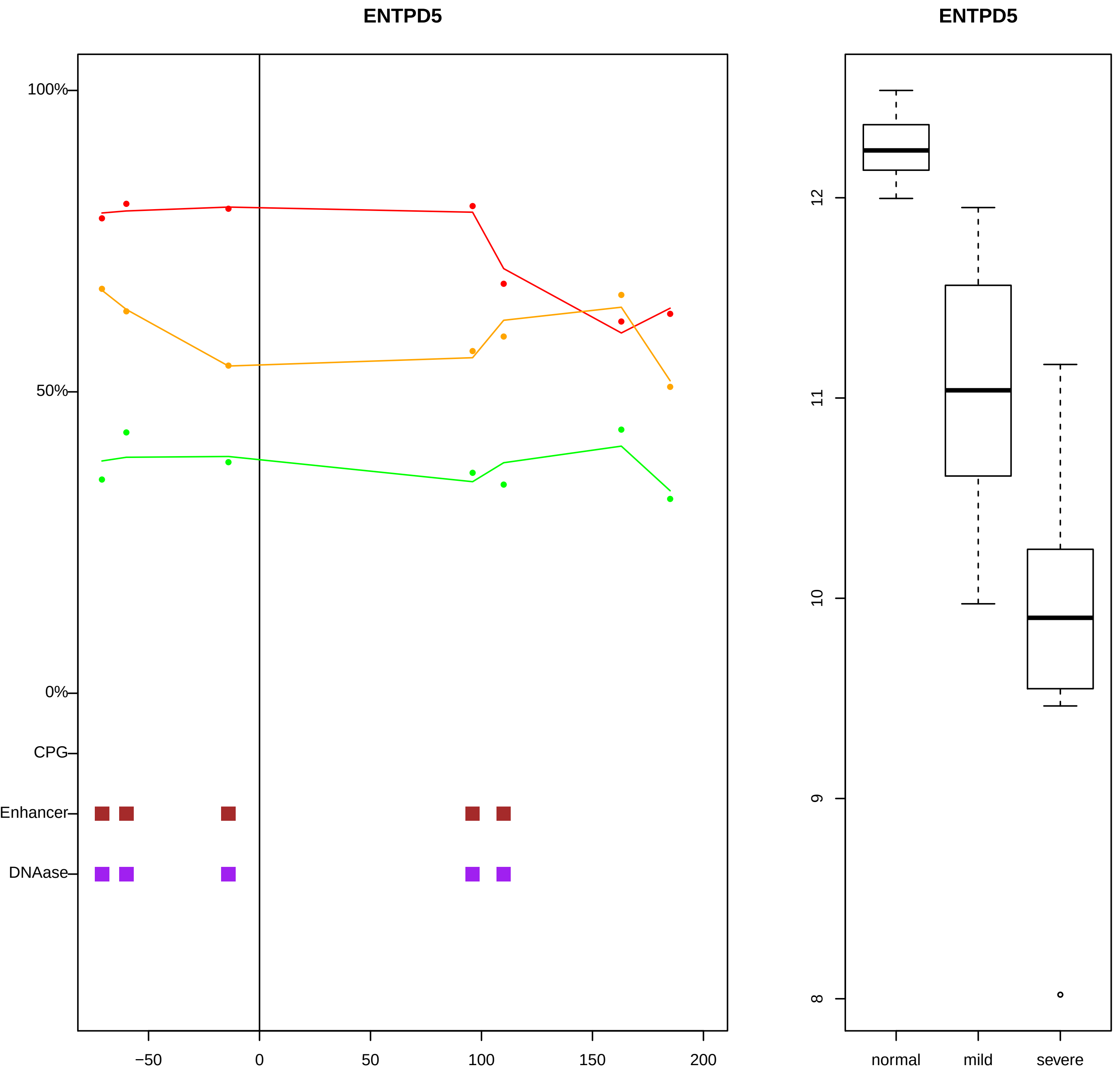

ENTPD5
100%
50%
0%
CPG
Enhancer
DNAase
−50
0
50
100
150
200
ENTPD5
12
11
10
9
8
normal
mild
severe

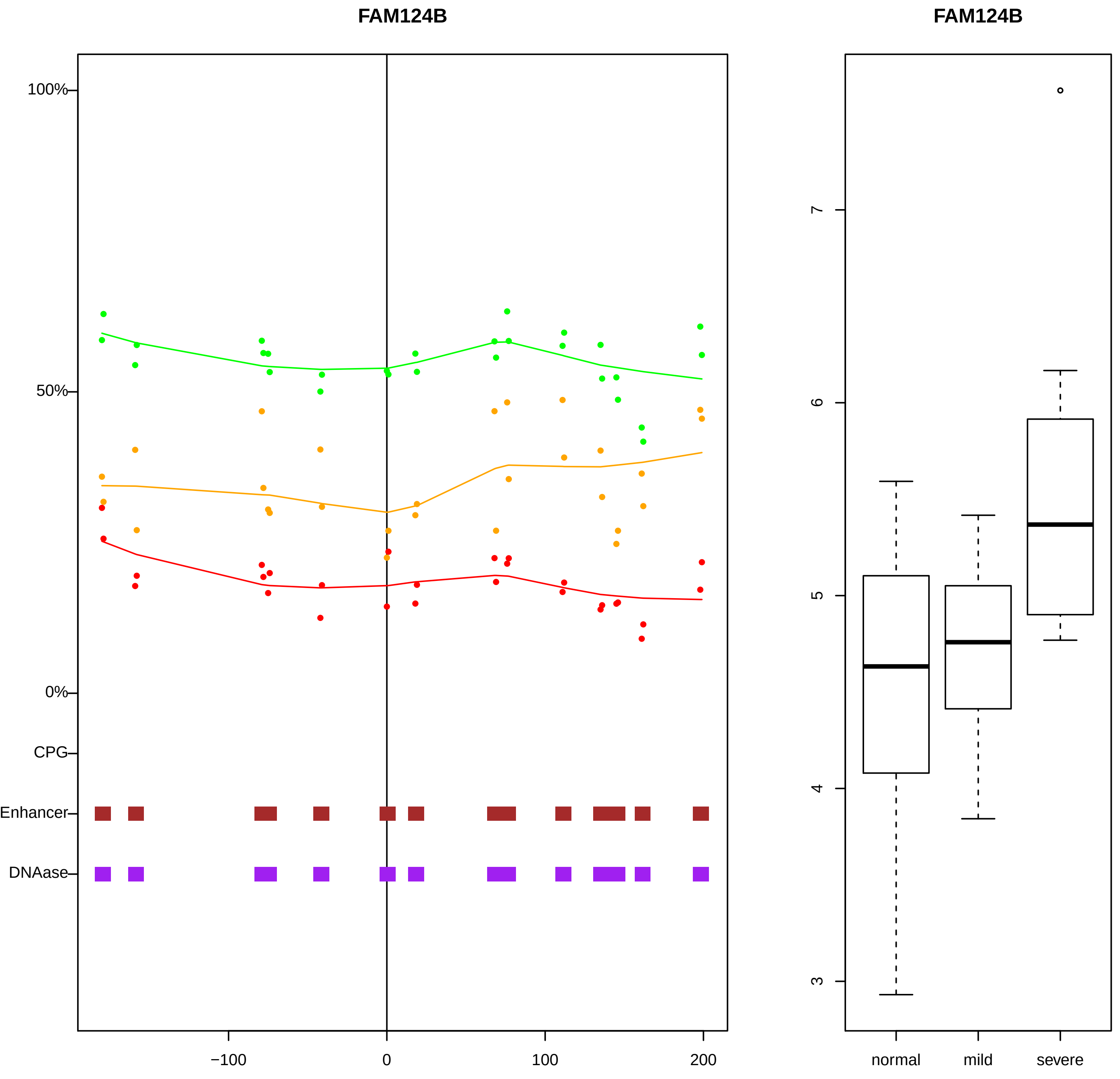
FAM124B
100%
50%
0%
CPG
Enhancer
DNAase
−100
0
100
200
FAM124B
7
6
5
4
3
normal
mild
severe

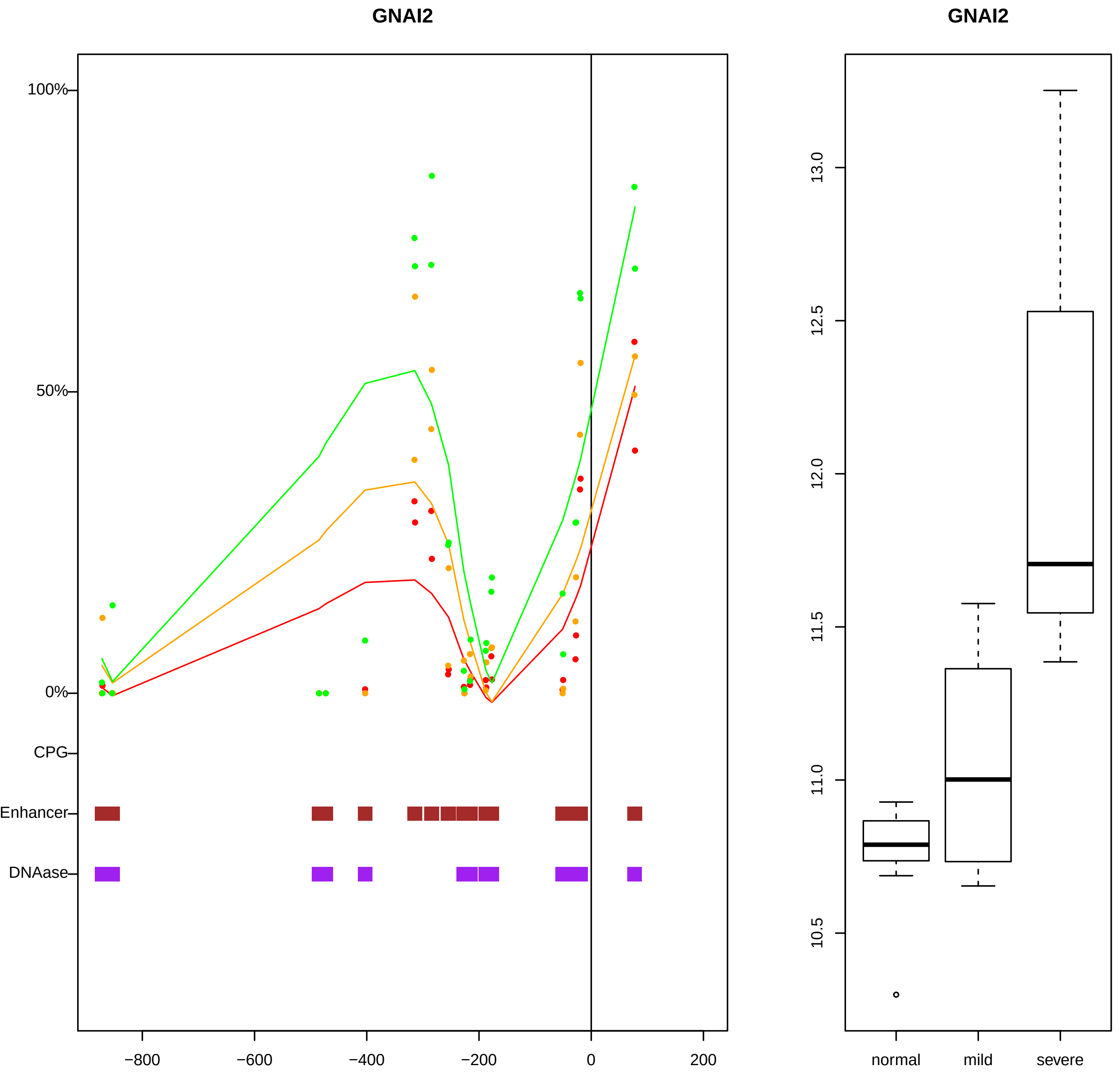
GNAI2
100%
50%
0%
CPG
Enhancer
DNAase
−800
−600
−400
−200
0
200
GNAI2
13.0
12.5
12.0
11.5
11.0
10.5
normal
mild
severe

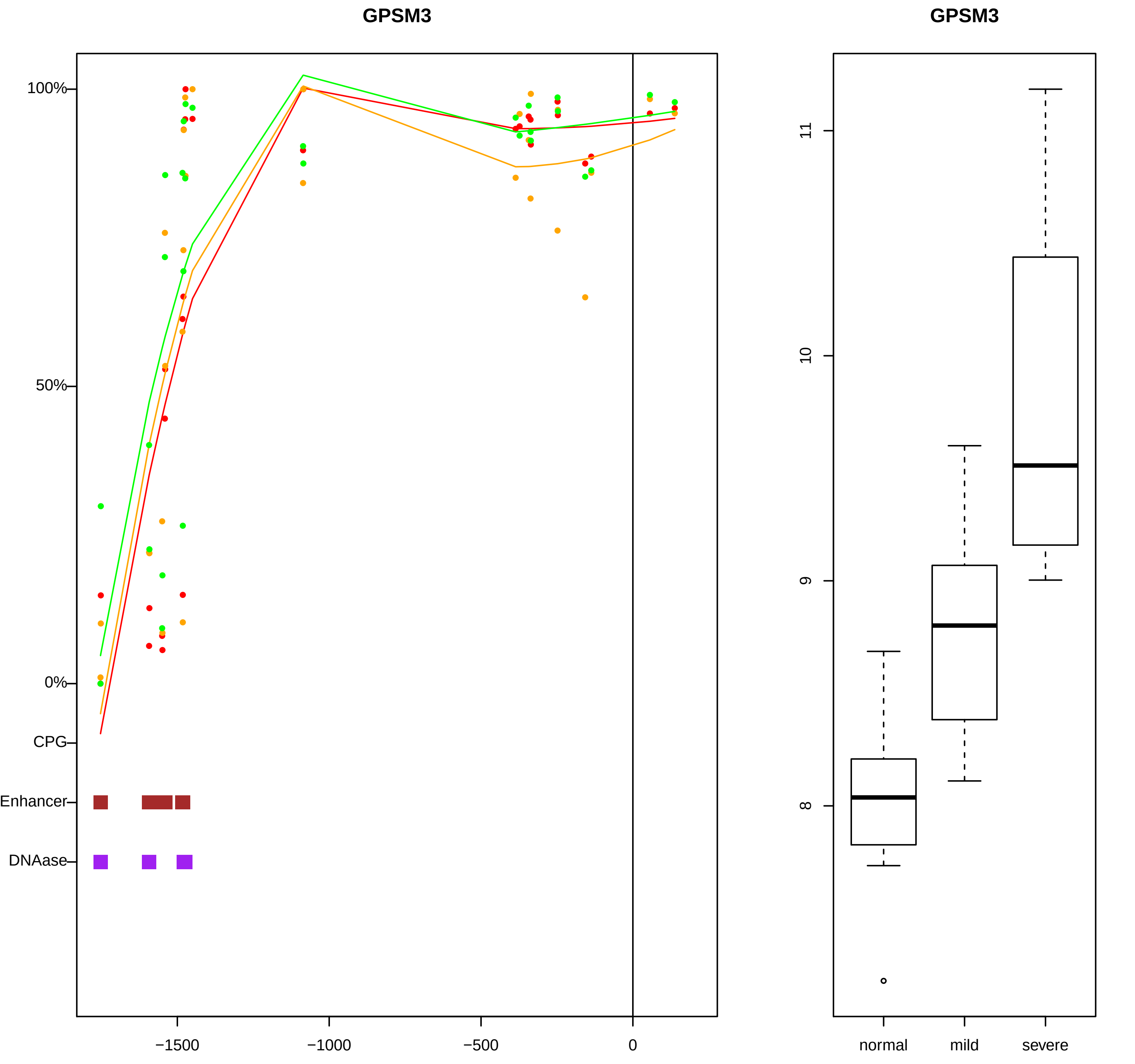

GPSM3
100%
50%
0%
CPG
Enhancer
DNAase
−1500
−1000
−500
0
GPSM3
11
10
9
8
normal
mild
severe

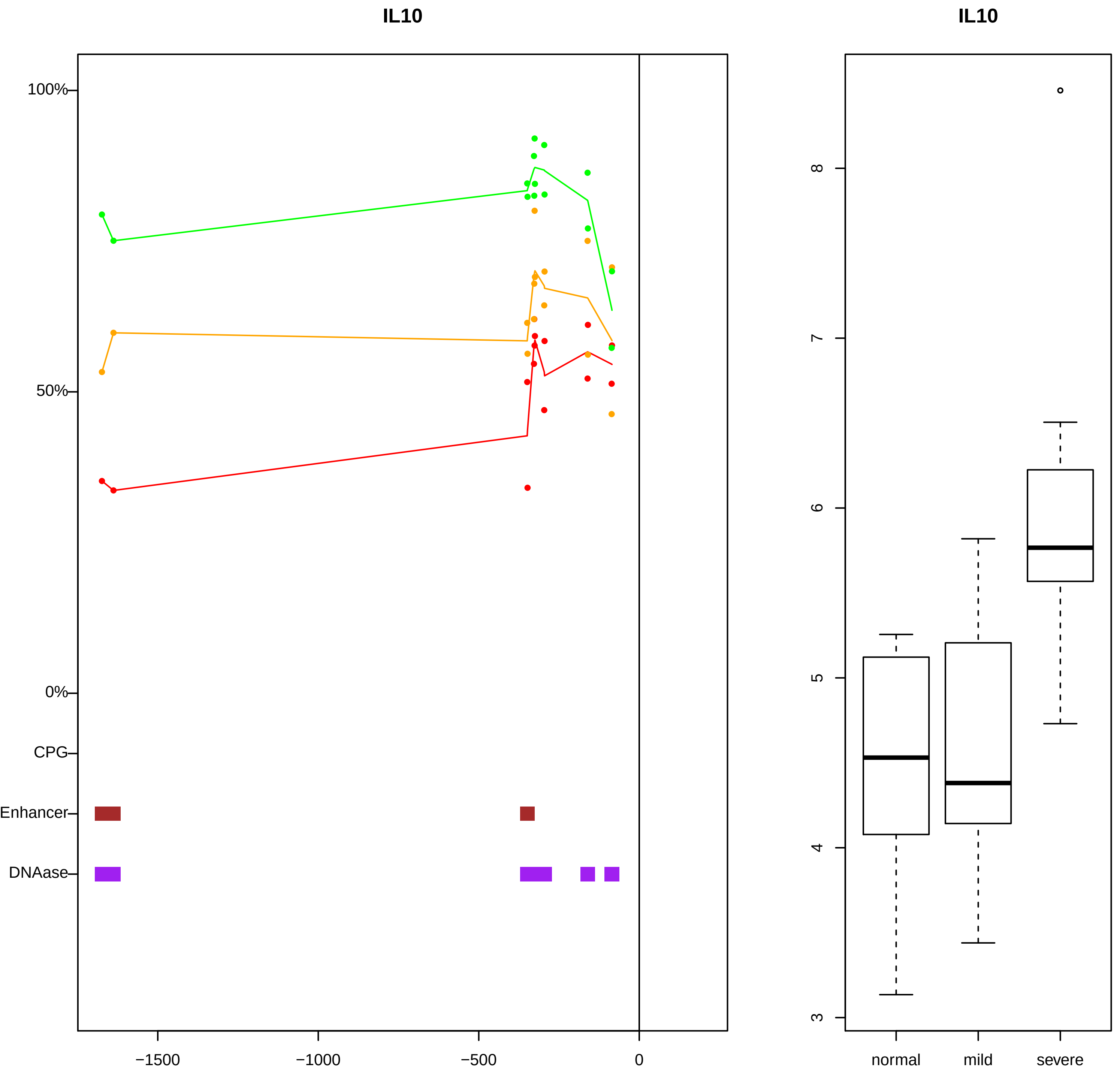
IL10
100%
50%
0%
CPG
Enhancer
DNAase
−1500
−1000
−500
0
IL10
8
7
6
5
4
3
normal
mild
severe

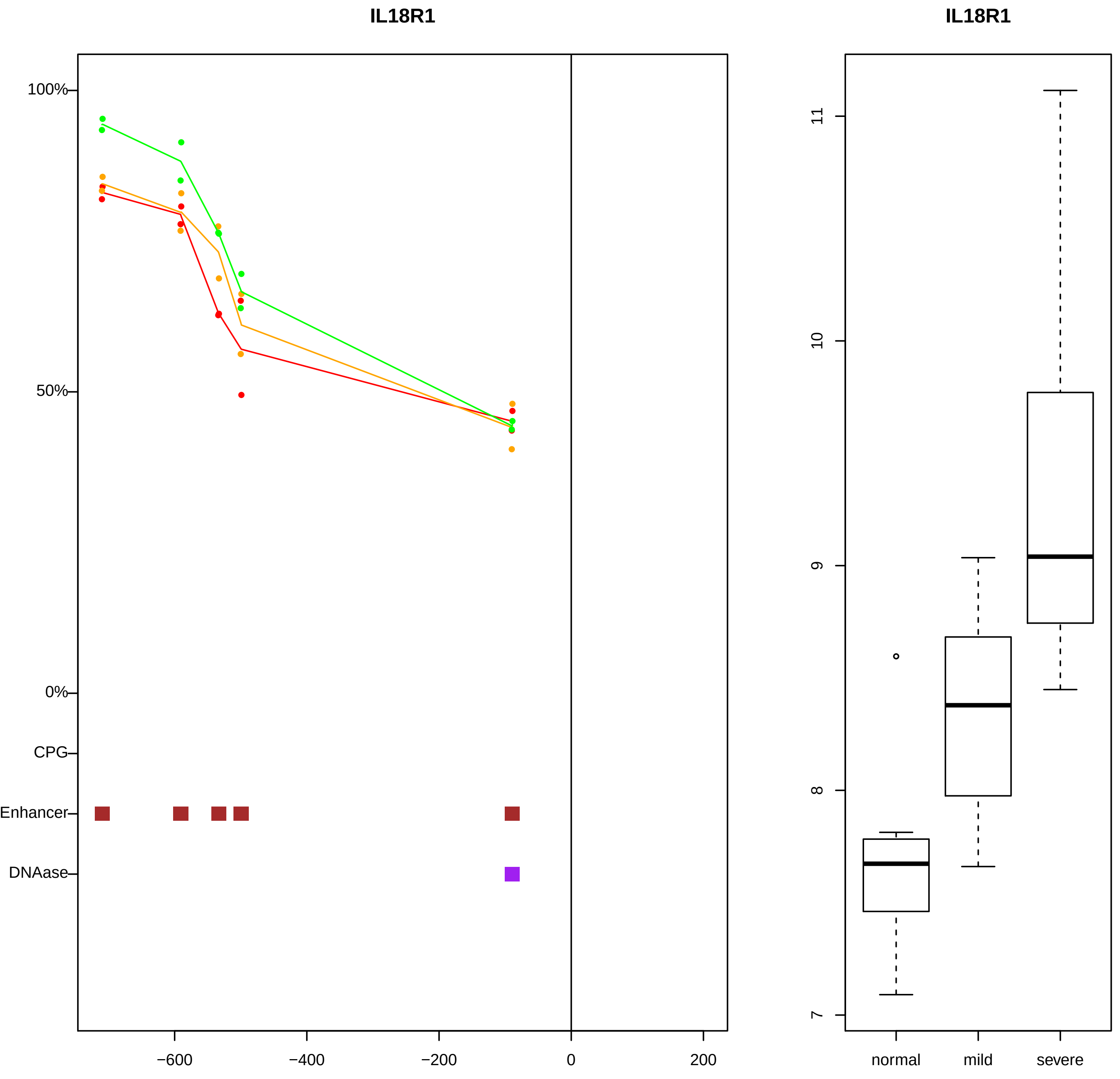

IL18R1
100%
50%
0%
CPG
Enhancer
DNAase
−600
−400
−200
0
200
IL18R1
11
10
9
8
7
normal
mild
severe

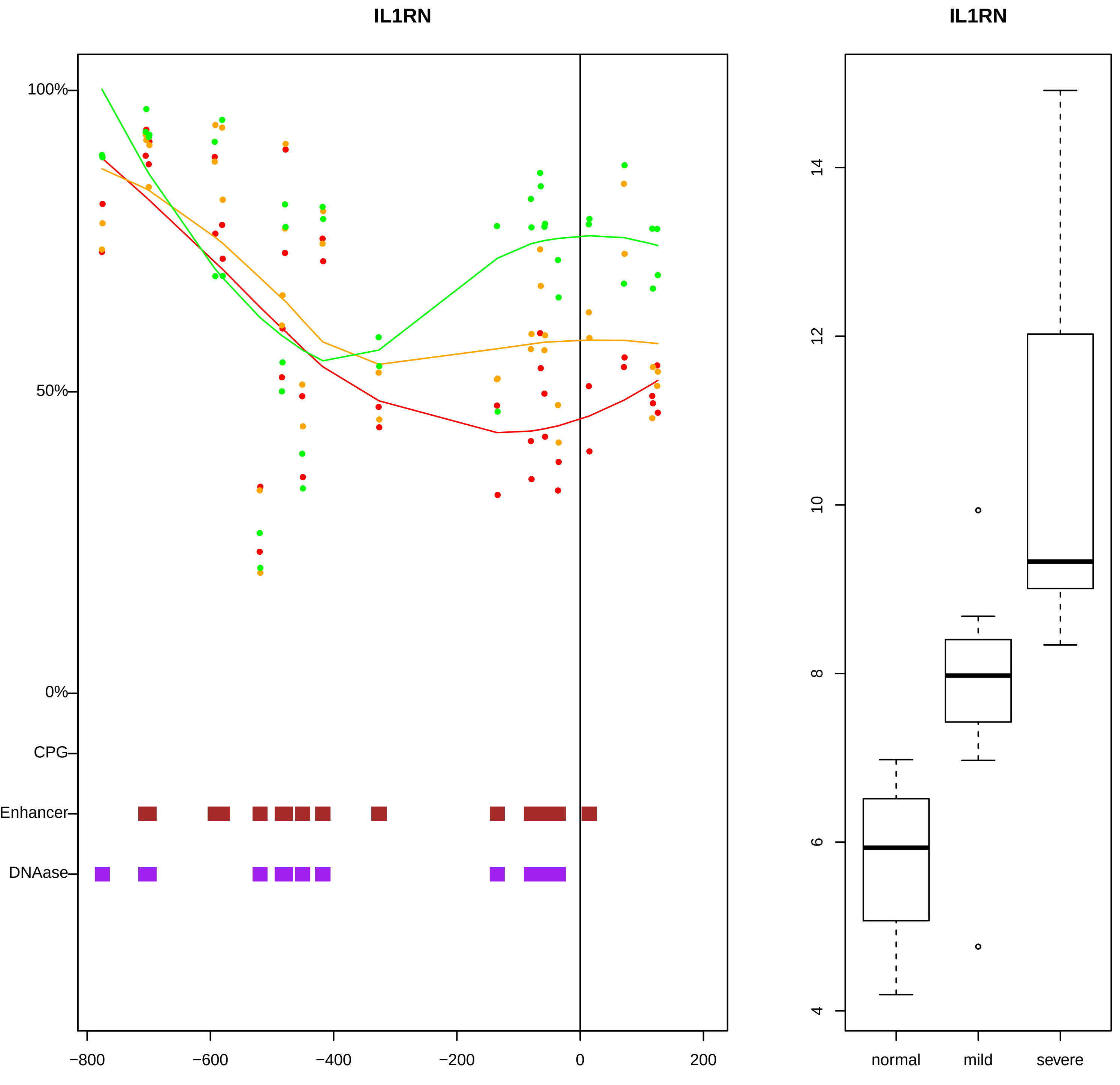
IL1RN
100%
50%
0%
CPG
Enhancer
DNAase
−800
−600
−400
−200
0
200
IL1RN
4
6
8
10
12
14
normal
mild
severe

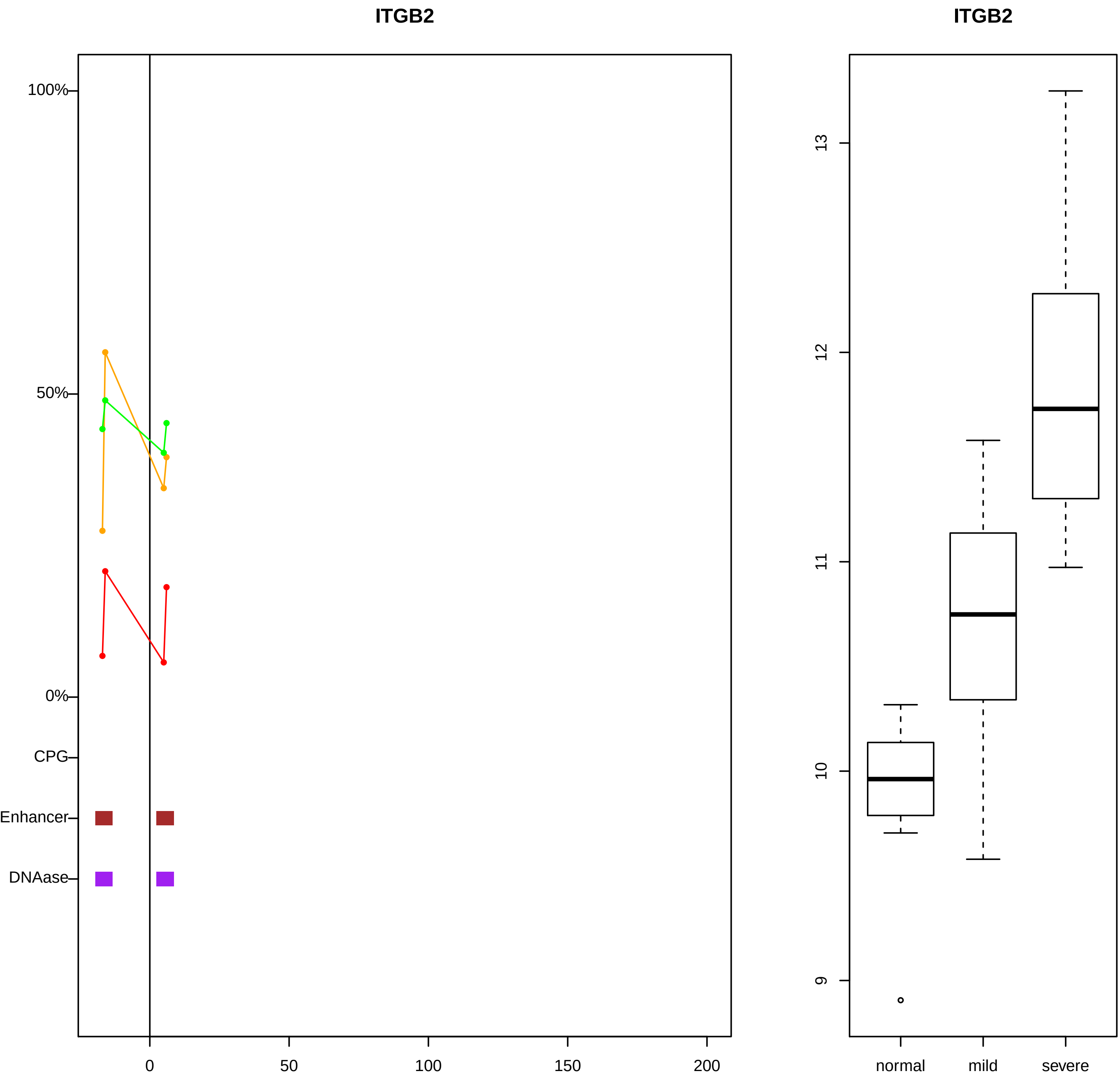
ITGB2
100%
50%
0%
CPG
Enhancer
DNAase
0
50
100
150
200
ITGB2
13
12
11
10
9
normal
mild
severe

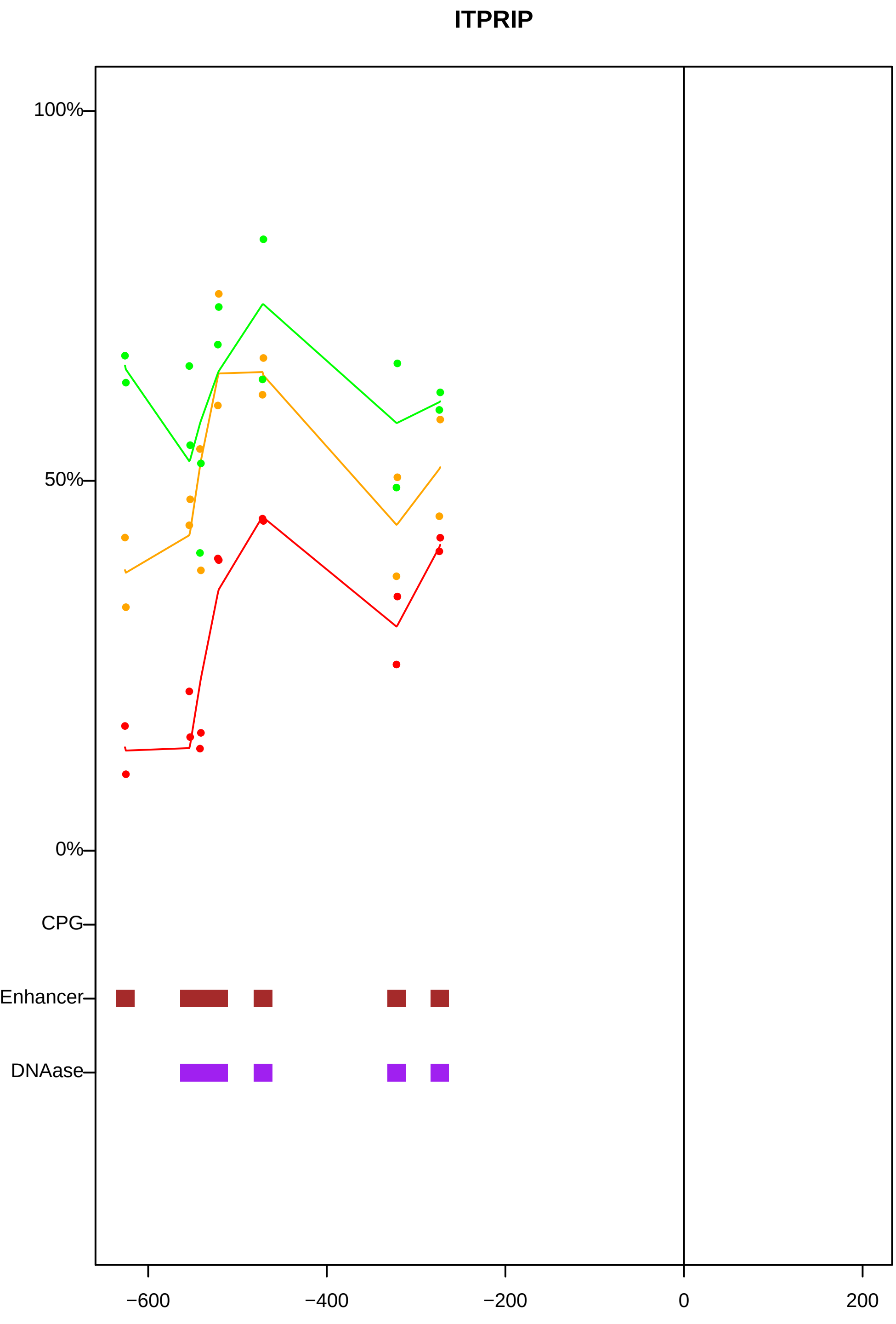
ITPRIP
100%
50%
0%
CPG
Enhancer
DNAase
−600
−400
−200
0
200

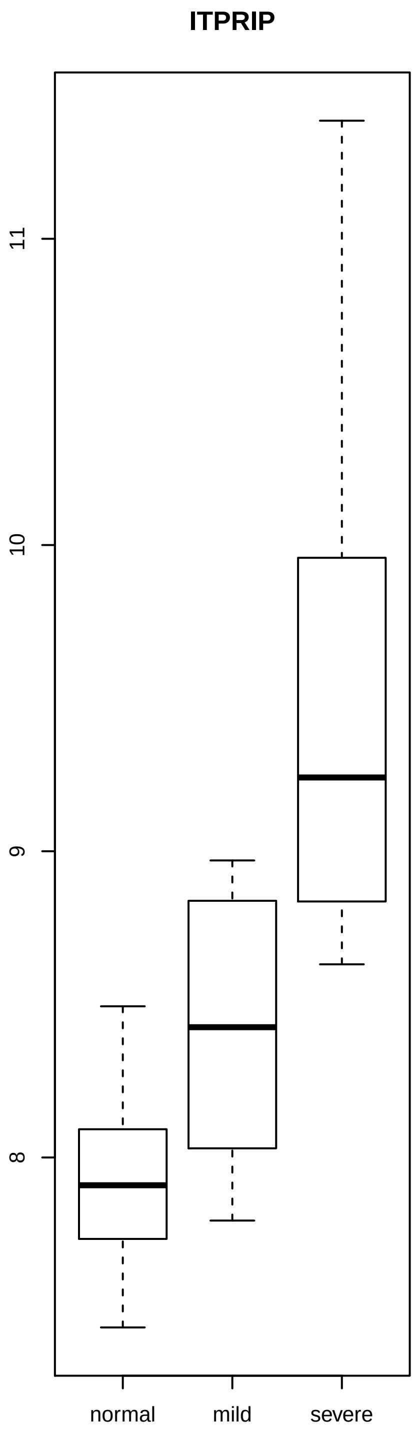
ITPRIP
11
10
9
8
normal
mild
severe

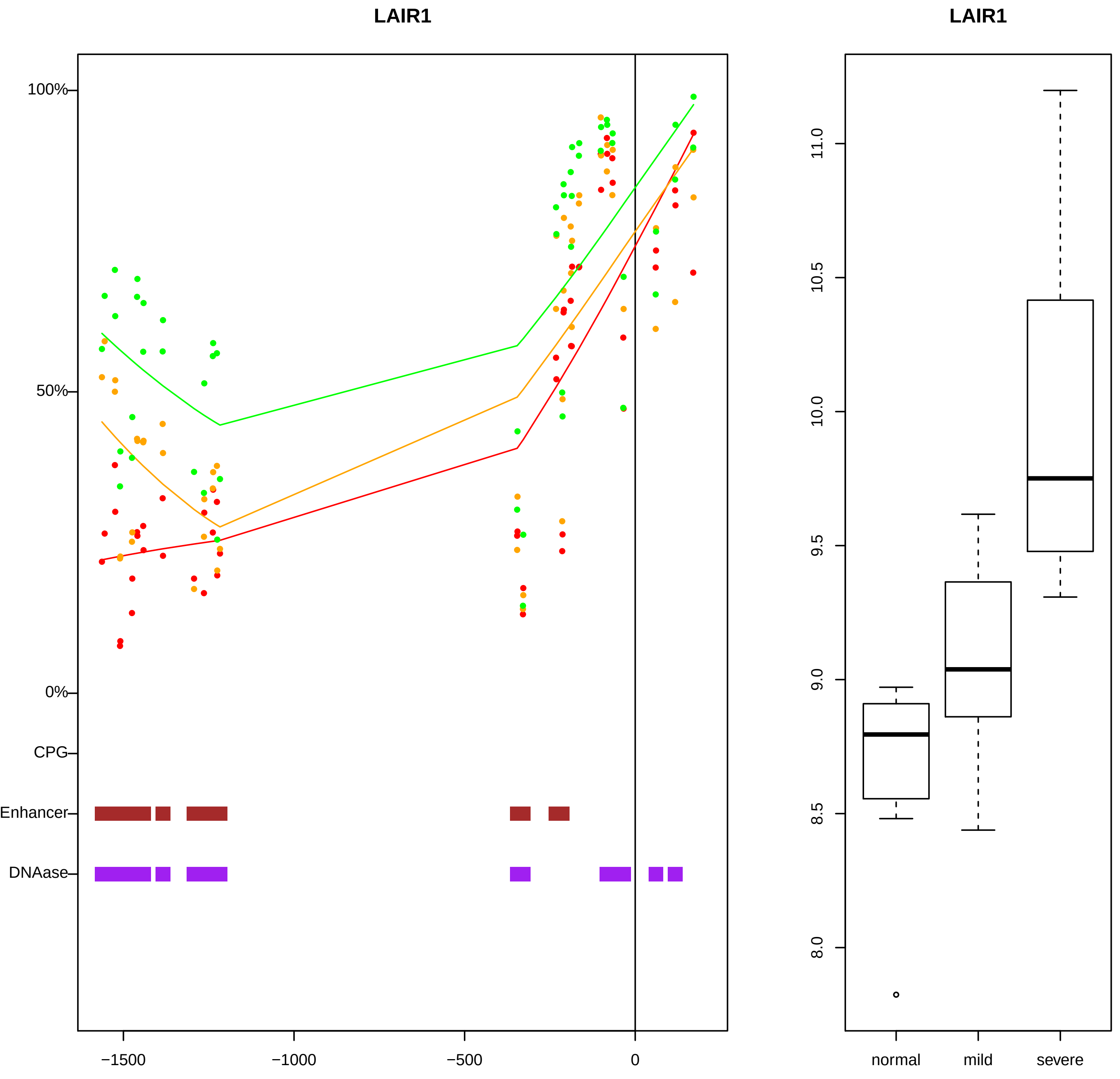
LAIR1
100%
50%
0%
CPG
Enhancer
DNAase
−1500
−1000
−500
0
LAIR1
11.0
10.5
10.0
9.5
9.0
8.5
8.0
normal
mild
severe

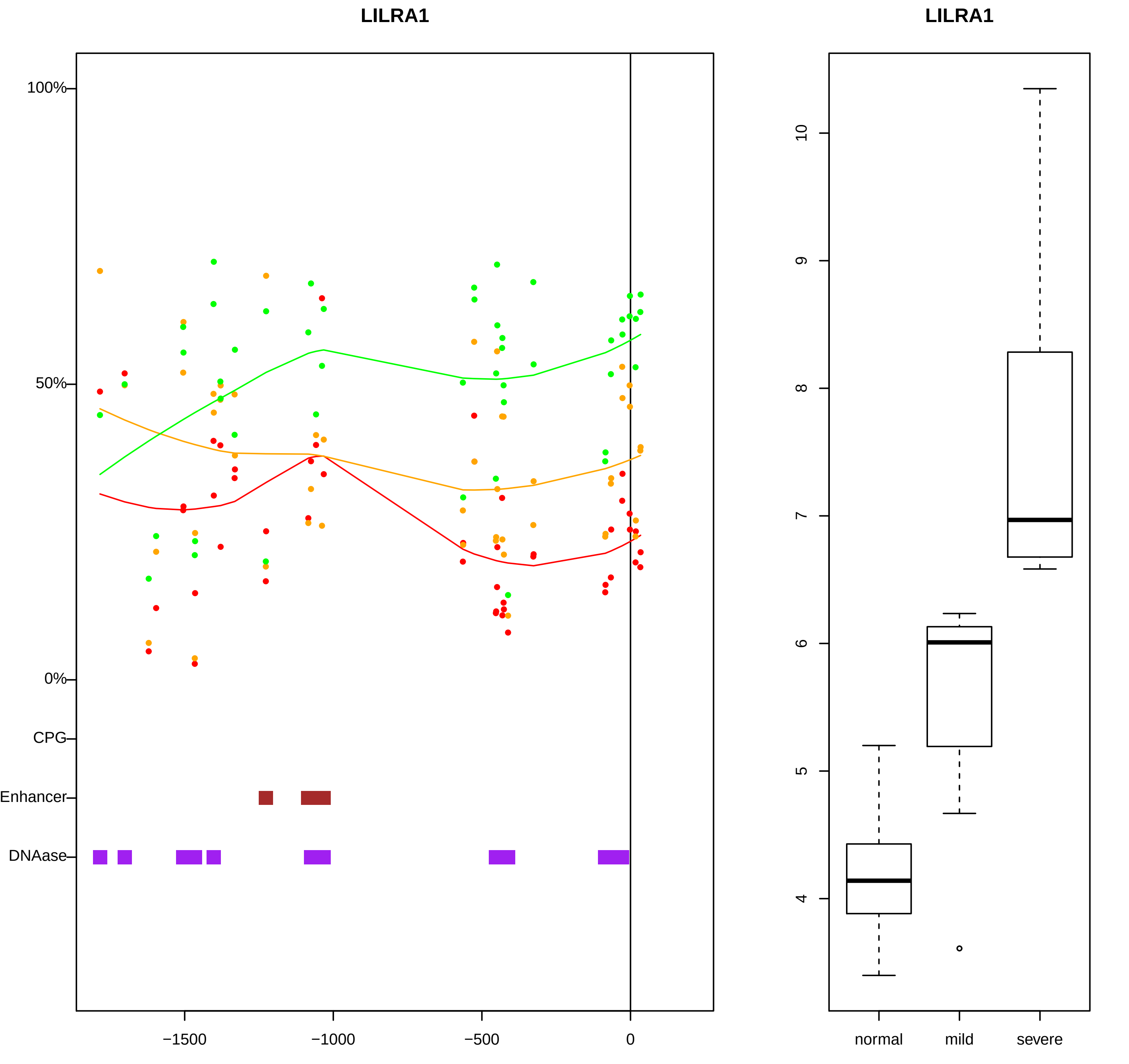
LILRA1
100%
50%
0%
CPG
Enhancer
DNAase
−1500
−1000
−500
0
LILRA1
10
9
8
7
6
5
4
normal
mild
severe

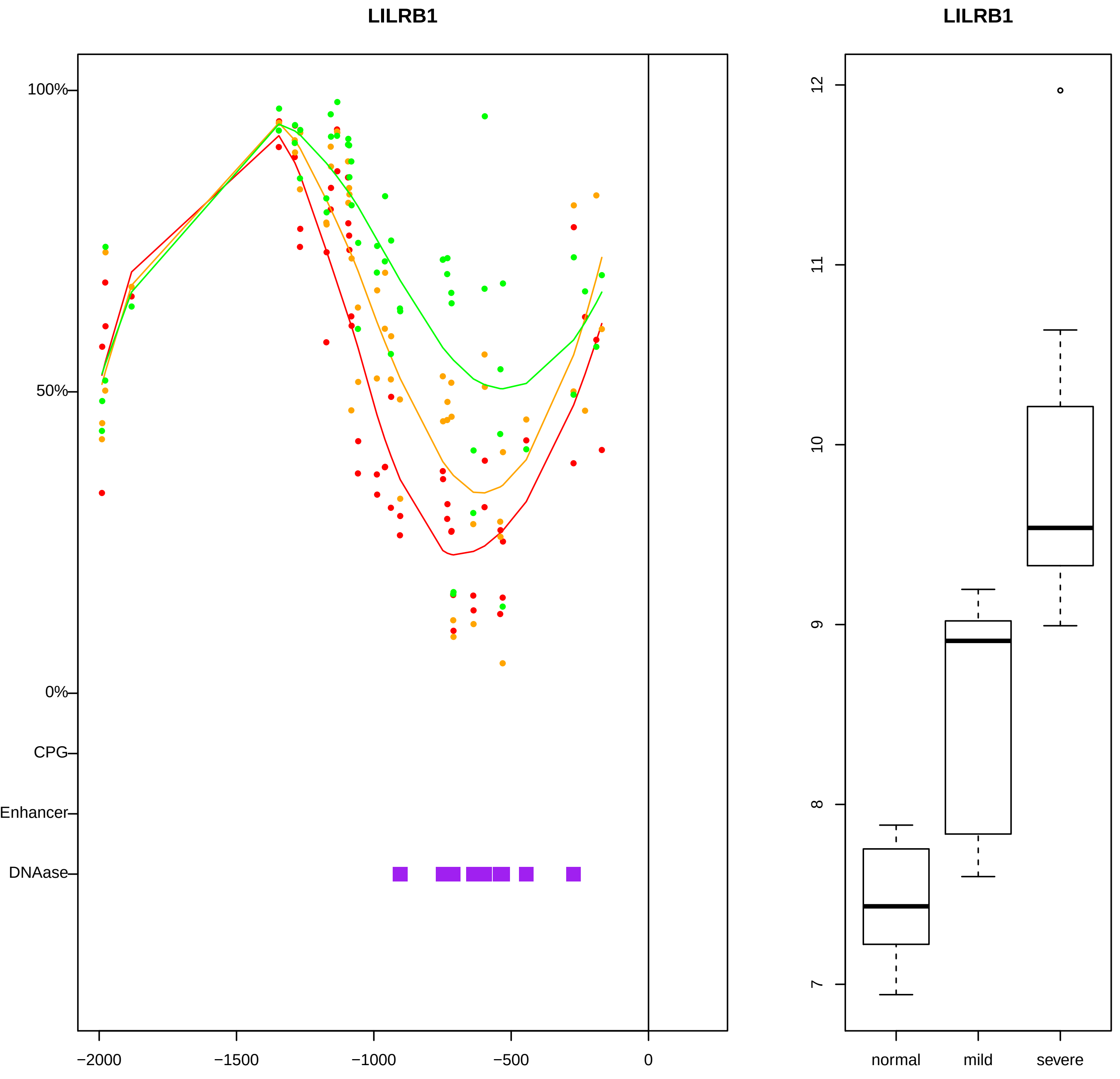
LILRB1
100%
50%
0%
CPG
Enhancer
DNAase
−2000
−1500
−1000
−500
0
LILRB1
12
11
10
9
8
7
normal
mild
severe

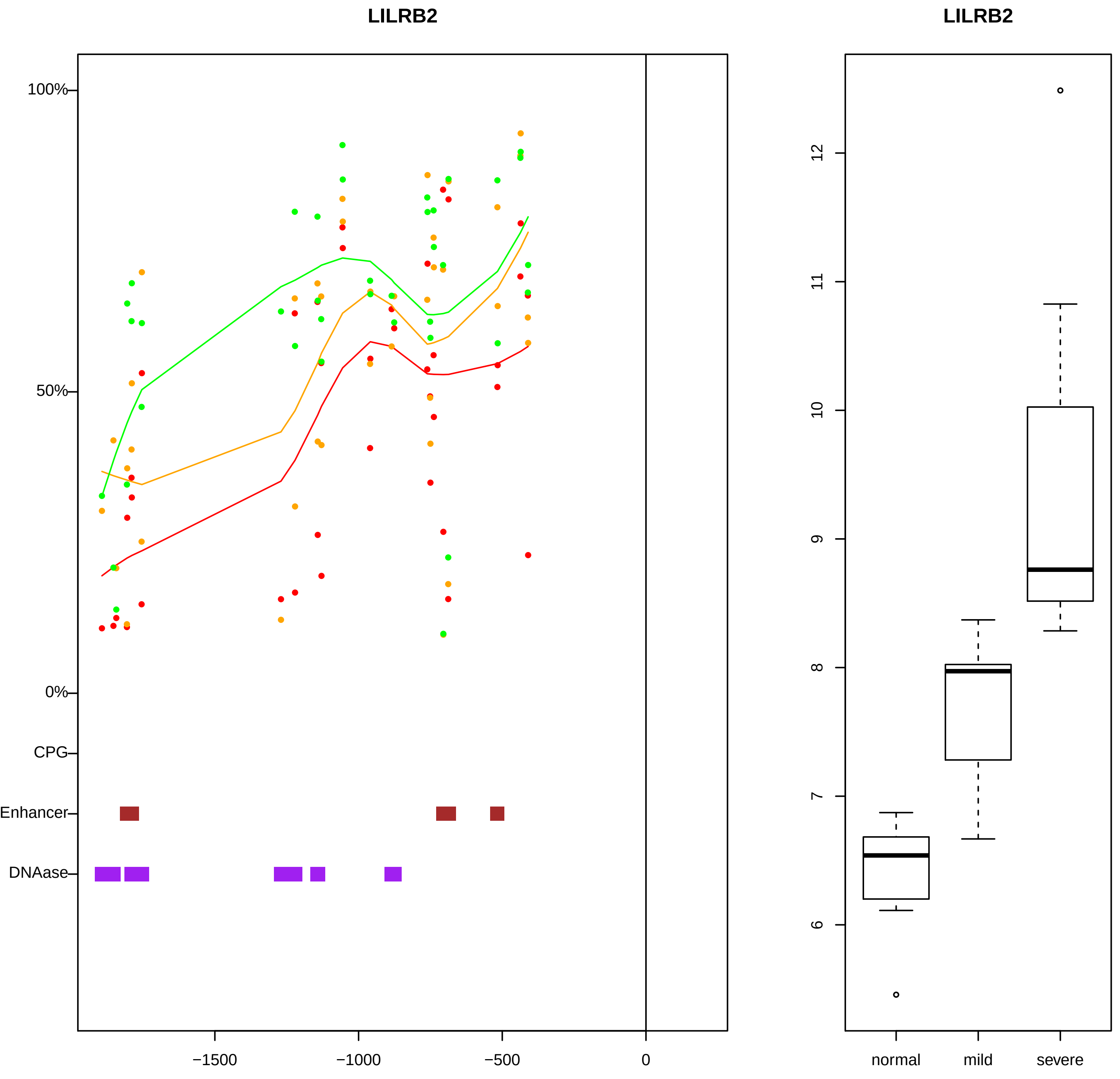
LILRB2
100%
50%
0%
CPG
Enhancer
DNAase
−1500
−1000
−500
0
LILRB2
12
11
10
9
8
7
6
normal
mild
severe

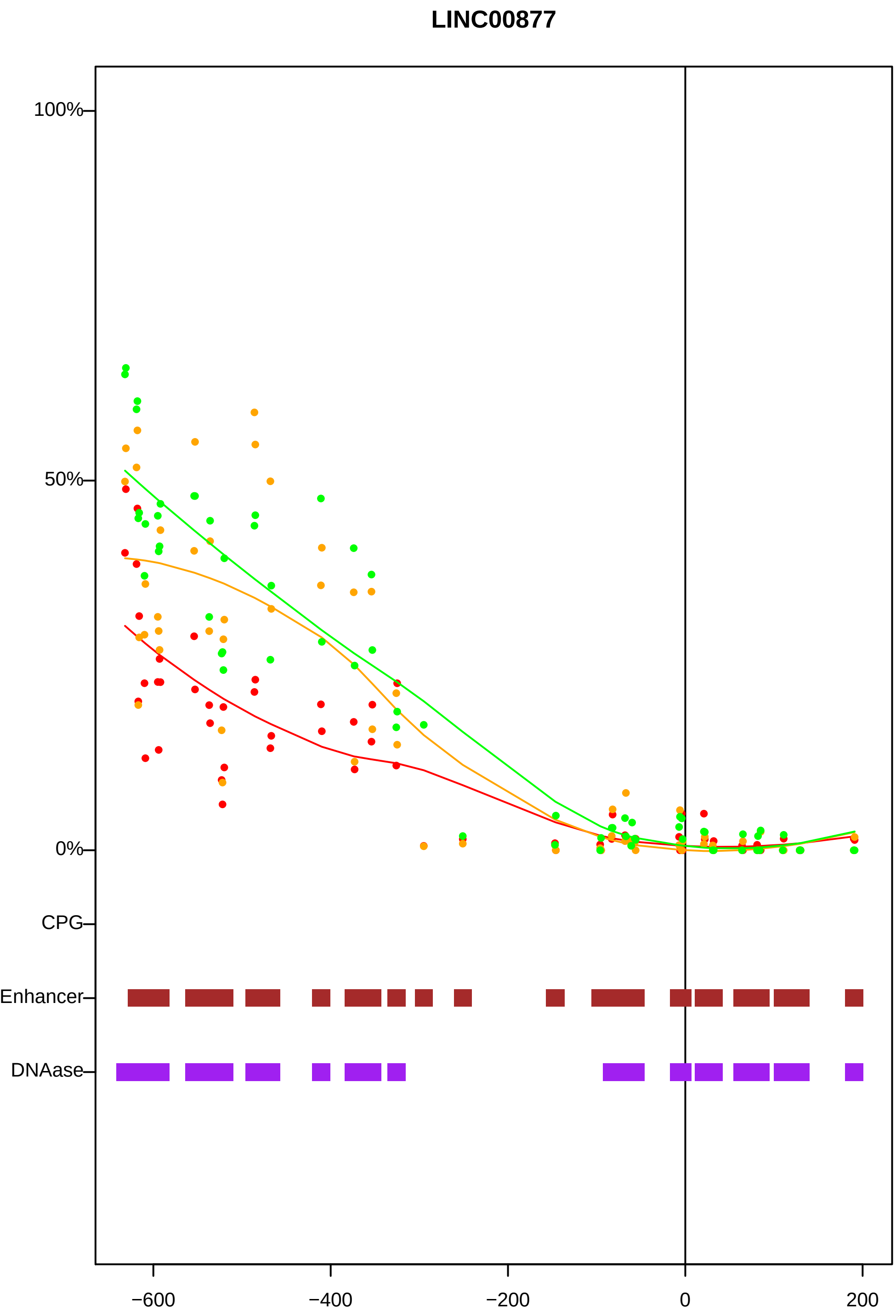

LINC00877
100%
50%
0%
CPG
Enhancer
DNAase
−600
−400
−200
0
200

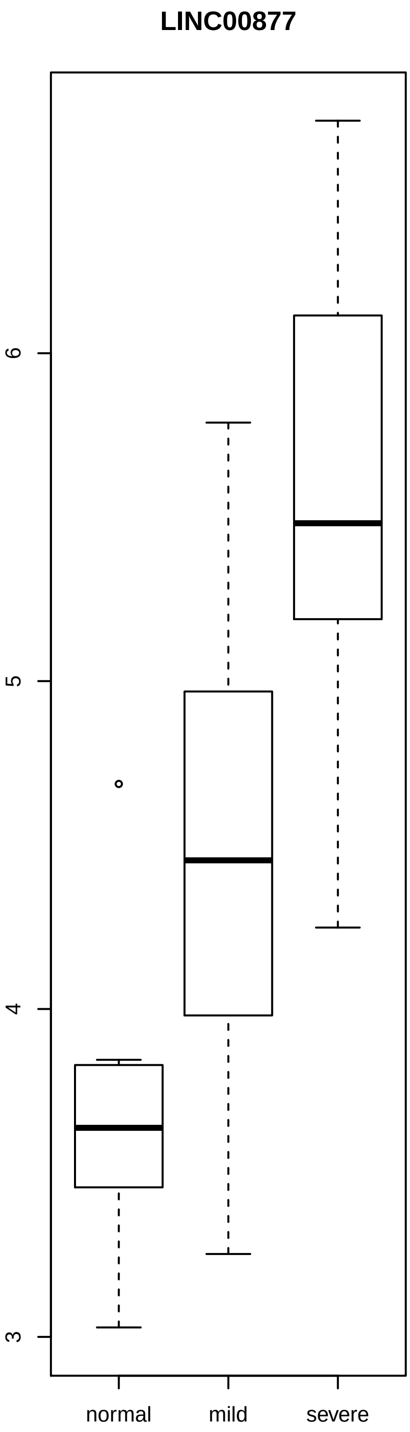

LINC00877
3
4
5
6
normal
mild
severe

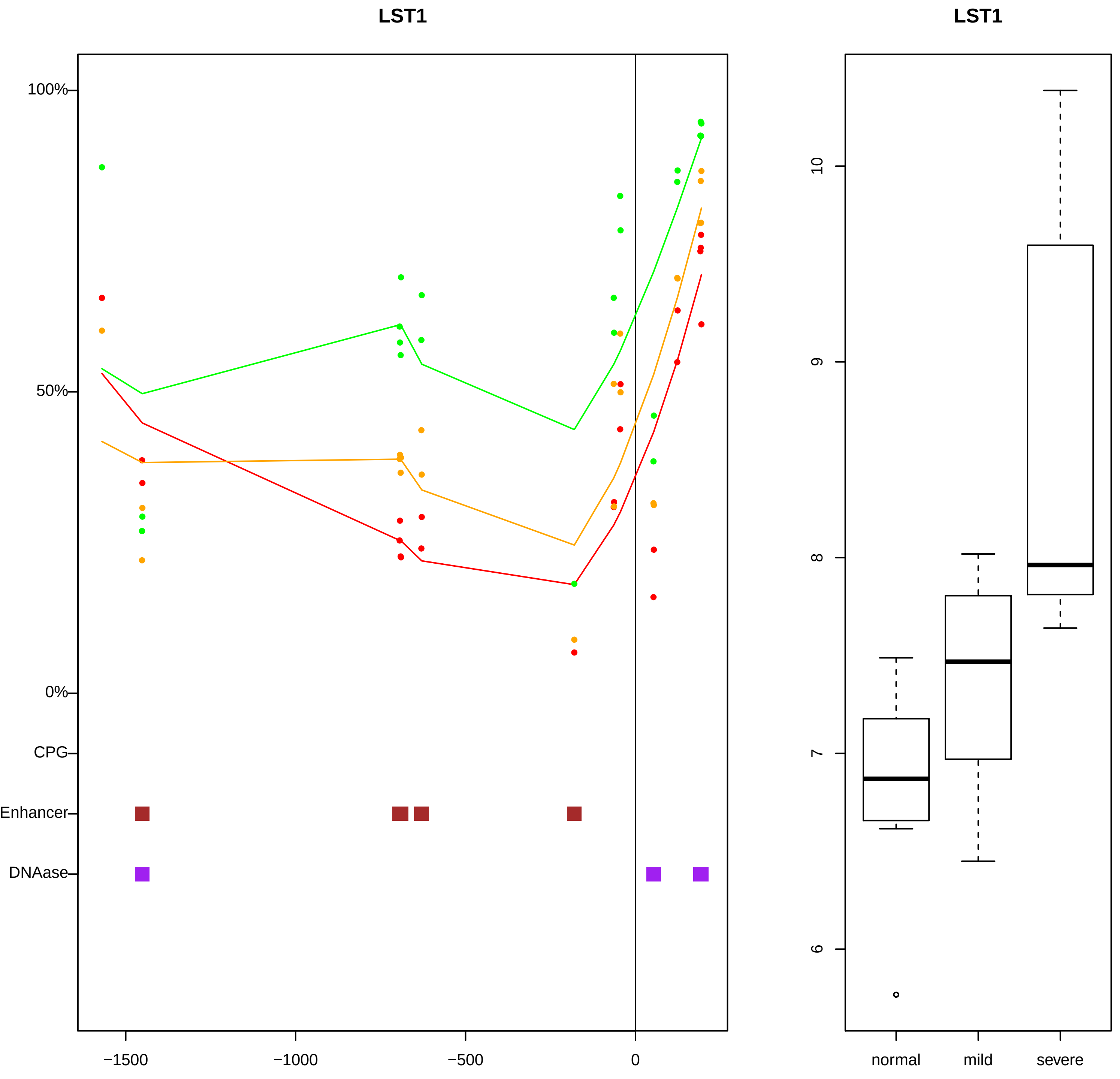
LST1
100%
50%
0%
CPG
Enhancer
DNAase
−1500
−1000
−500
0
LST1
10
9
8
7
6
normal
mild
severe

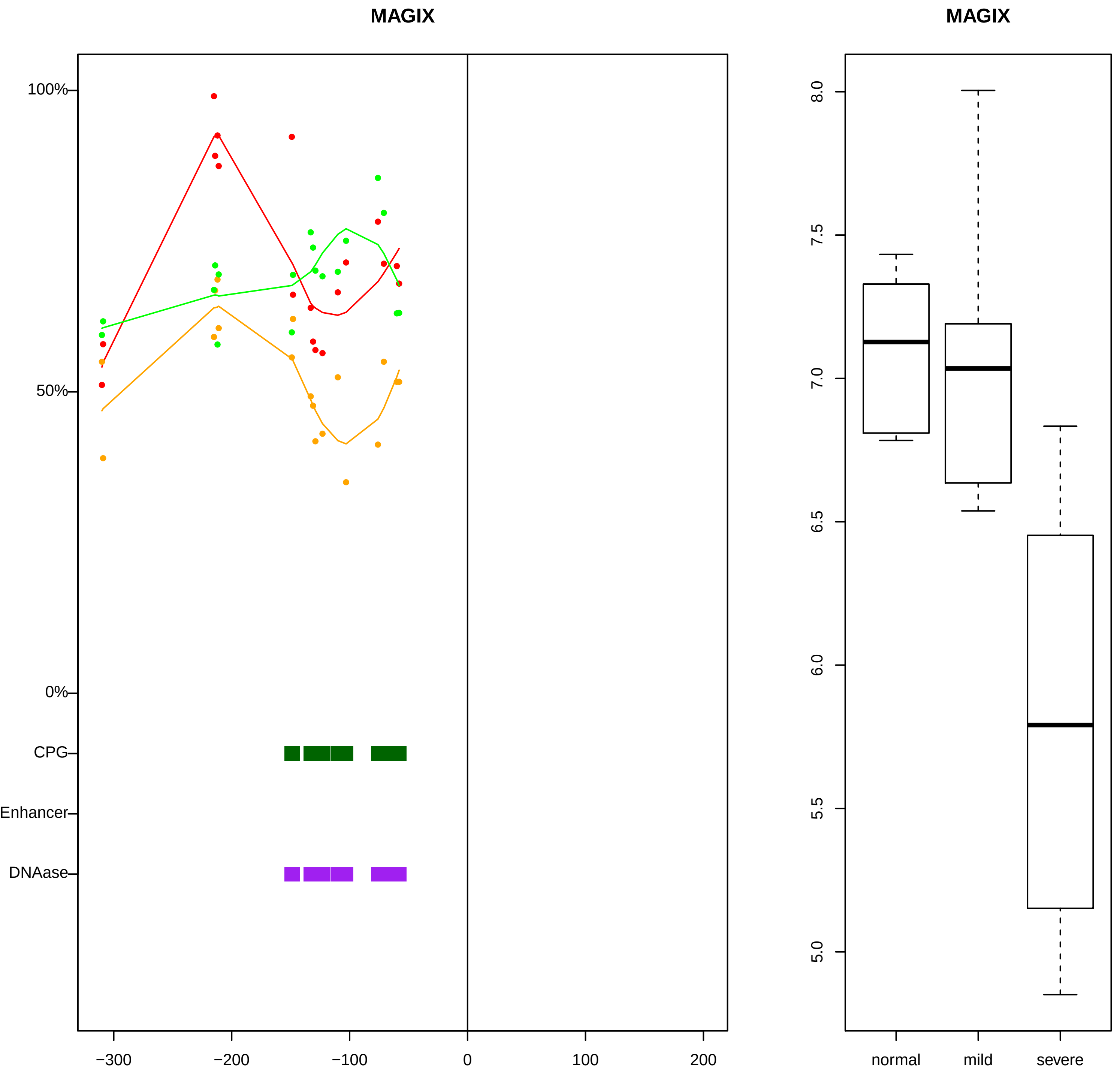
MAGIX
100%
50%
0%
CPG
Enhancer
DNAase
−300
−200
−100
0
100
200
MAGIX
8.0
7.5
7.0
6.5
6.0
5.5
5.0
normal
mild
severe

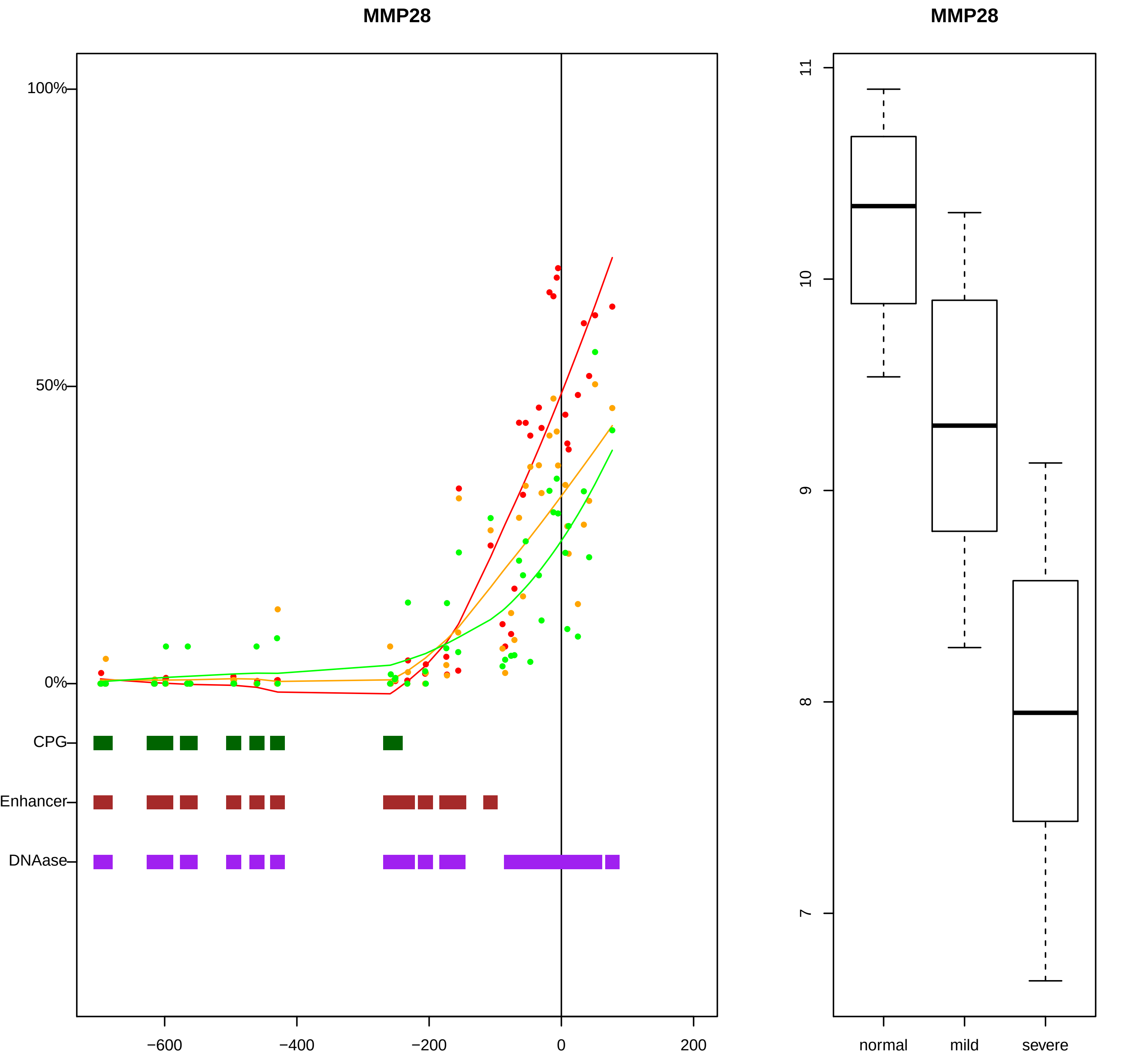
MMP28
100%
50%
0%
CPG
Enhancer
DNAase
−600
−400
−200
0
200
MMP28
11
10
9
8
7
normal
mild
severe

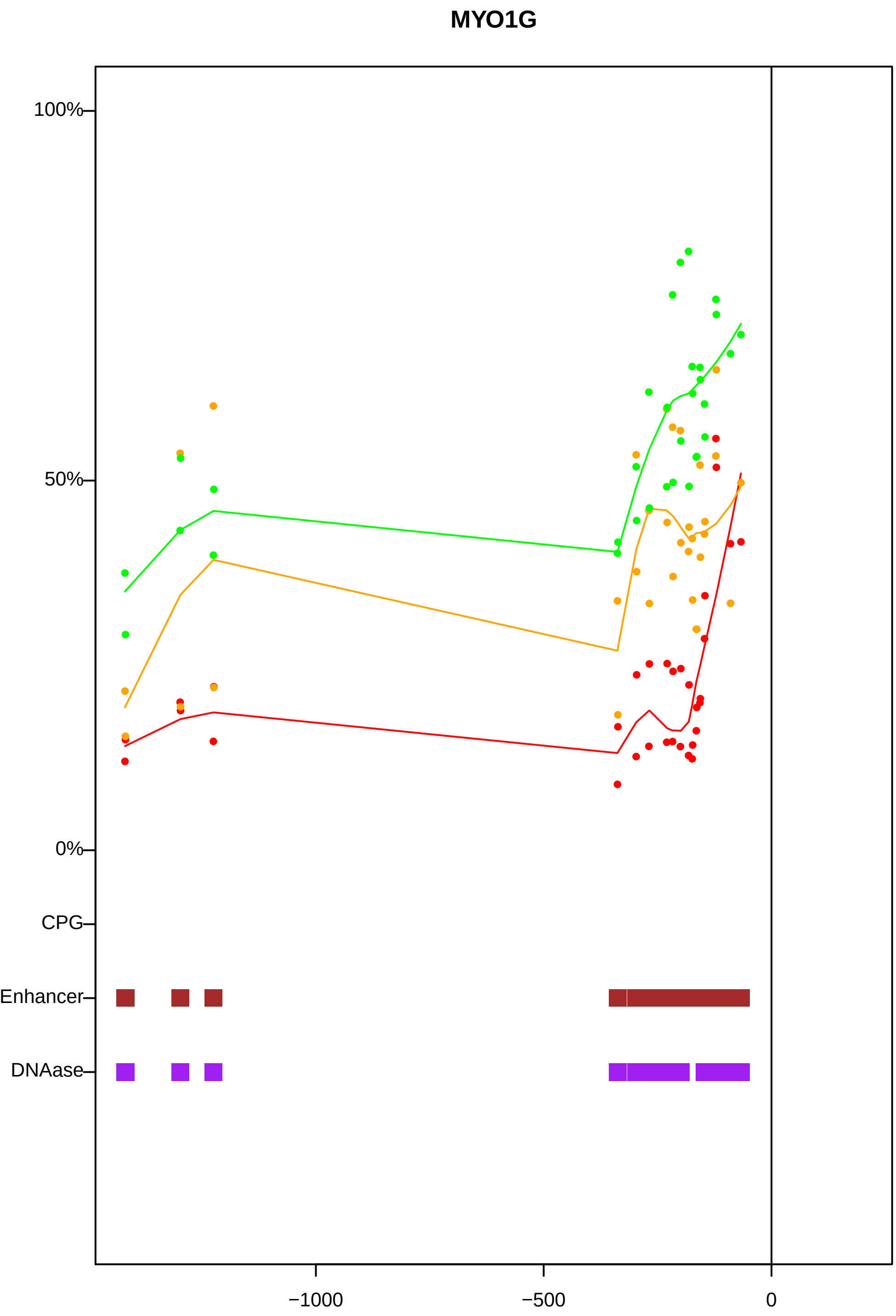
MYO1G
100%
50%
0%
CPG
Enhancer
DNAase
−1000
−500
0

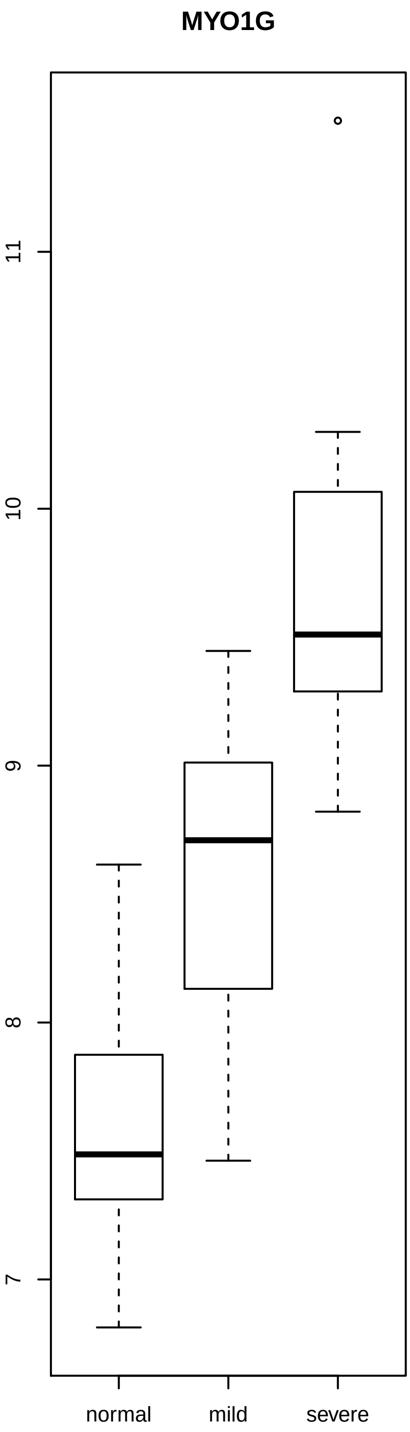
MYO1G
11
10
9
8
7
normal
mild
severe

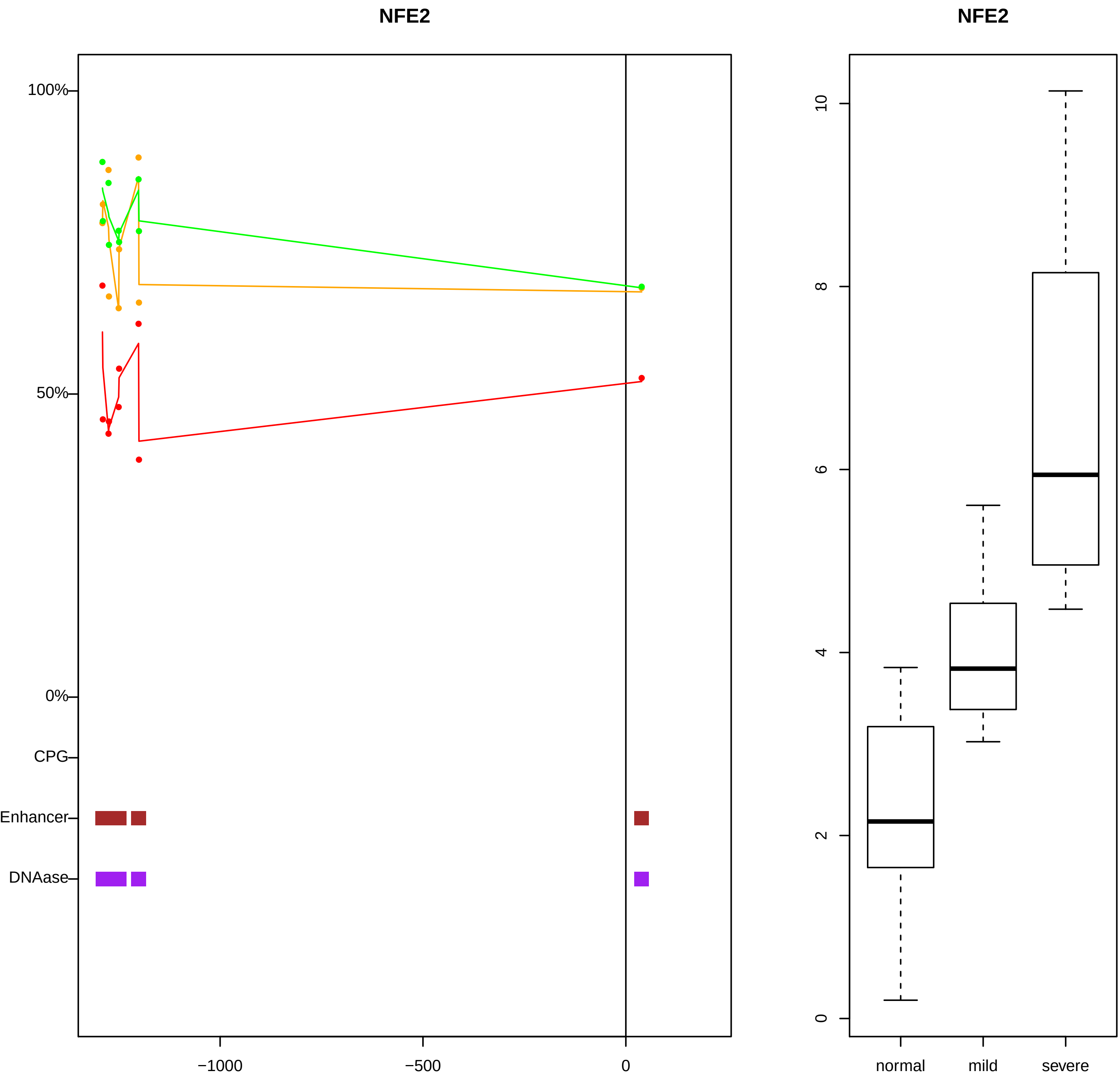
NFE2
100%
50%
0%
CPG
Enhancer
DNAase
−1000
−500
0
NFE2
0
2
4
6
8
10
normal
mild
severe

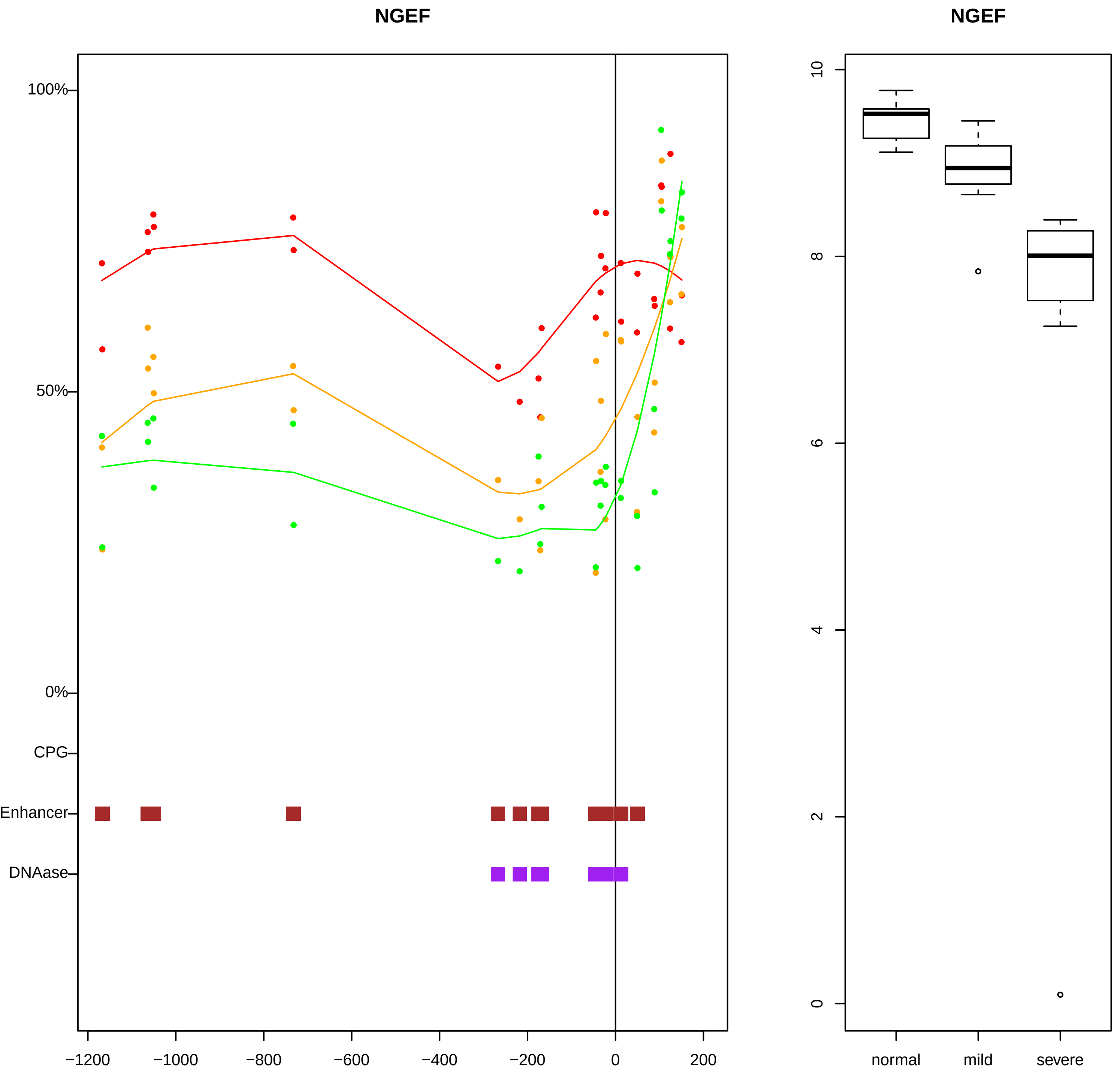
NGEF
100%
50%
0%
CPG
Enhancer
DNAase
−1200
−1000
−800
−600
−400
−200
0
200
NGEF
10
8
6
4
2
0
normal
mild
severe

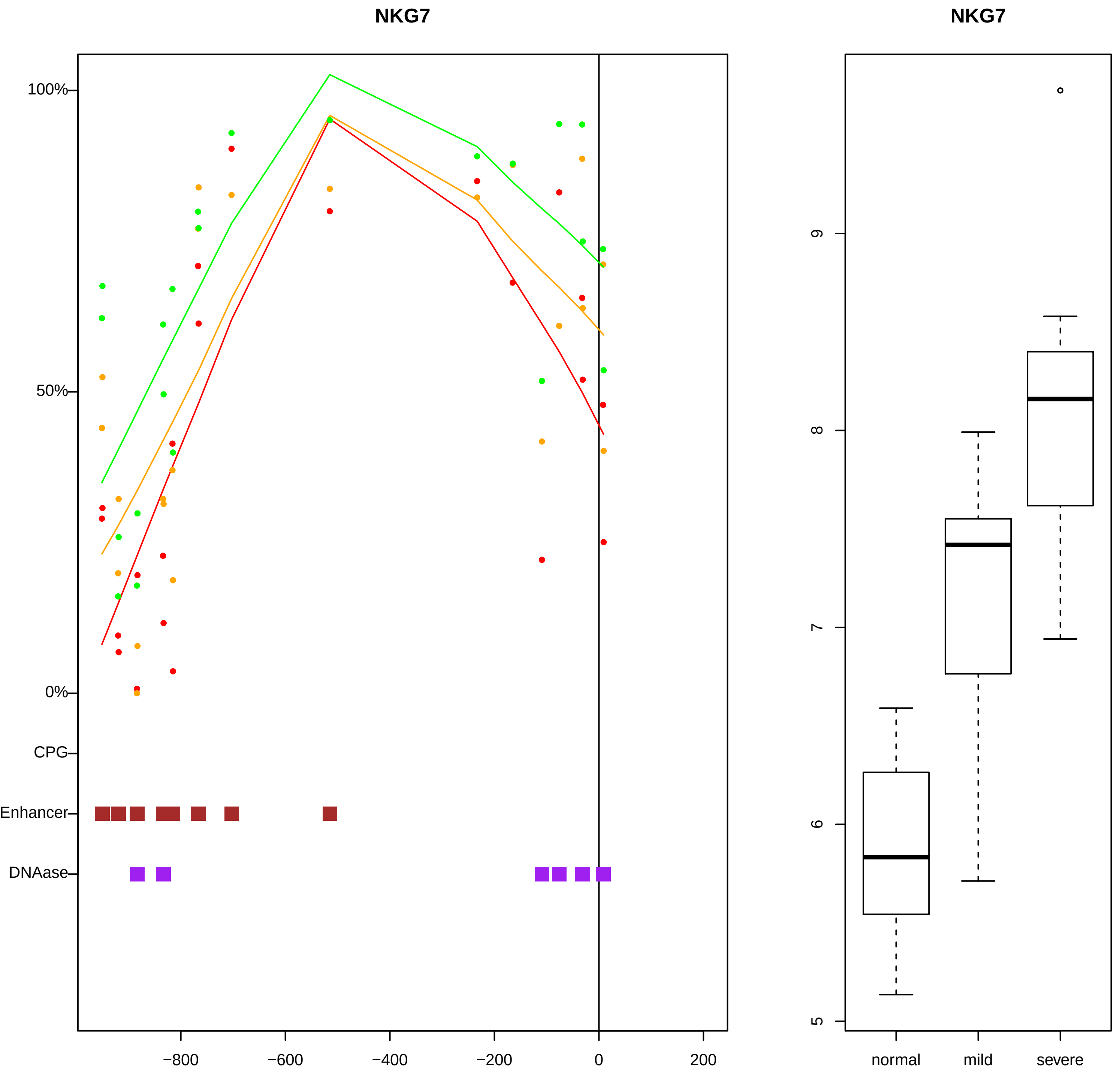
NKG7
100%
50%
0%
CPG
Enhancer
DNAase
−800
−600
−400
−200
0
200
NKG7
9
8
7
6
5
normal
mild
severe

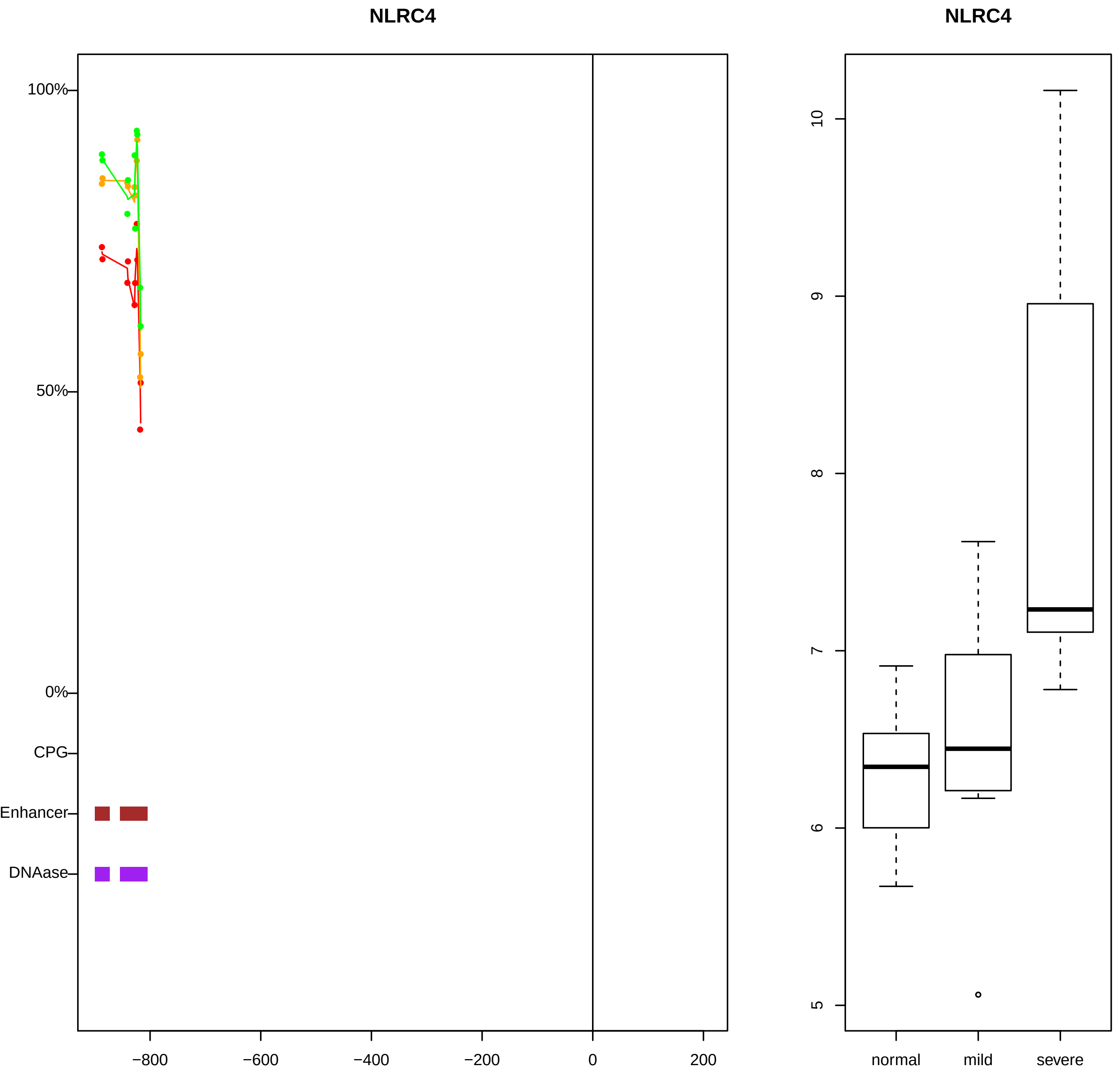

NLRC4
100%
50%
0%
CPG
Enhancer
DNAase
−800
−600
−400
−200
0
200
NLRC4
10
9
8
7
6
5
normal
mild
severe

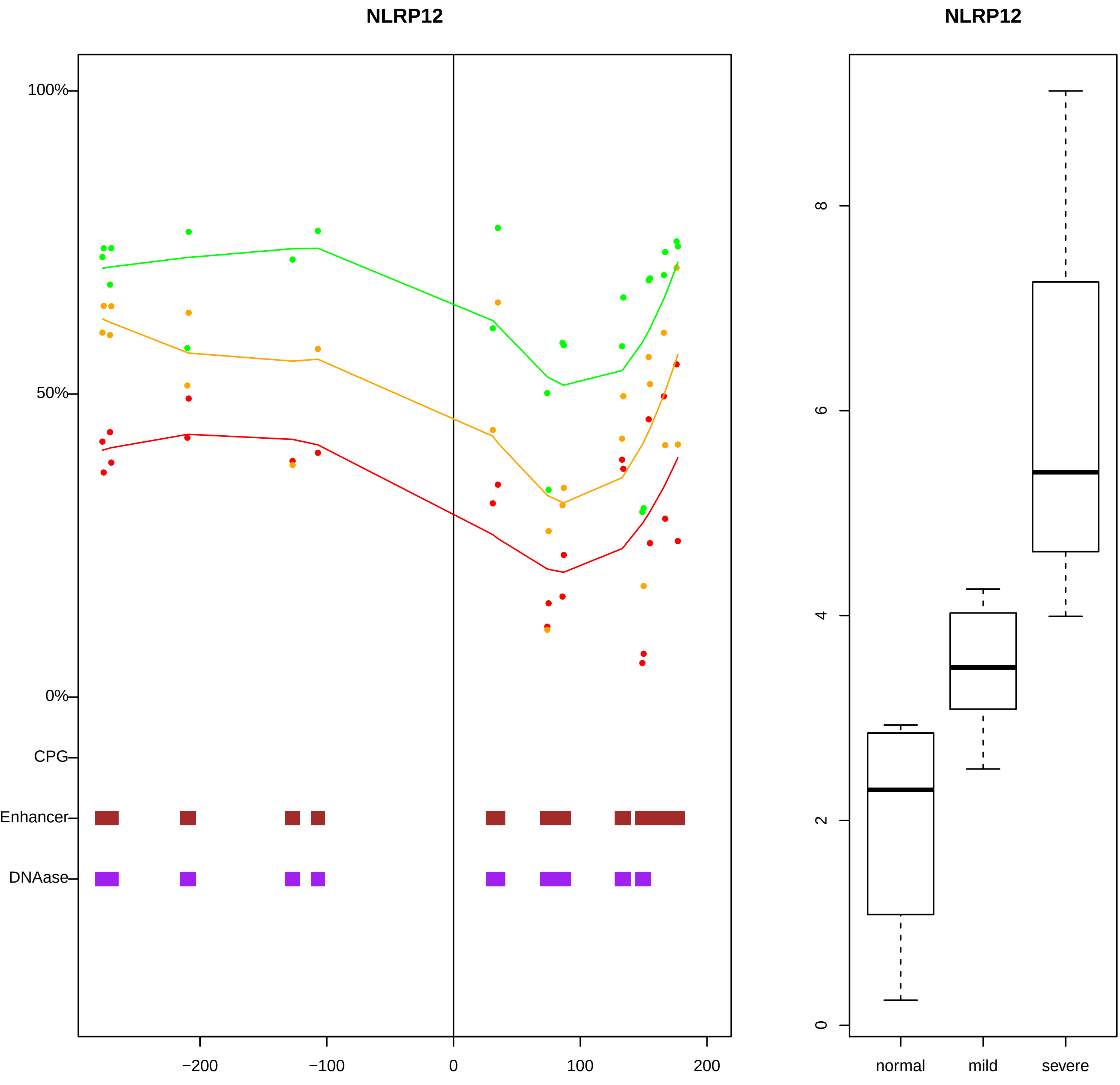
NLRP12
100%
50%
0%
CPG
Enhancer
DNAase
−200
−100
0
100
200
NLRP12
0
2
4
6
8
normal
mild
severe

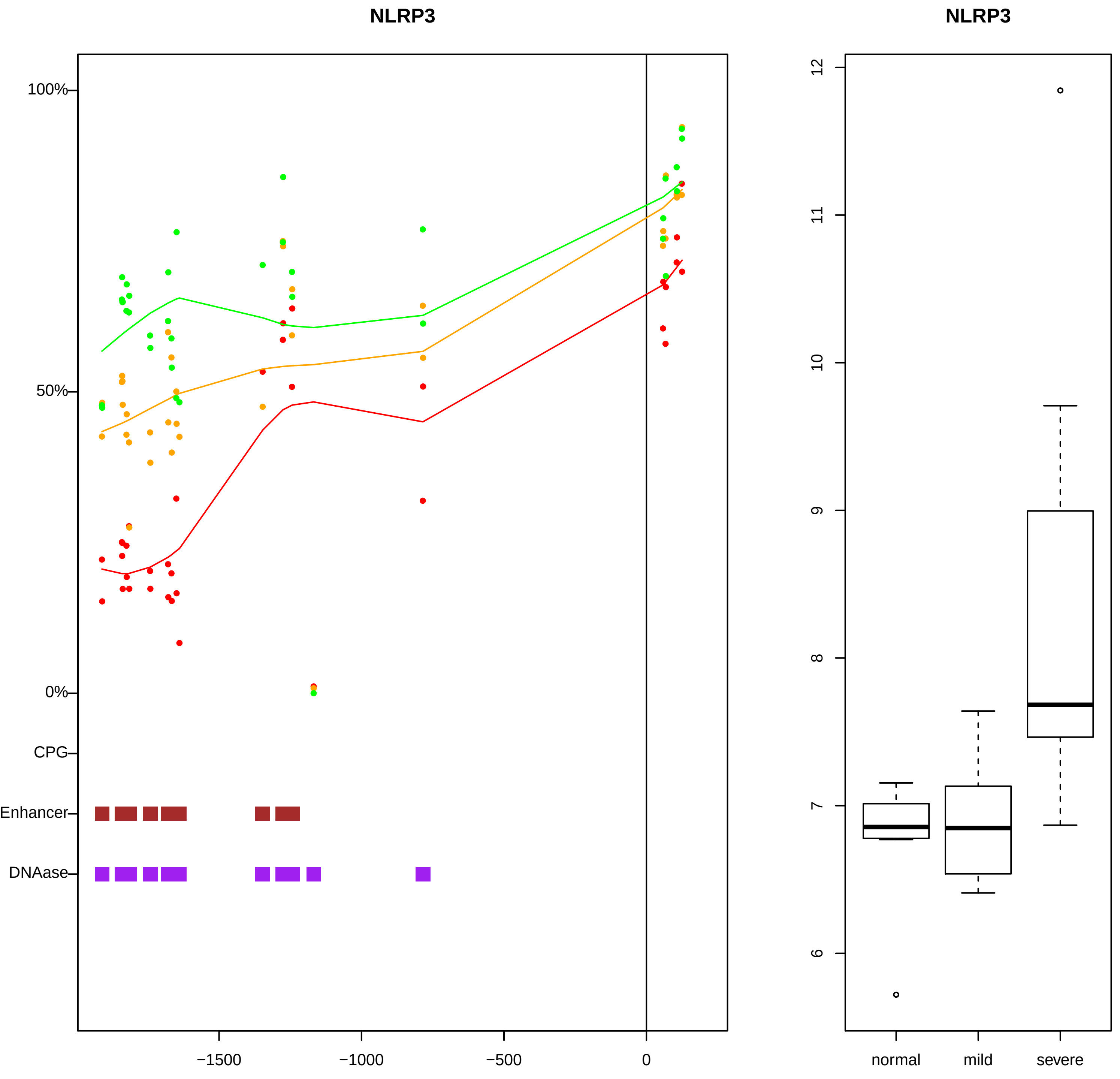
NLRP3
100%
50%
0%
CPG
Enhancer
DNAase
−1500
−1000
−500
0
NLRP3
12
11
10
9
8
7
6
normal
mild
severe

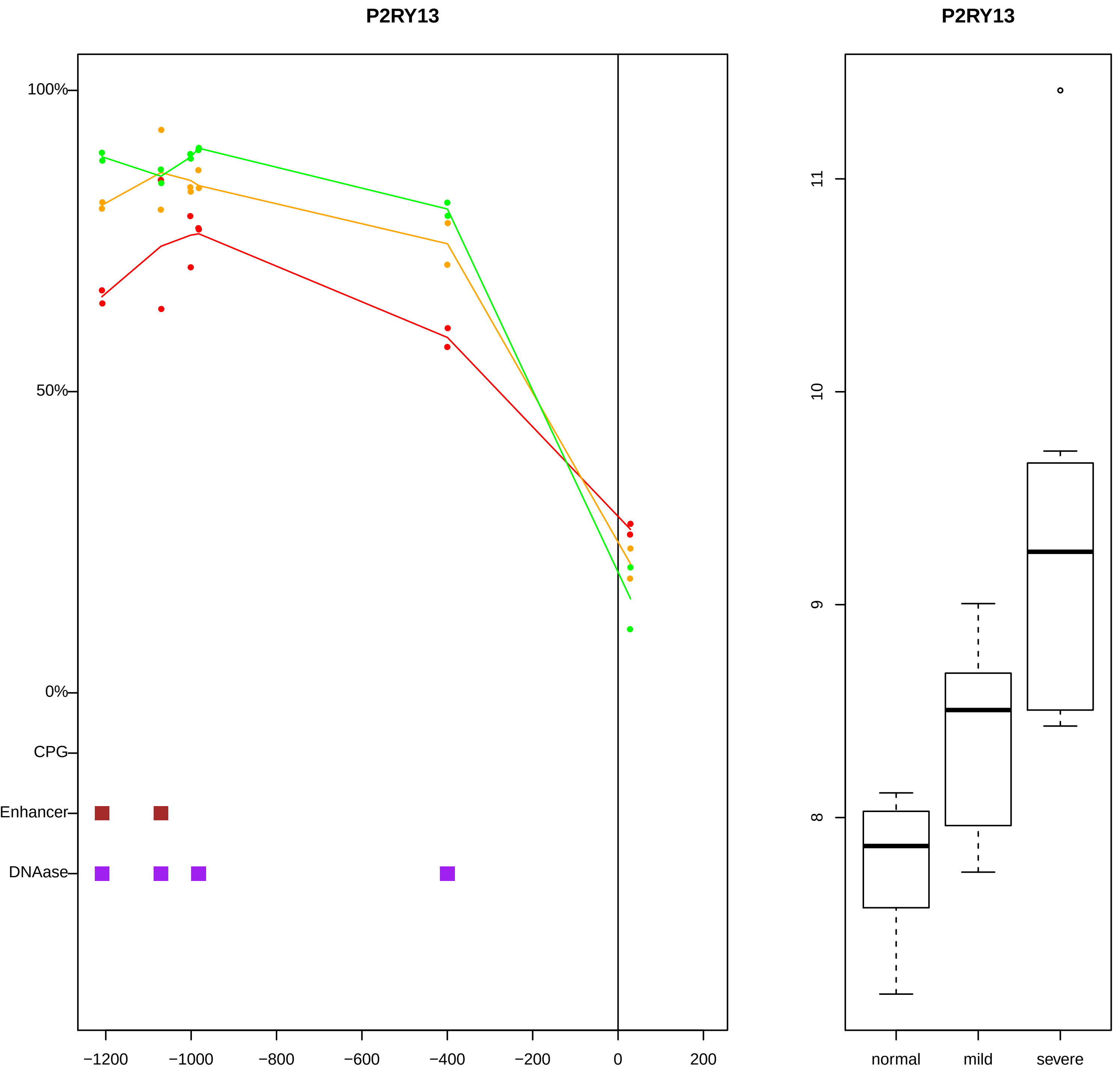

P2RY13
100%
50%
0%
CPG
Enhancer
DNAase
−1200
−1000
−800
−600
−400
−200
0
200
P2RY13
11
10
9
8
normal
mild
severe

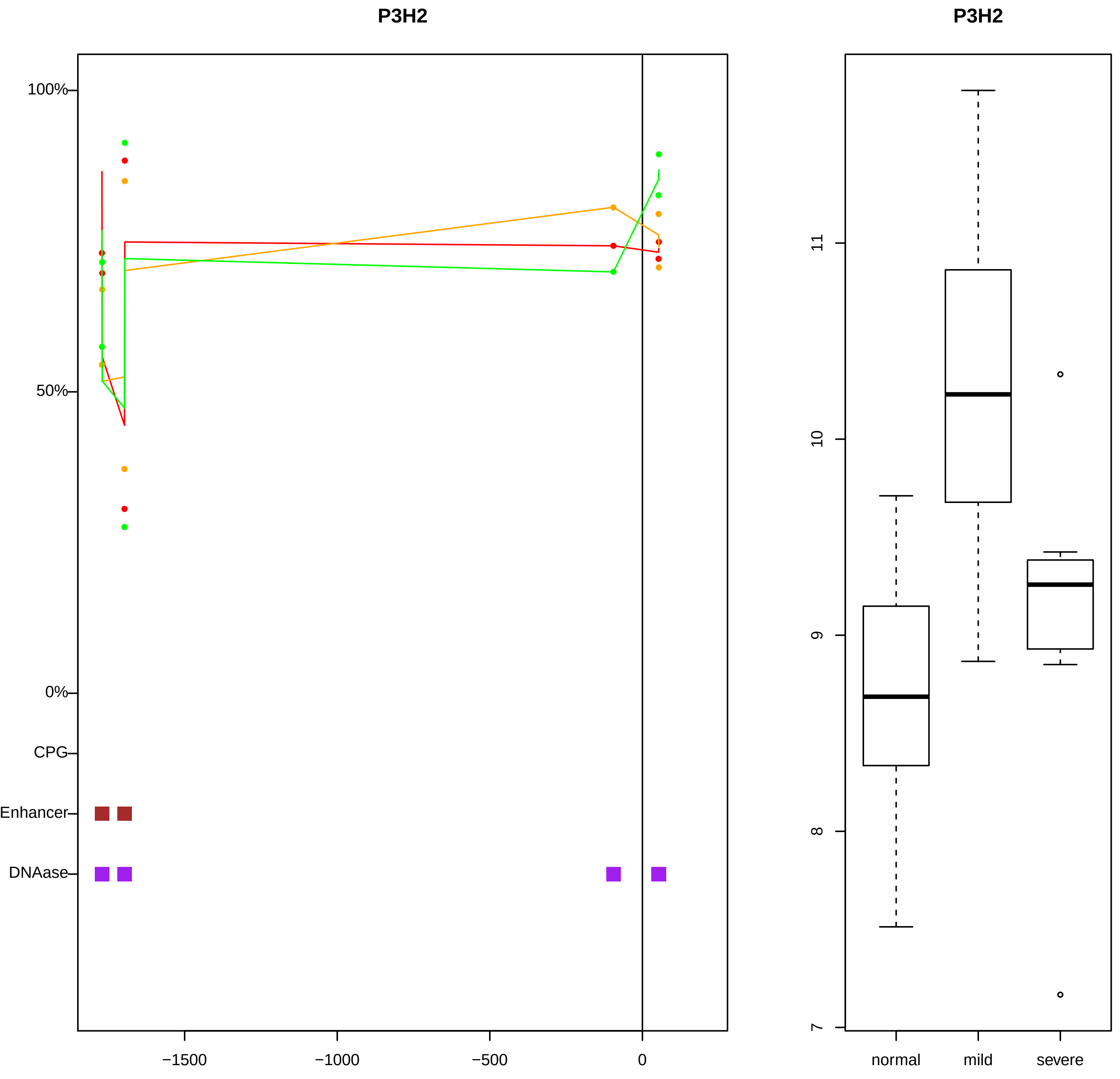

P3H2
100%
50%
0%
CPG
Enhancer
DNAase
−1500
−1000
−500
0
P3H2
11
10
9
8
7
normal
mild
severe

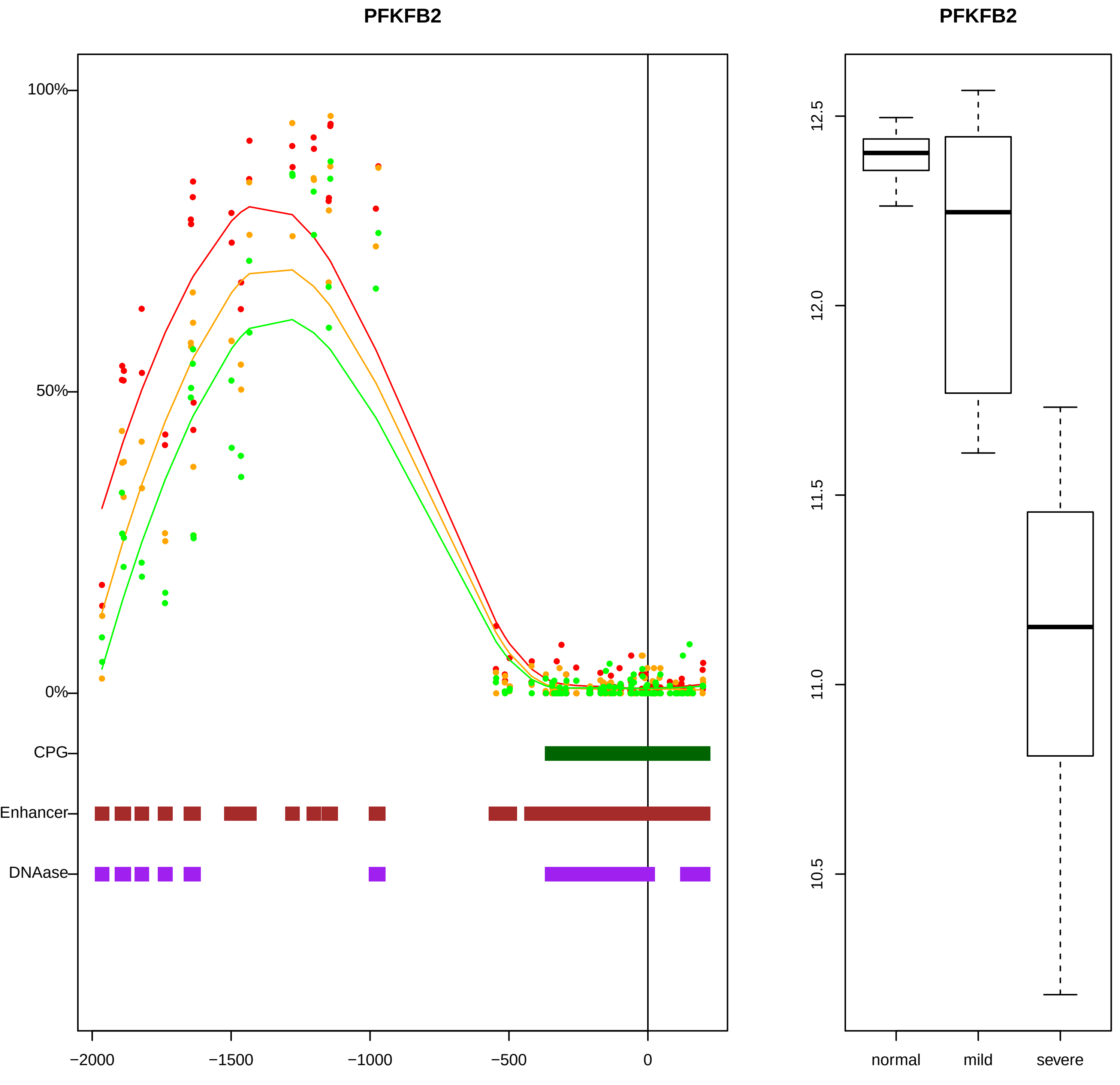
PFKFB2
100%
50%
0%
CPG
Enhancer
DNAase
−2000
−1500
−1000
−500
0
PFKFB2
12.5
12.0
11.5
11.0
10.5
normal
mild
severe

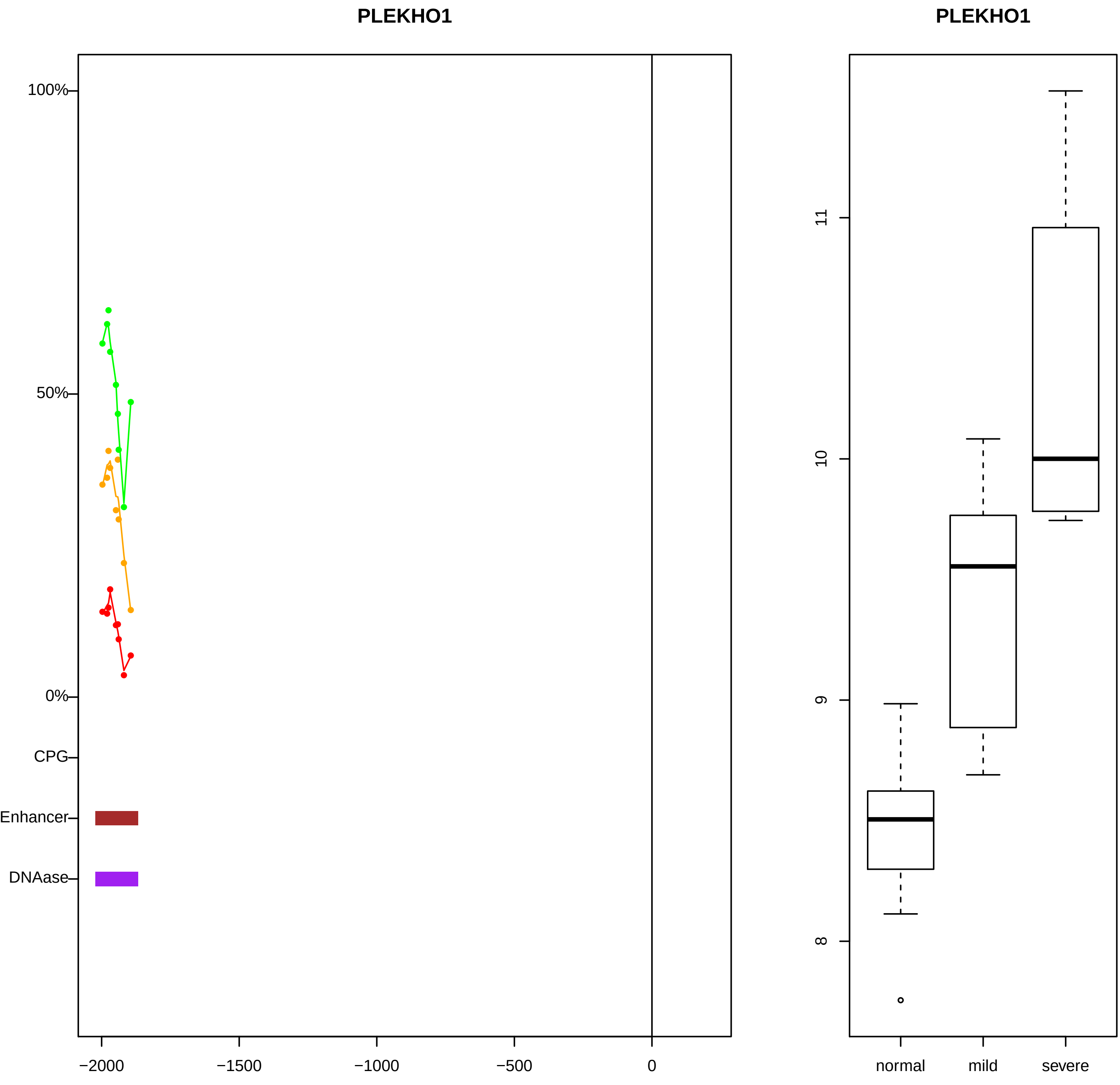
PLEKHO1
100%
50%
0%
CPG
Enhancer
DNAase
−2000
−1500
−1000
−500
0
PLEKHO1
11
10
9
8
normal
mild
severe

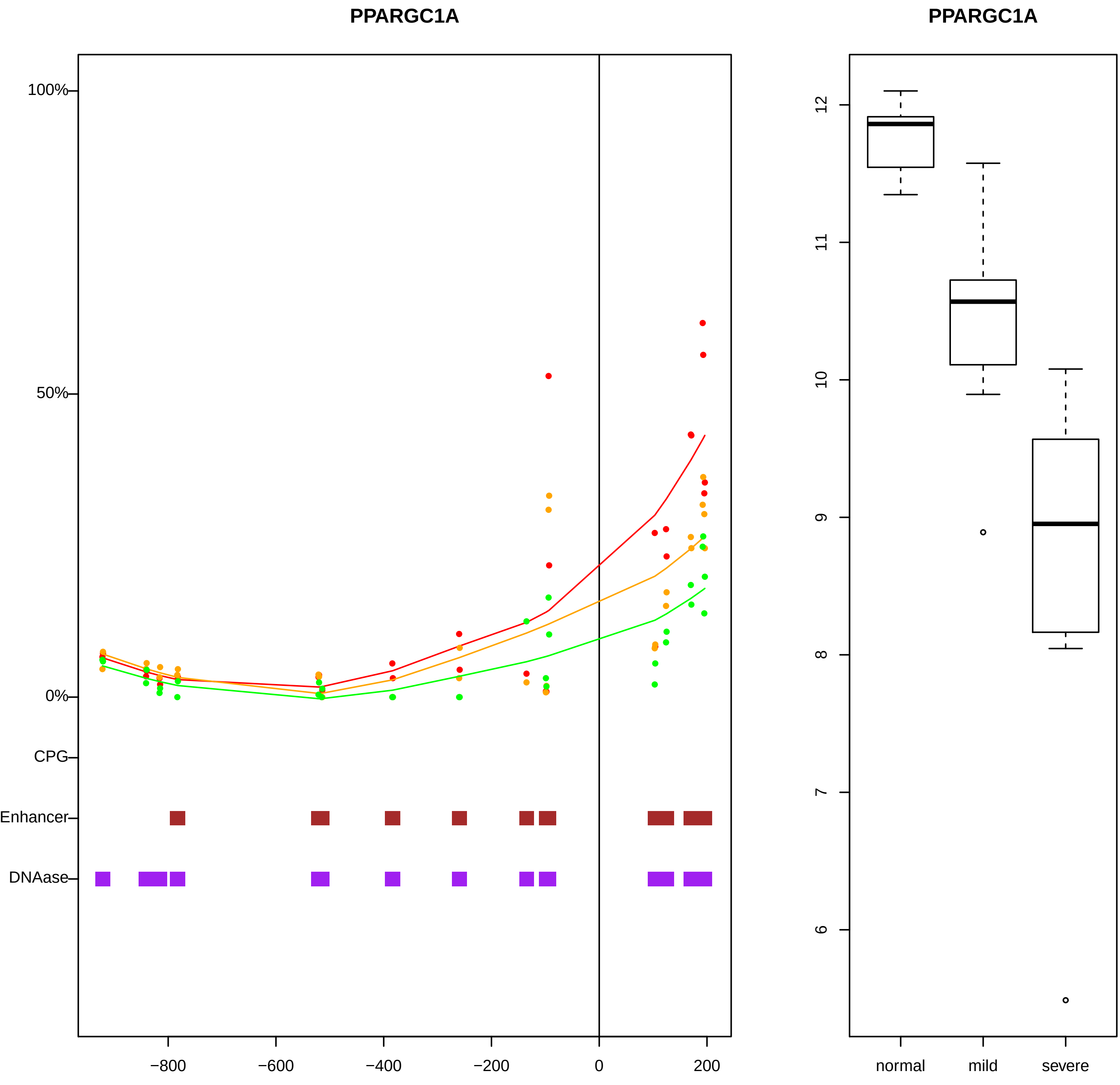
PPARGC1A
100%
50%
0%
CPG
Enhancer
DNAase
−800
−600
−400
−200
0
200
PPARGC1A
12
11
10
9
8
7
6
normal
mild
severe

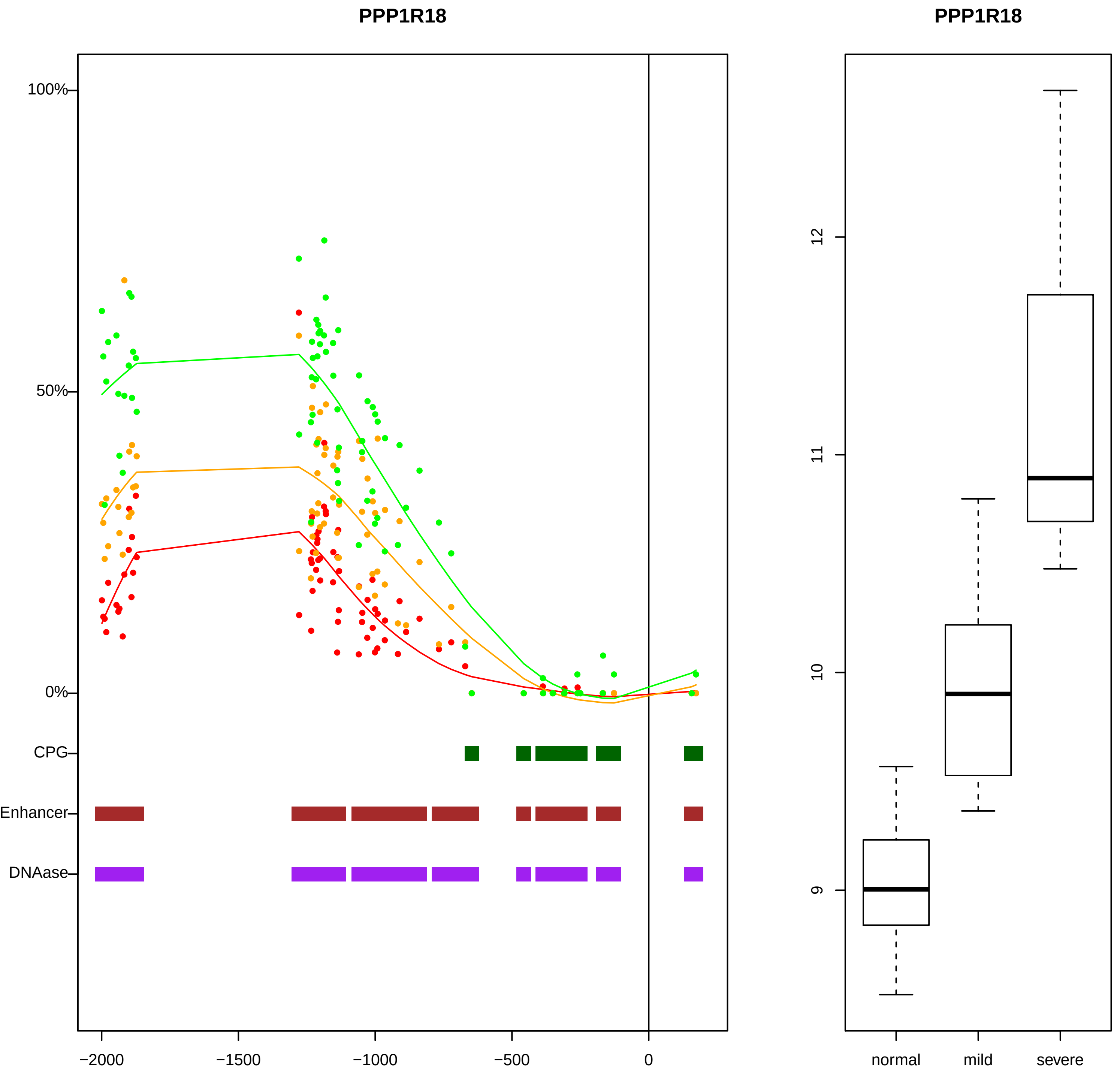
PPP1R18
100%
50%
0%
CPG
Enhancer
DNAase
−2000
−1500
−1000
−500
0
PPP1R18
12
11
10
9
normal
mild
severe

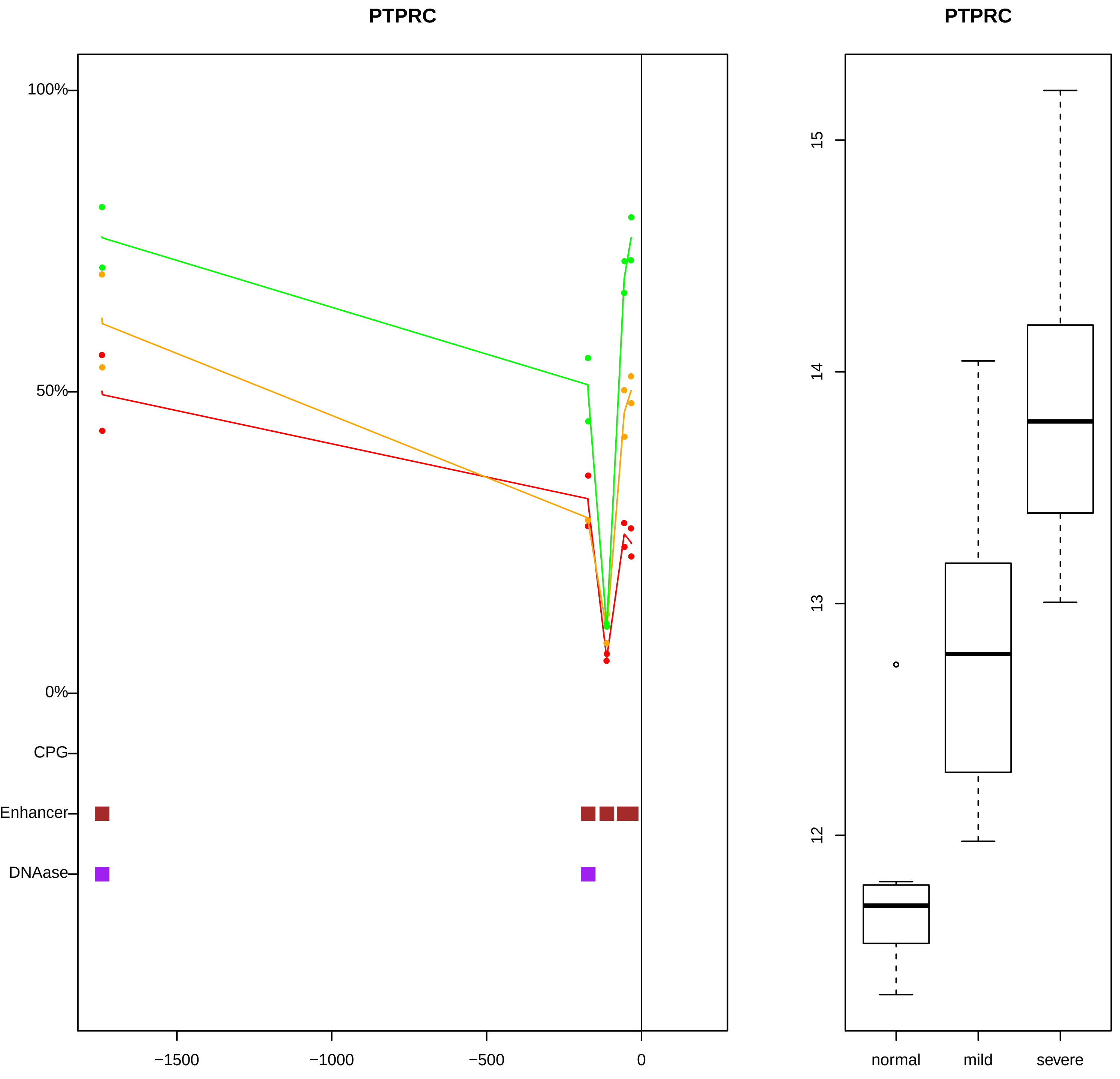

PTPRC
100%
50%
0%
CPG
Enhancer
DNAase
−1500
−1000
−500
0
PTPRC
15
14
13
12
normal
mild
severe

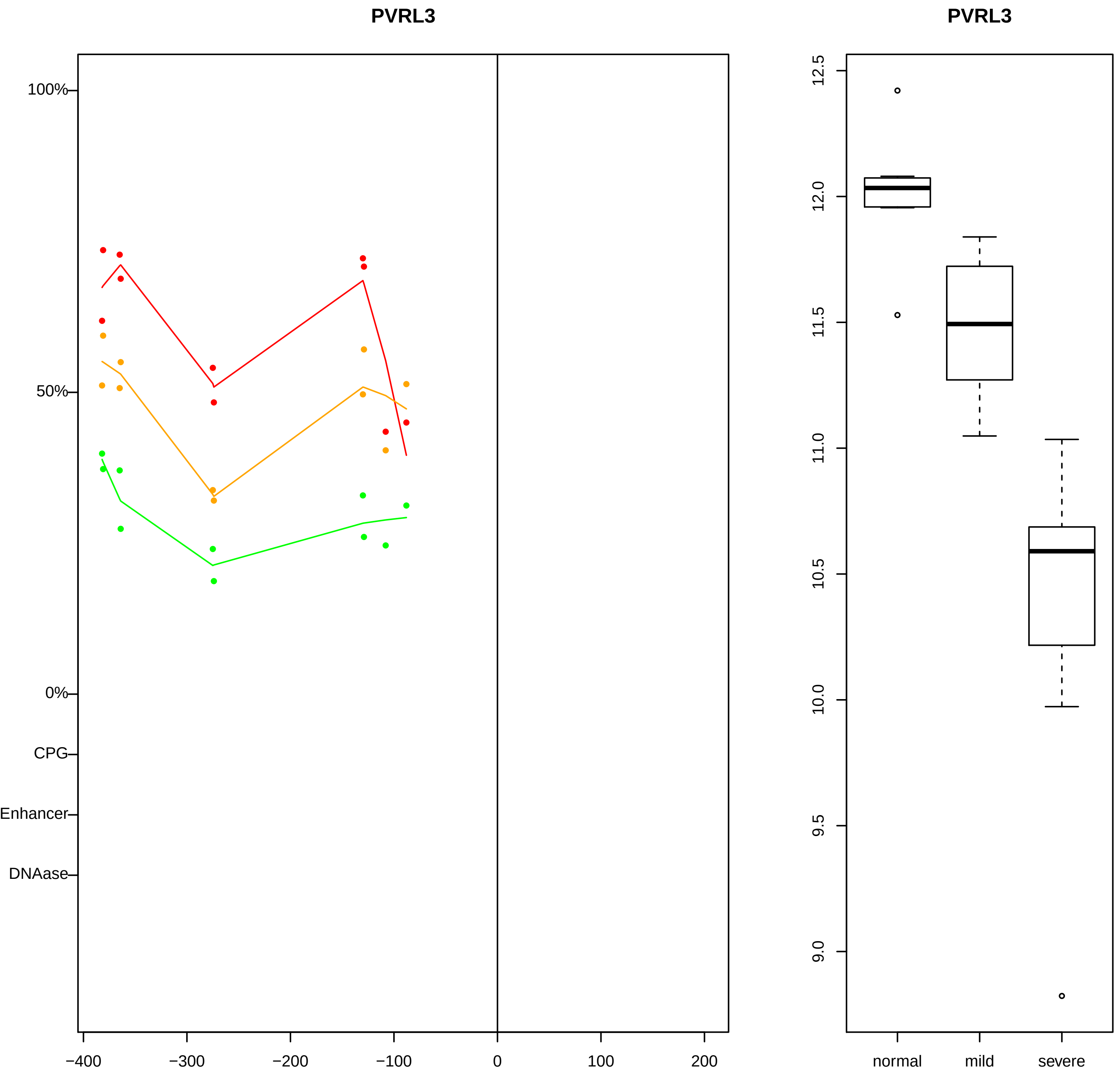
PVRL3
100%
50%
0%
CPG
Enhancer
DNAase
−400
−300
−200
−100
0
100
200
PVRL3
12.5
12.0
11.5
11.0
10.5
10.0
9.5
9.0
normal
mild
severe

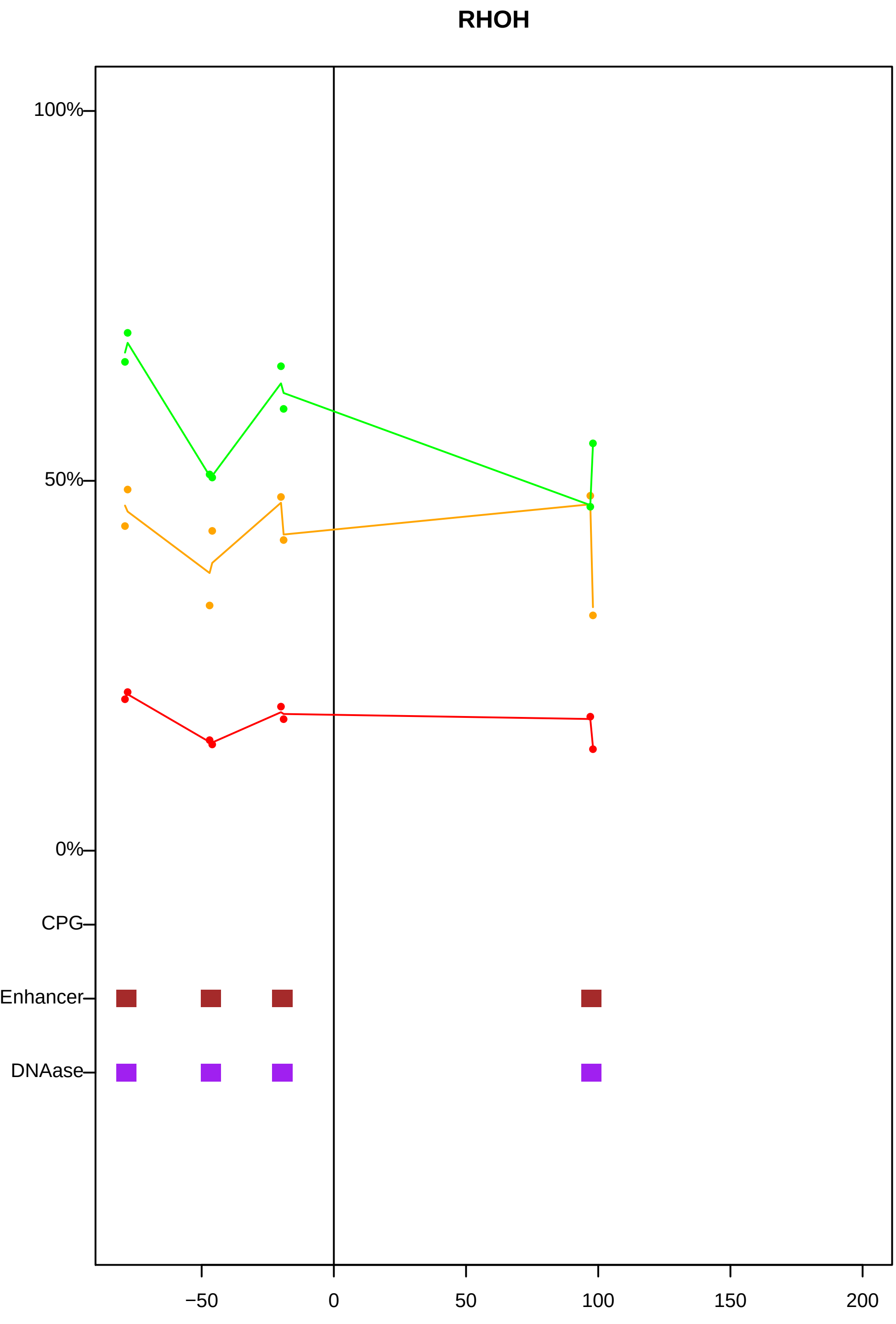
RHOH
100%
50%
0%
CPG
Enhancer
DNAase
−50
0
50
100
150
200

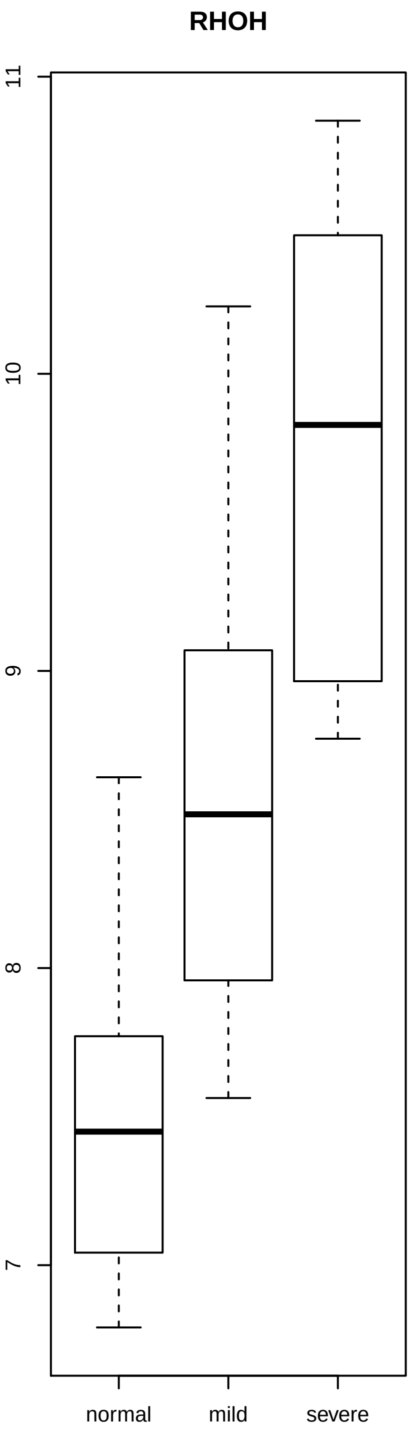
RHOH
11
10
9
8
7
normal
mild
severe

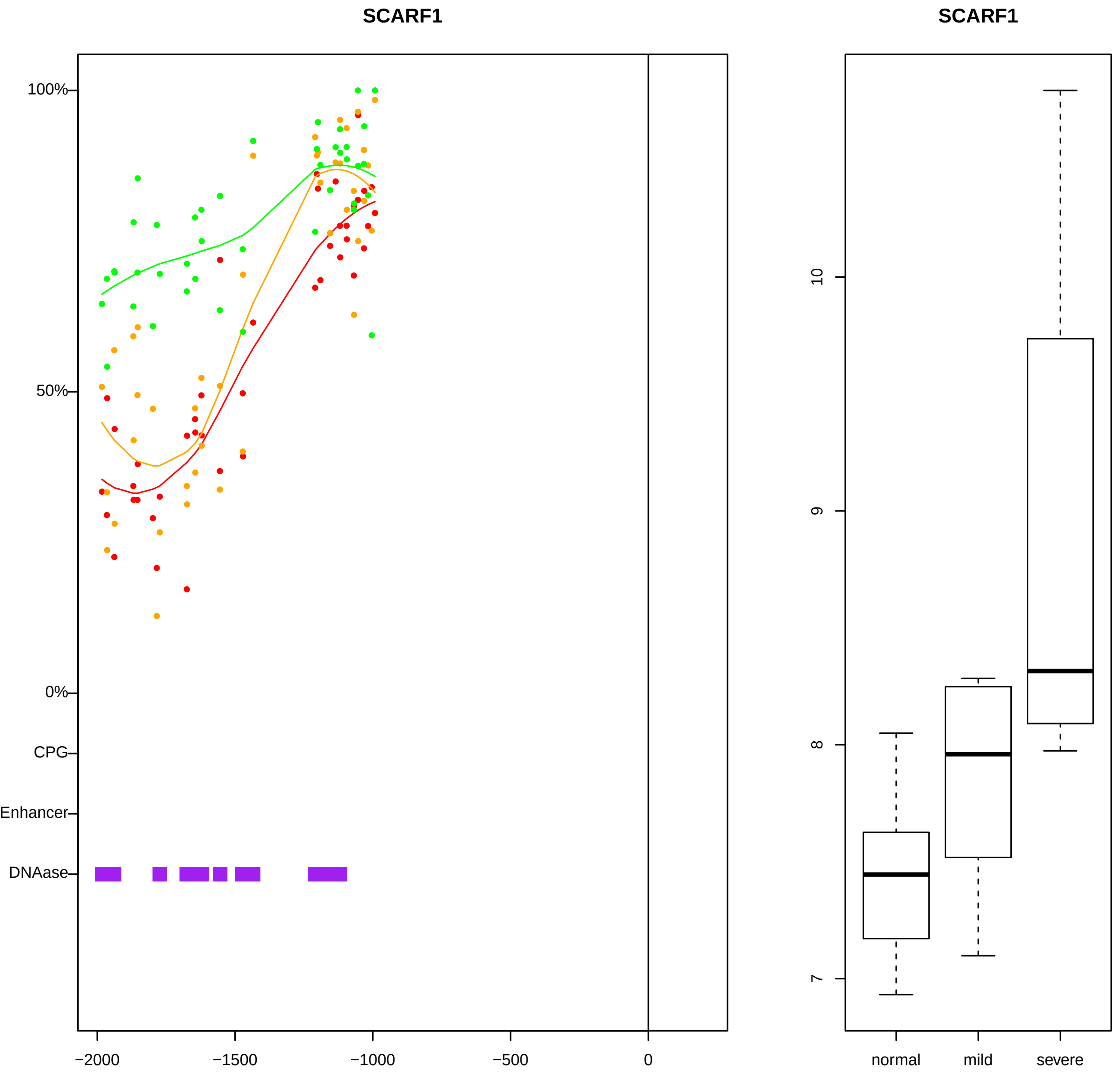
SCARF1
100%
50%
0%
CPG
Enhancer
DNAase
−2000
−1500
−1000
−500
0
SCARF1
10
9
8
7
normal
mild
severe

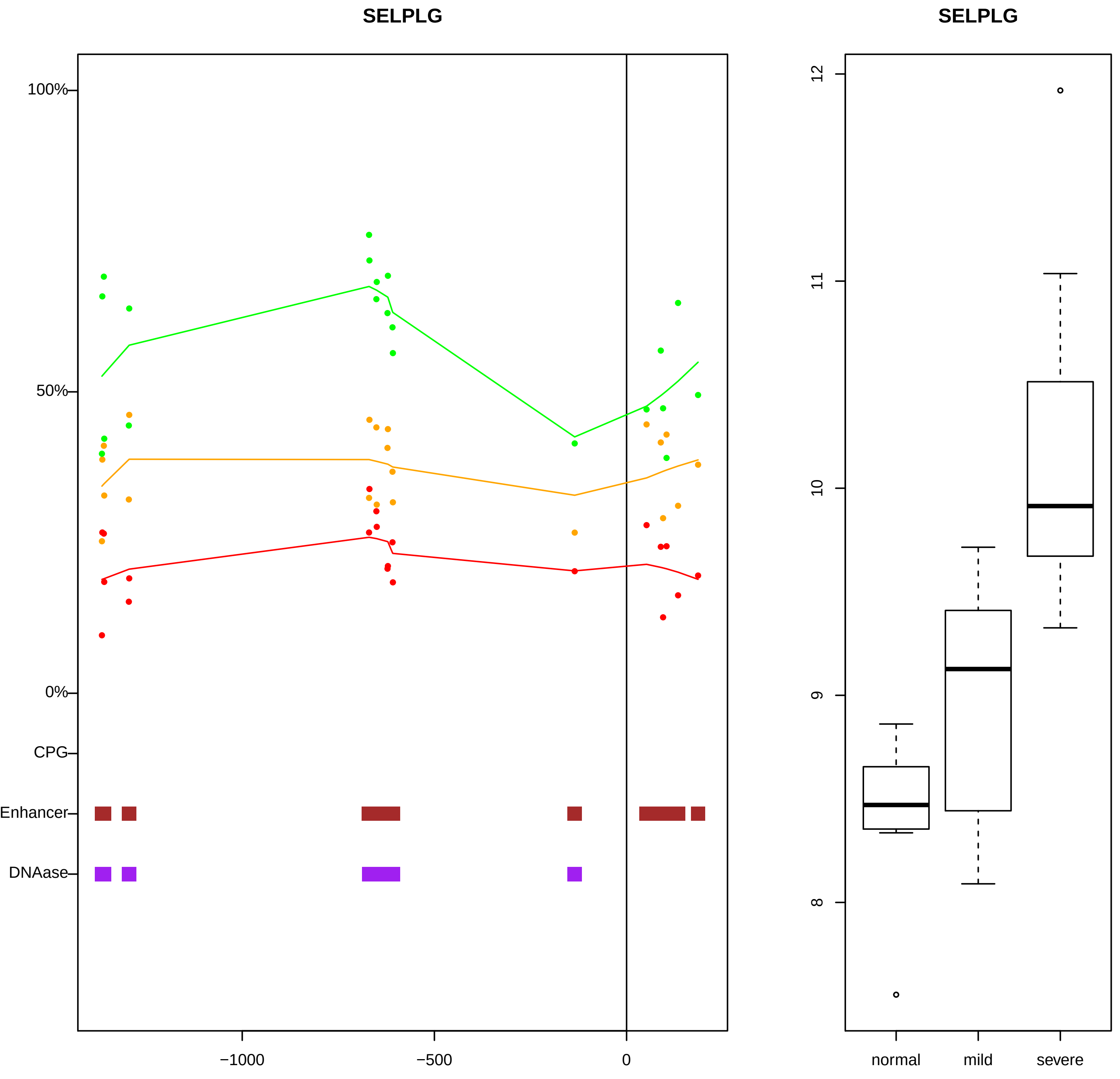
SELPLG
100%
50%
0%
CPG
Enhancer
DNAase
−1000
−500
0
SELPLG
12
11
10
9
8
normal
mild
severe

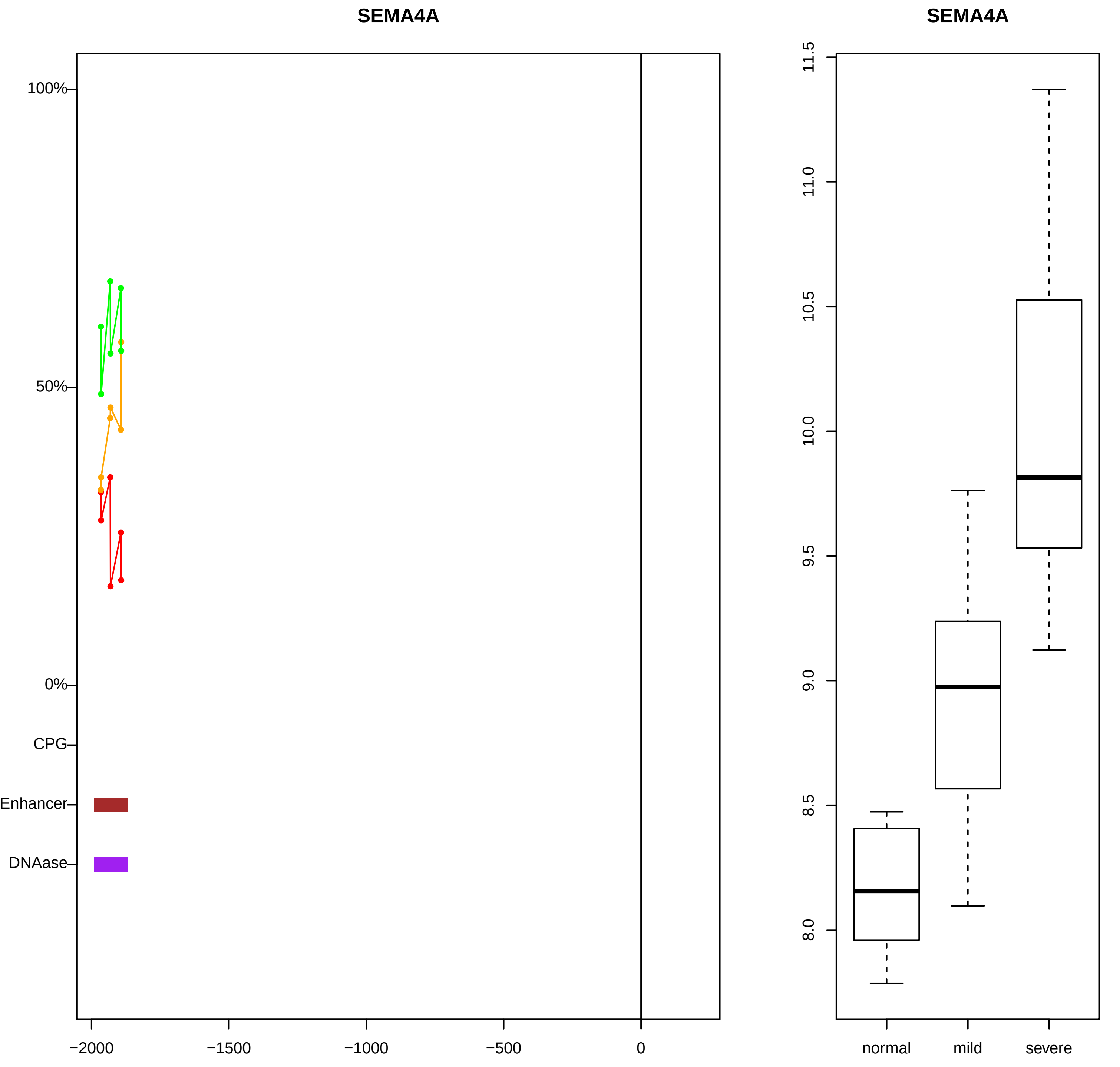
SEMA4A
100%
50%
0%
CPG
Enhancer
DNAase
−2000
−1500
−1000
−500
0
SEMA4A
11.5
11.0
10.5
10.0
9.5
9.0
8.5
8.0
normal
mild
severe

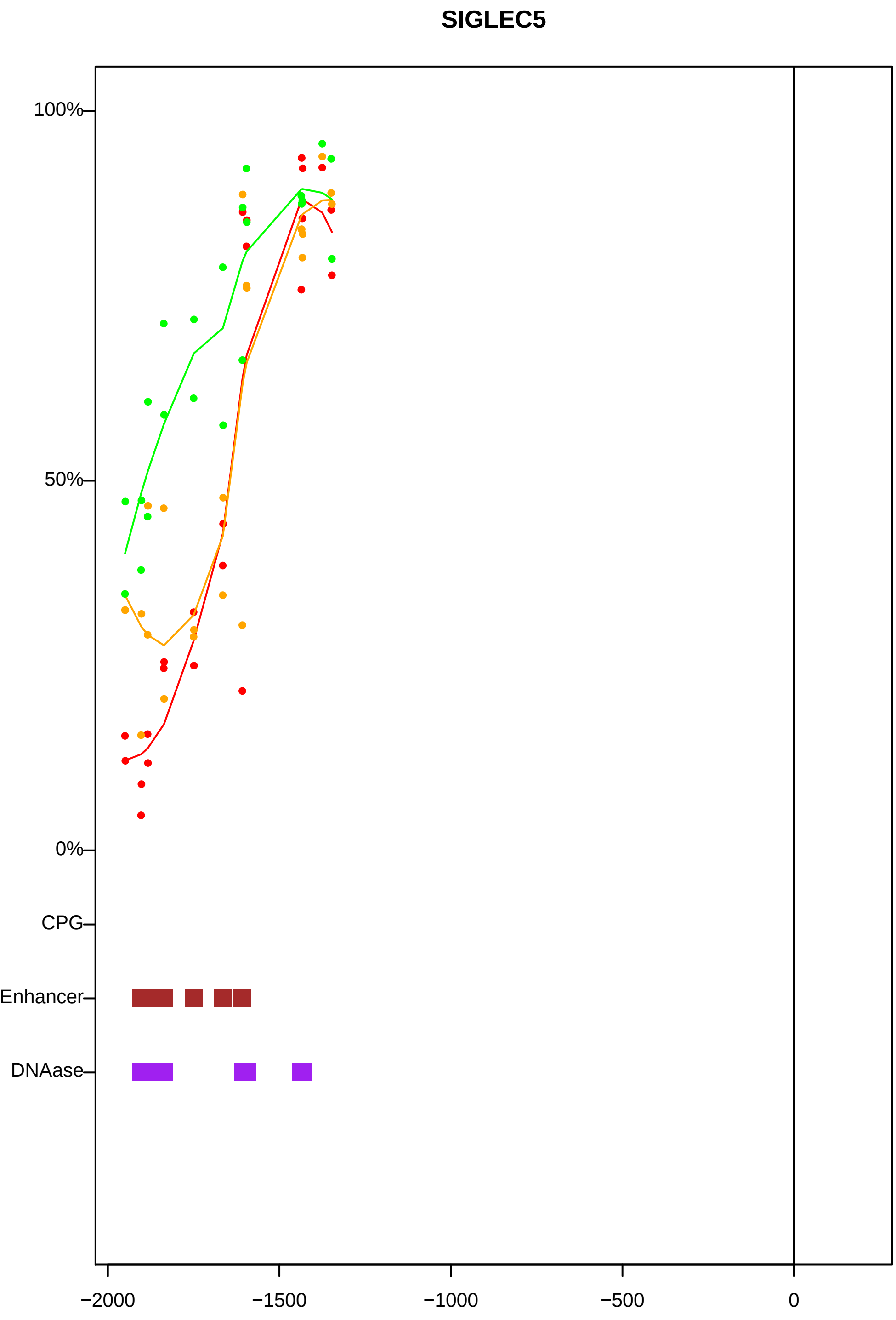
SIGLEC5
100%
50%
0%
CPG
Enhancer
DNAase
−2000
−1500
−1000
−500
0

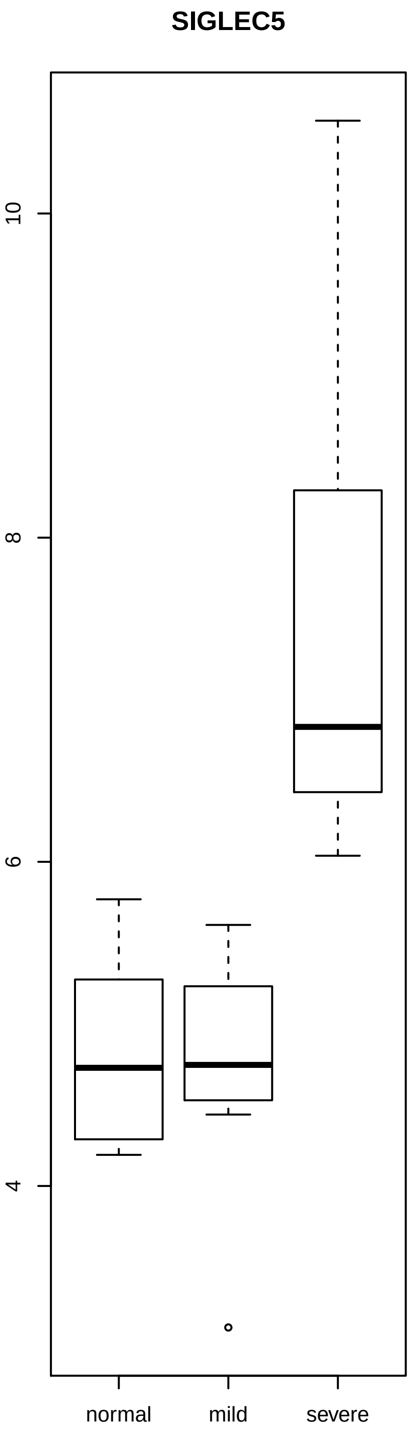
SIGLEC5
10
8
6
4
normal
mild
severe

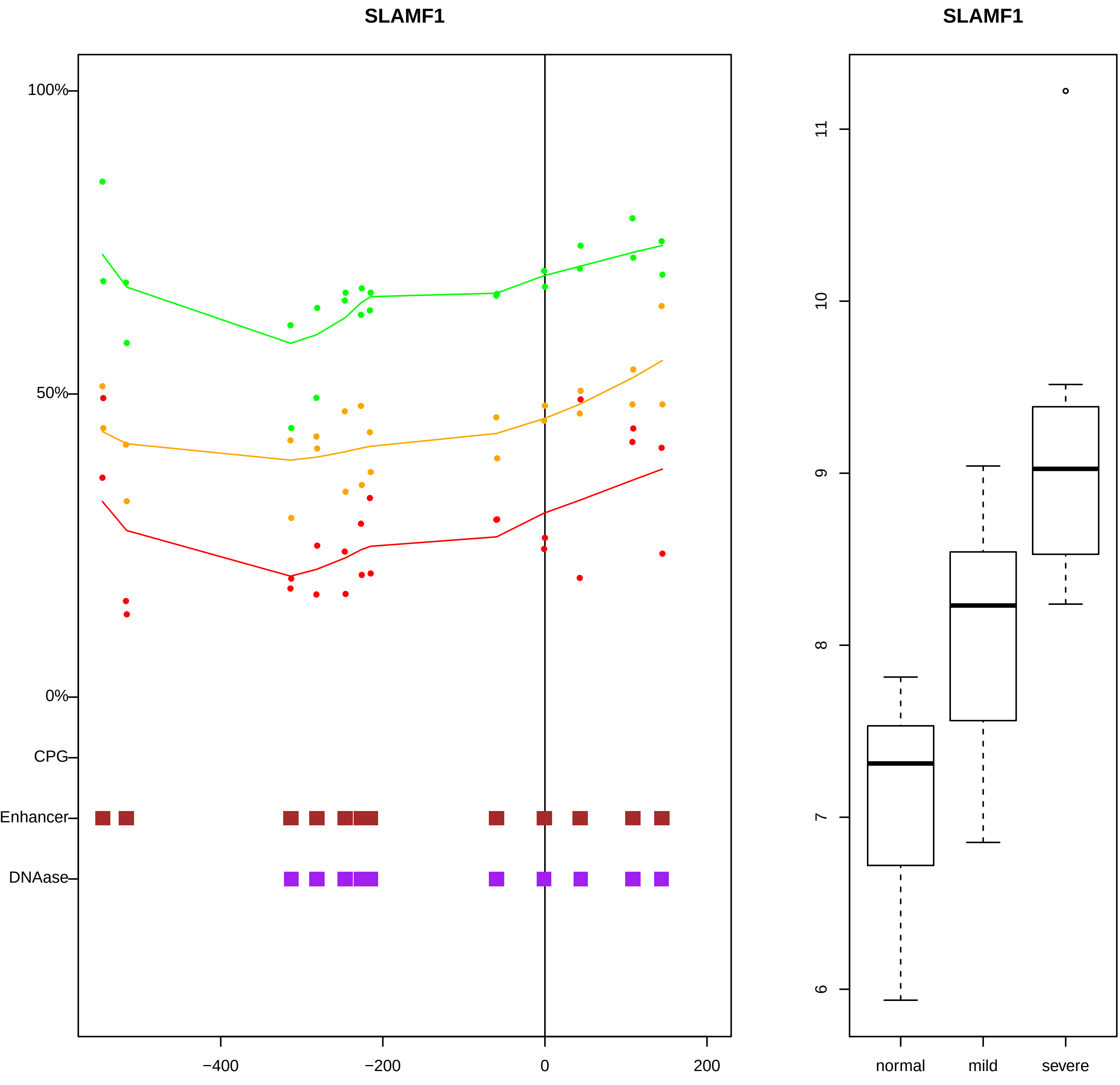
SLAMF1
100%
50%
0%
CPG
Enhancer
DNAase
−400
−200
0
200
SLAMF1
11
10
9
8
7
6
normal
mild
severe

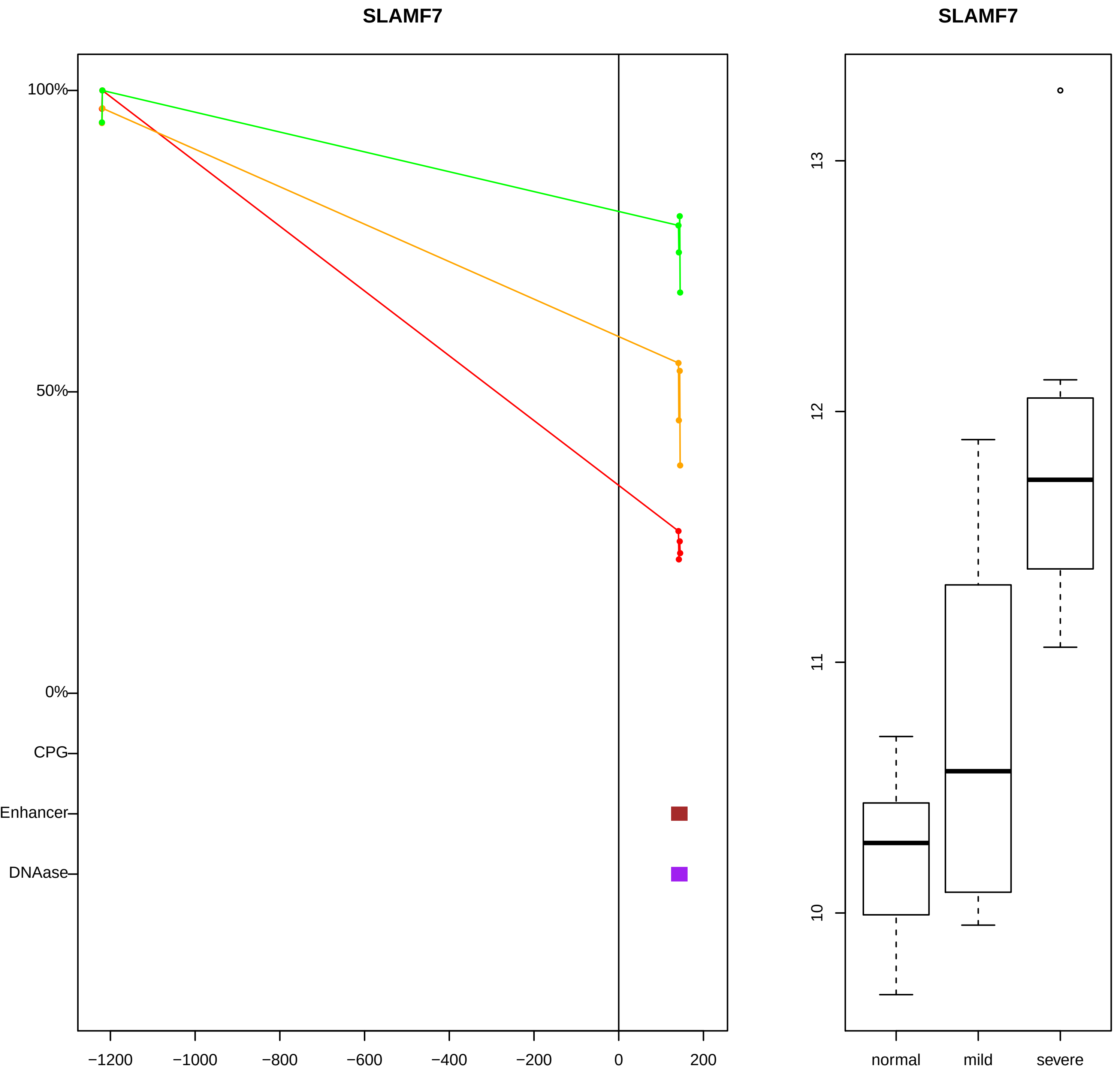
SLAMF7
100%
50%
0%
CPG
Enhancer
DNAase
−1200
−1000
−800
−600
−400
−200
0
200
SLAMF7
13
12
11
10
normal
mild
severe

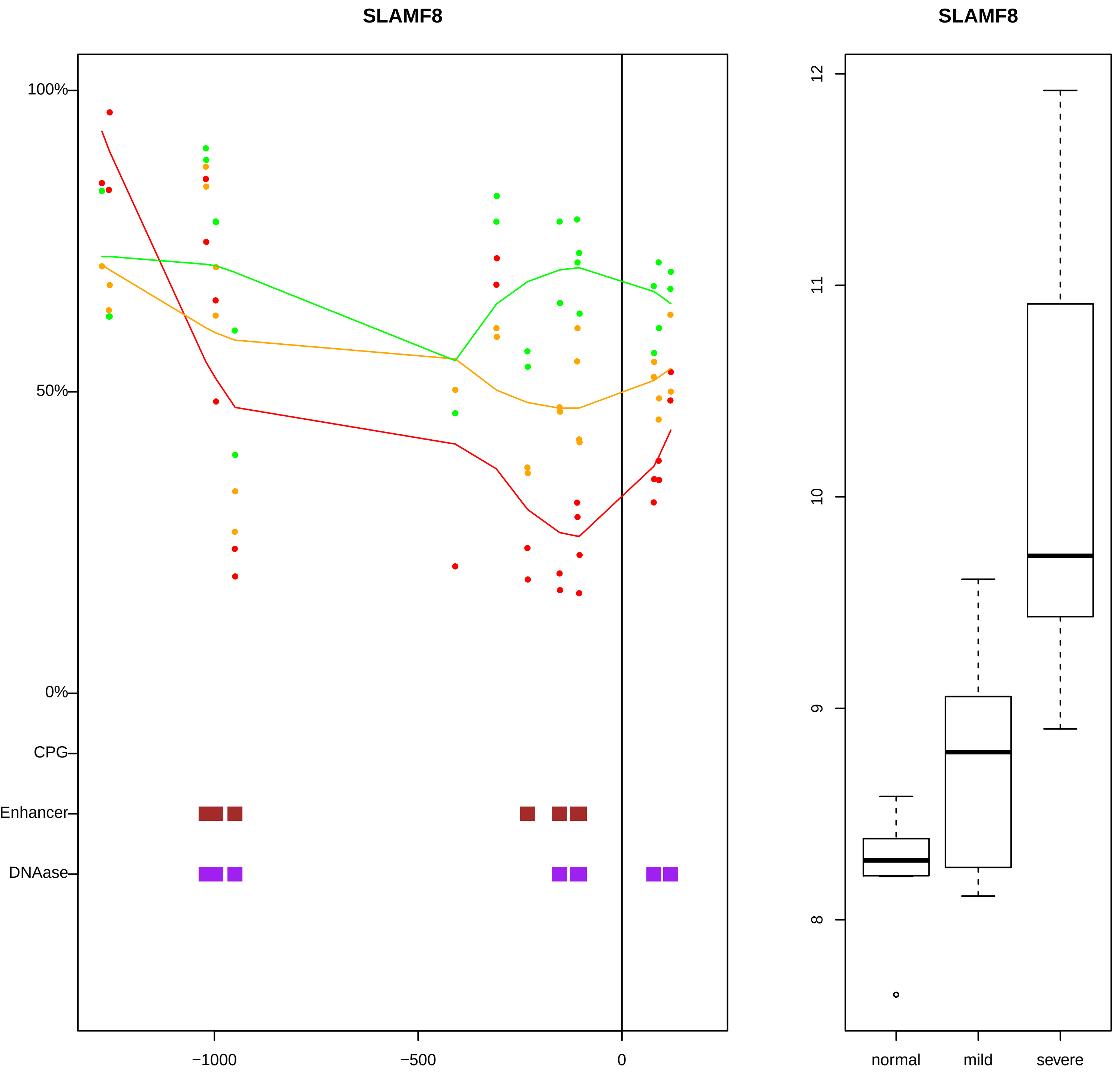
SLAMF8
100%
50%
0%
CPG
Enhancer
DNAase
−1000
−500
0
SLAMF8
12
11
10
9
8
normal
mild
severe

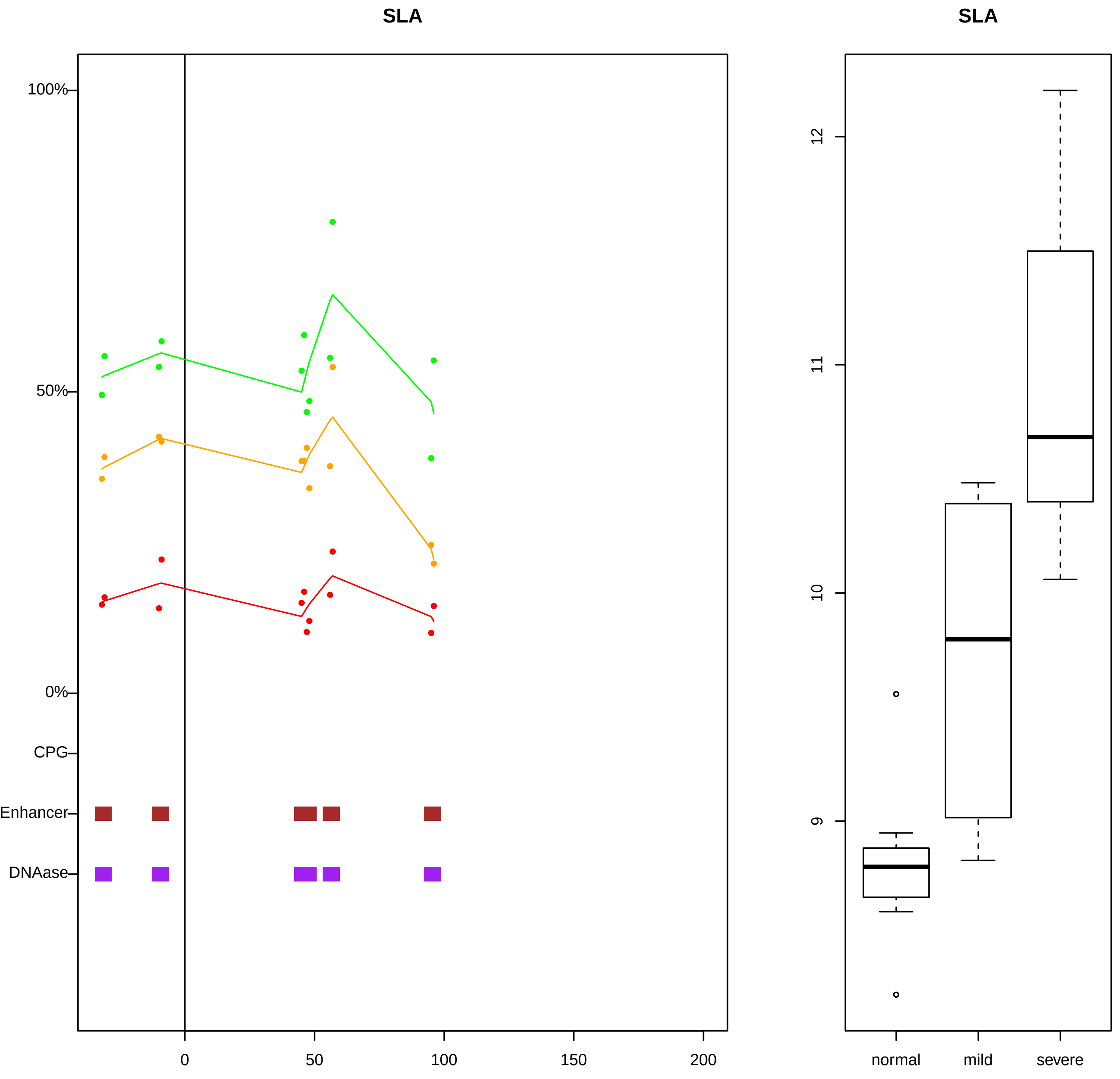

SLA
100%
50%
0%
CPG
Enhancer
DNAase
0
50
100
150
200
SLA
12
11
10
9
normal
mild
severe

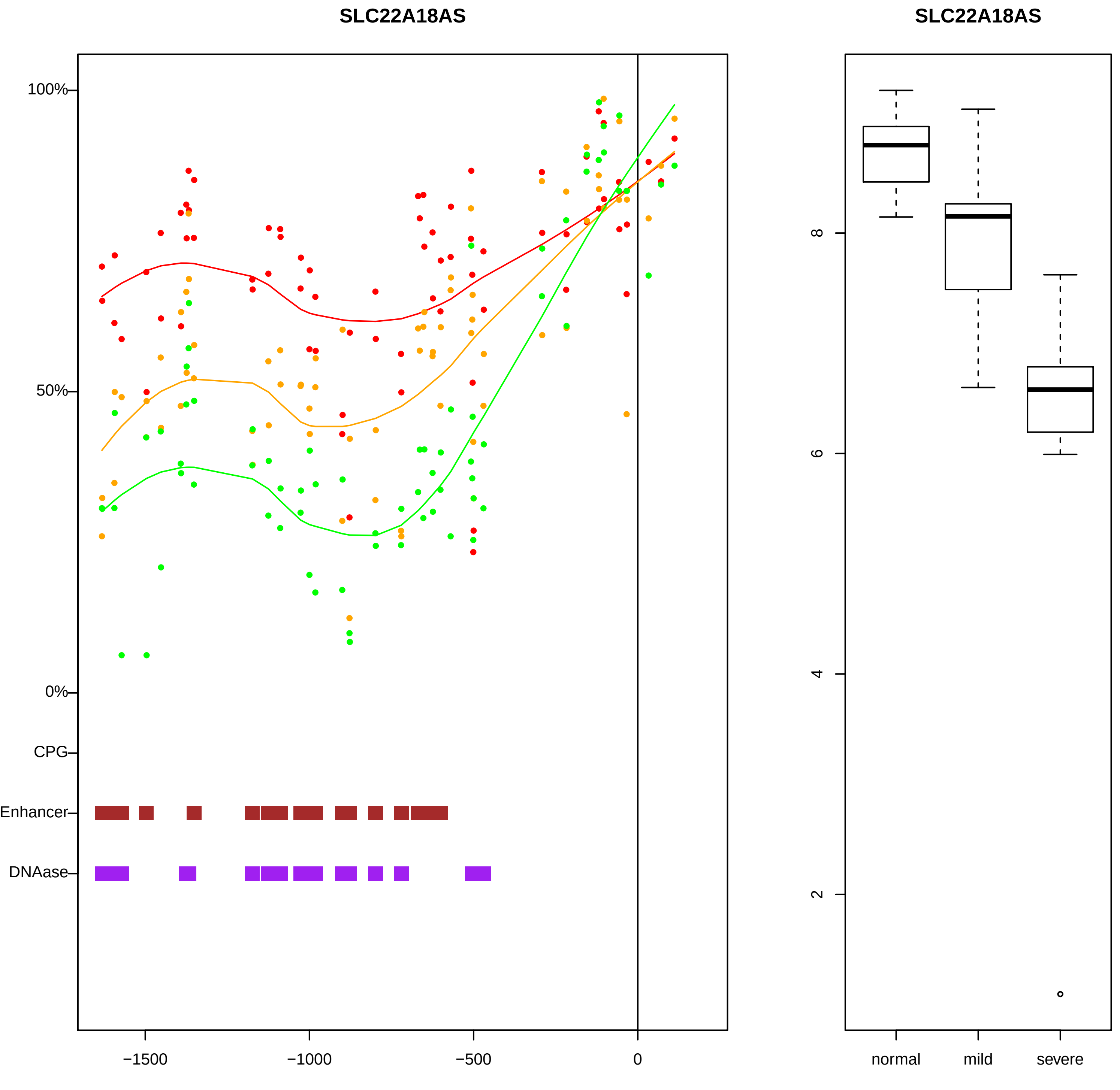
SLC22A18AS
100%
50%
0%
CPG
Enhancer
DNAase
−1500
−1000
−500
0
SLC22A18AS
8
6
4
2
normal
mild
severe

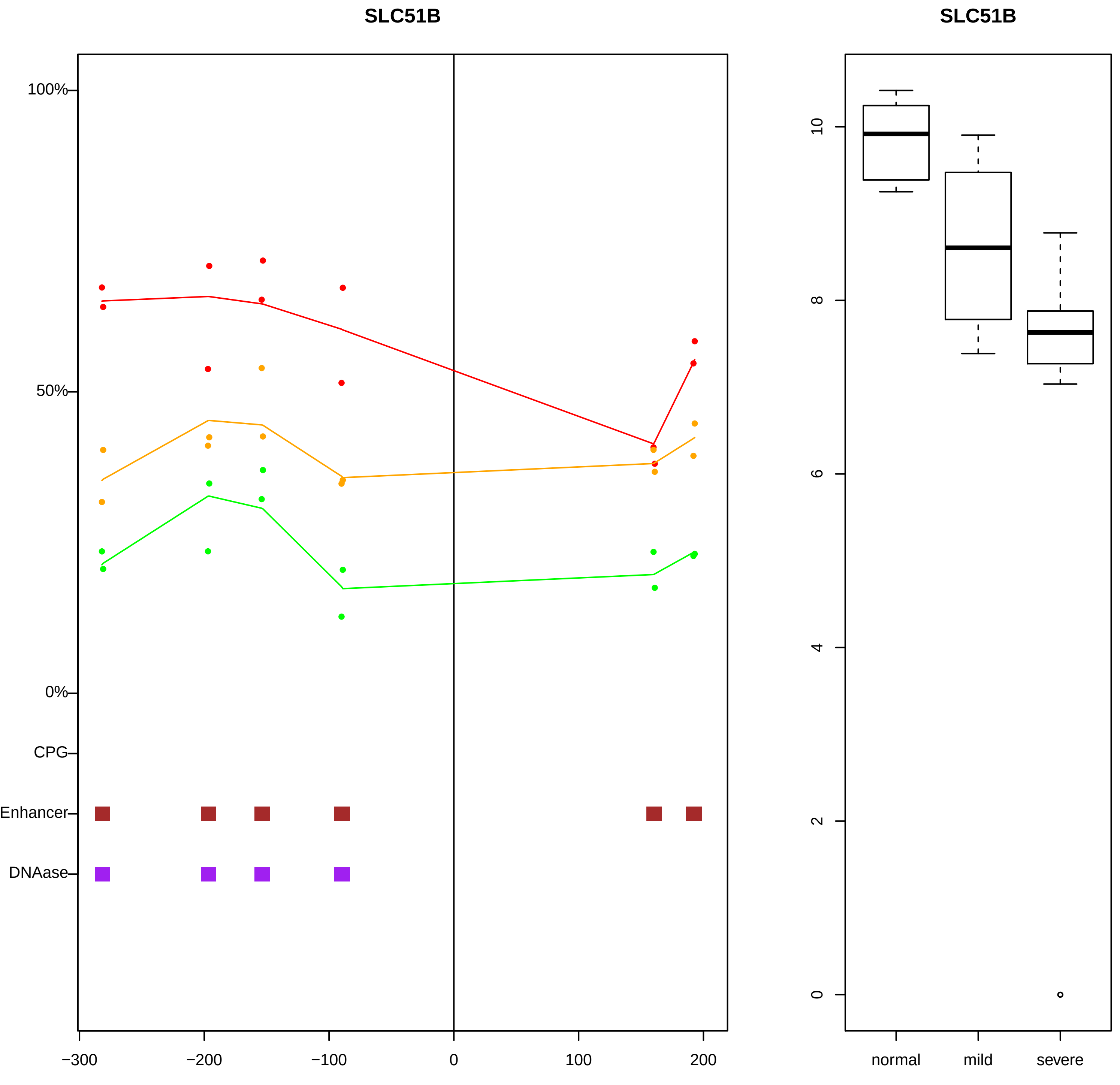
SLC51B
100%
50%
0%
CPG
Enhancer
DNAase
−300
−200
−100
0
100
200
SLC51B
0
2
4
6
8
10
normal
mild
severe

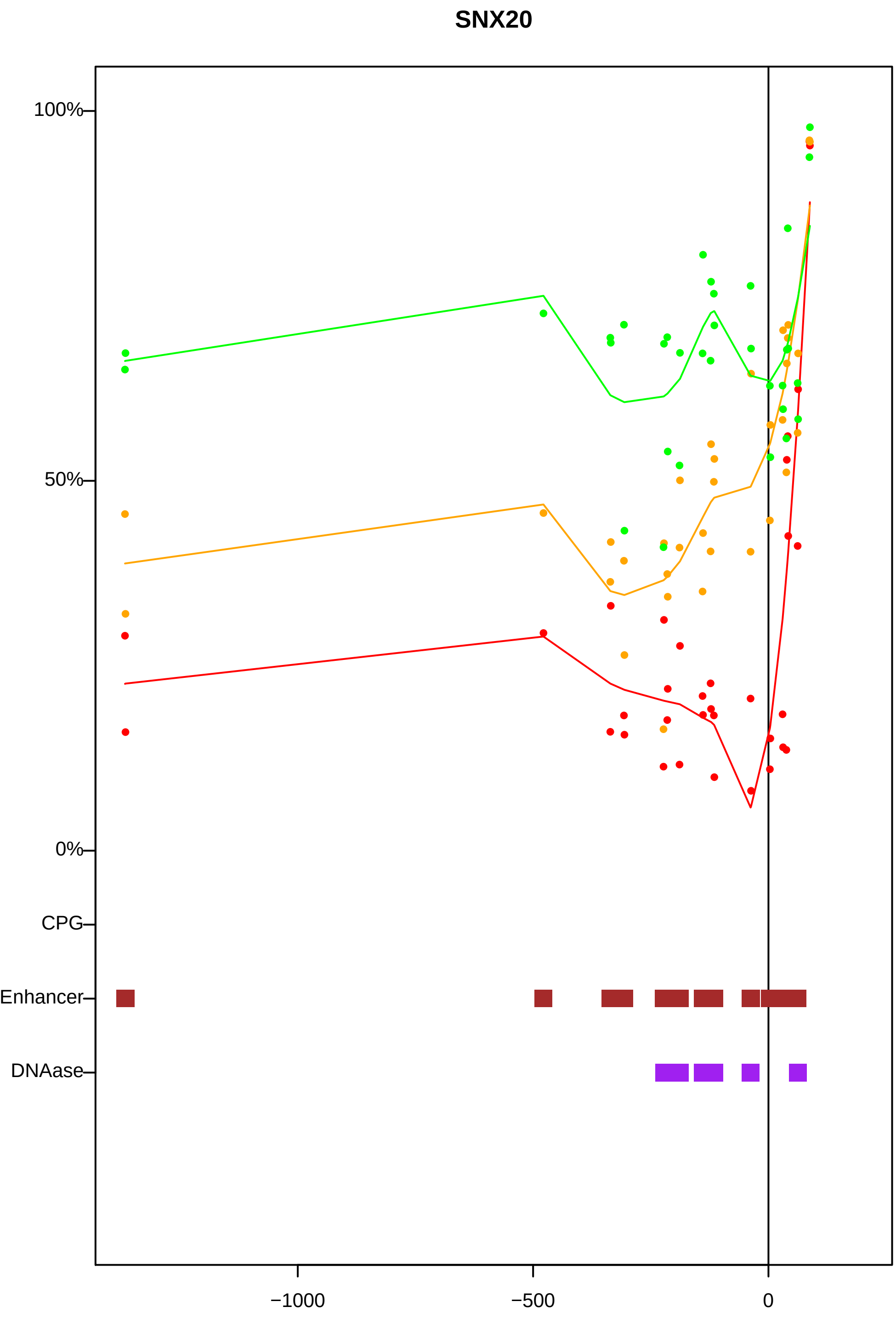
SNX20
100%
50%
0%
CPG
Enhancer
DNAase
−1000
−500
0

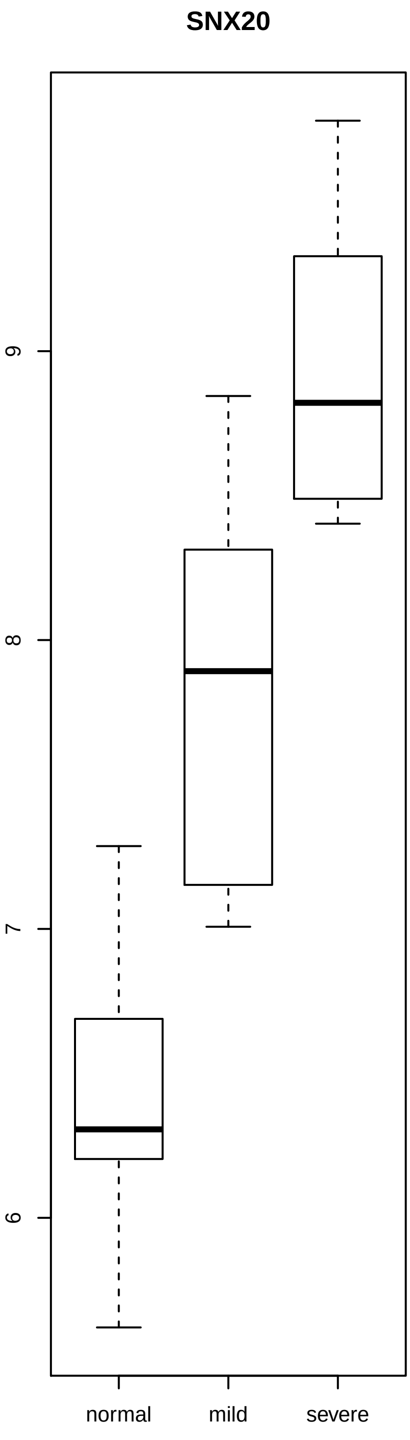
SNX20
9
8
7
6
normal
mild
severe

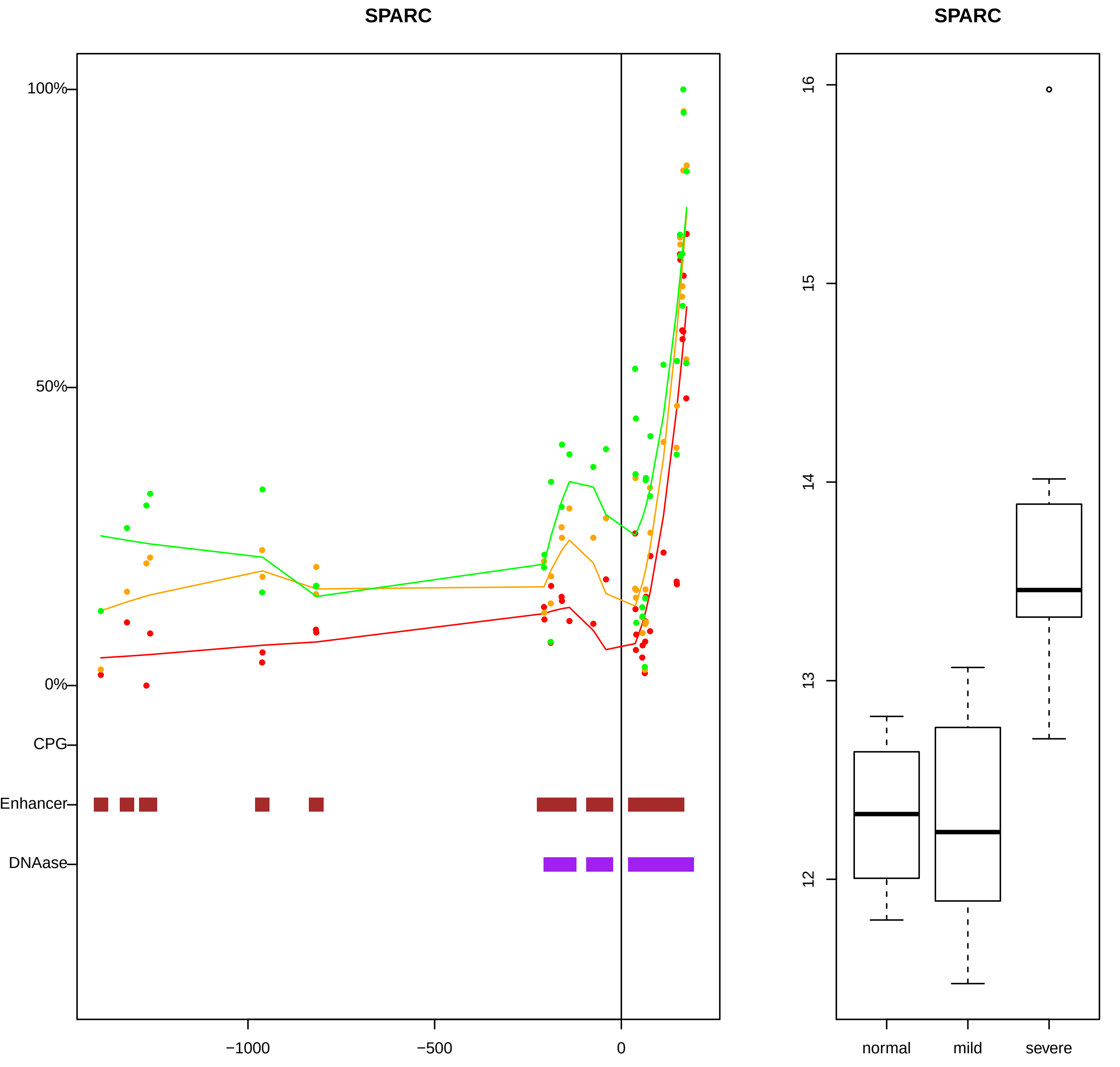
SPARC
100%
50%
0%
CPG
Enhancer
DNAase
−1000
−500
0
SPARC
16
15
14
13
12
normal
mild
severe

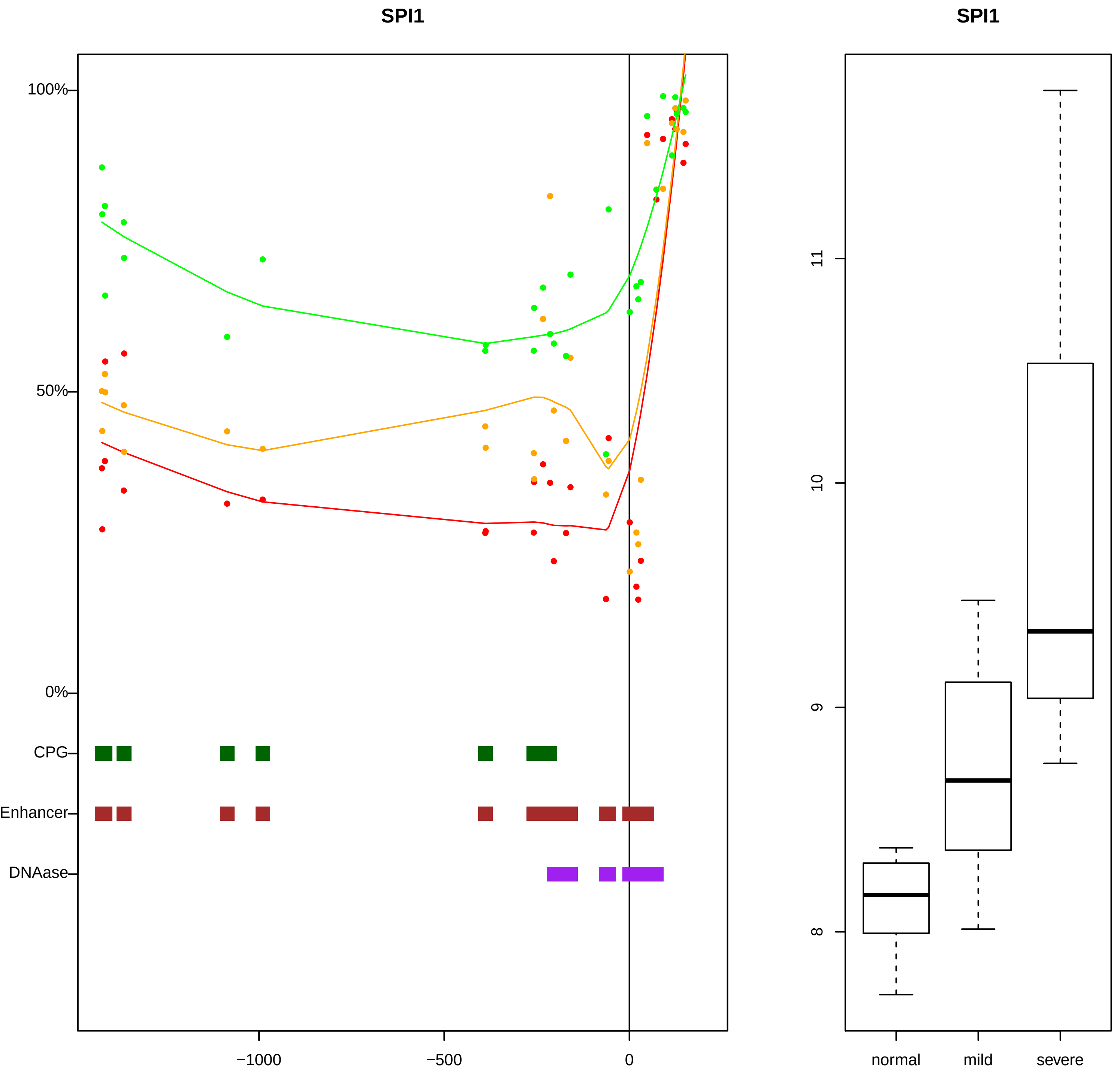

SPI1
100%
50%
0%
CPG
Enhancer
DNAase
−1000
−500
0
SPI1
11
10
9
8
normal
mild
severe

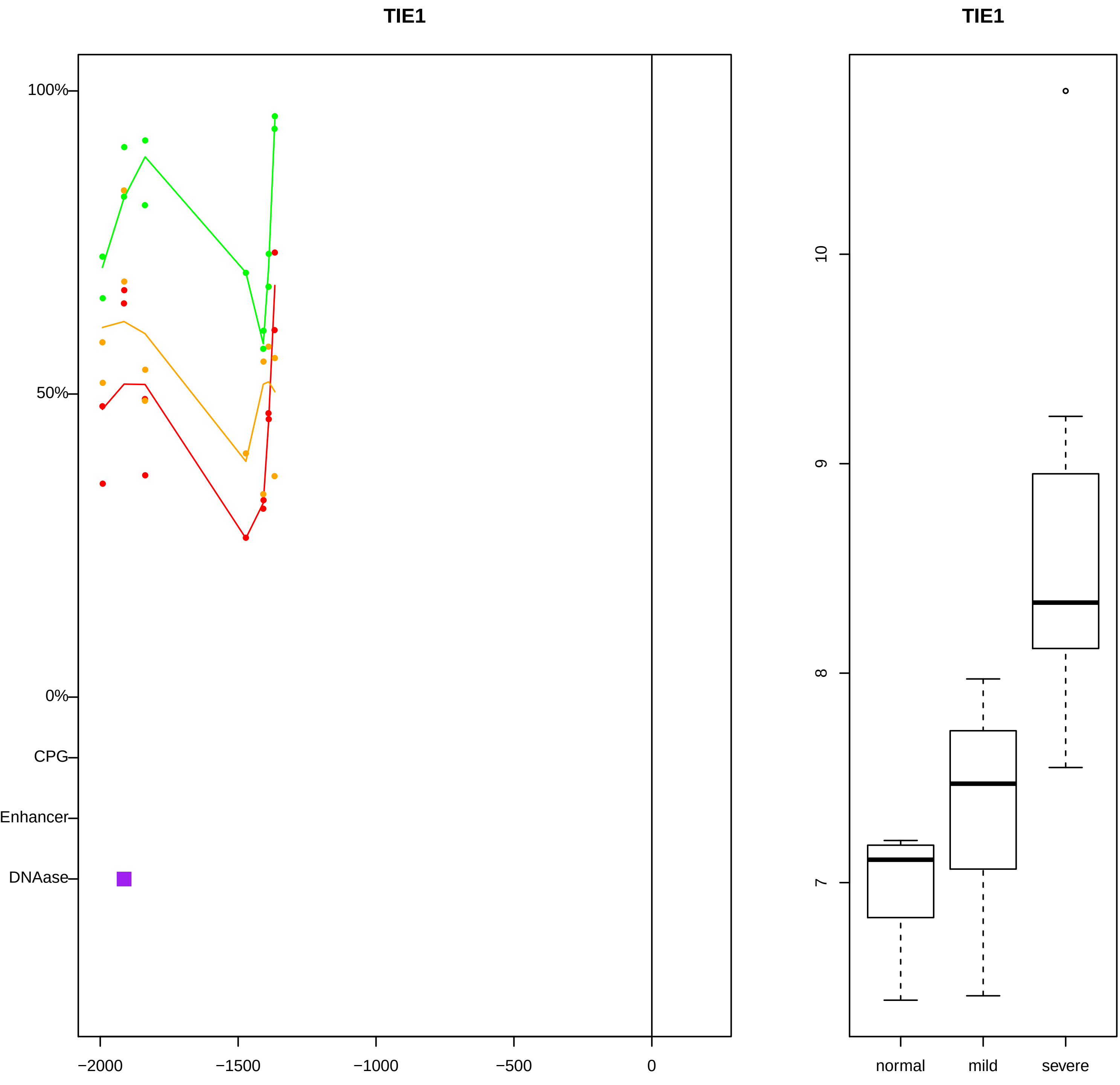

TIE1
100%
50%
0%
CPG
Enhancer
DNAase
−2000
−1500
−1000
−500
0
TIE1
10
9
8
7
normal
mild
severe

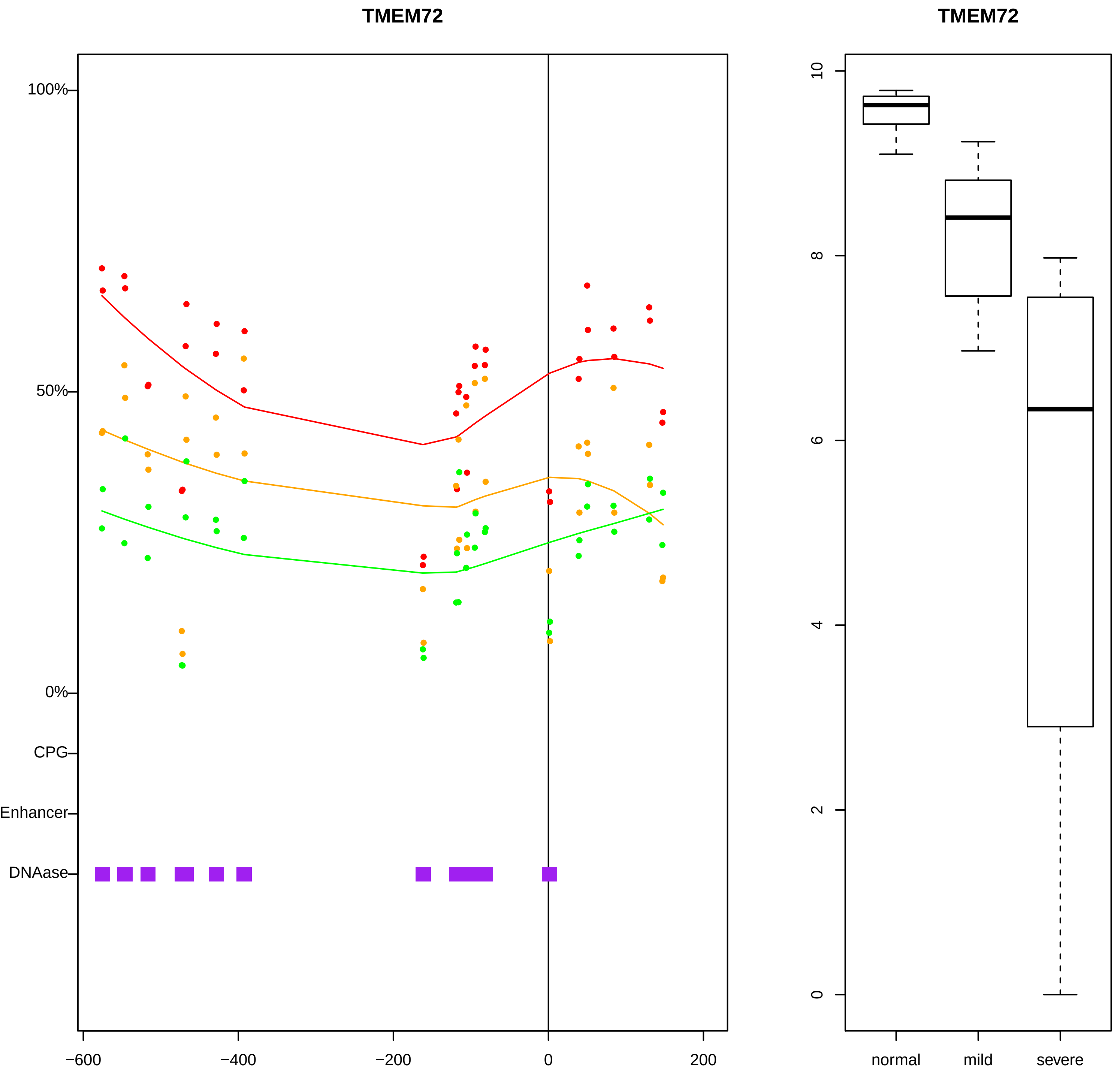
TMEM72
100%
50%
0%
CPG
Enhancer
DNAase
−600
−400
−200
0
200
TMEM72
10
8
6
4
2
0
normal
mild
severe

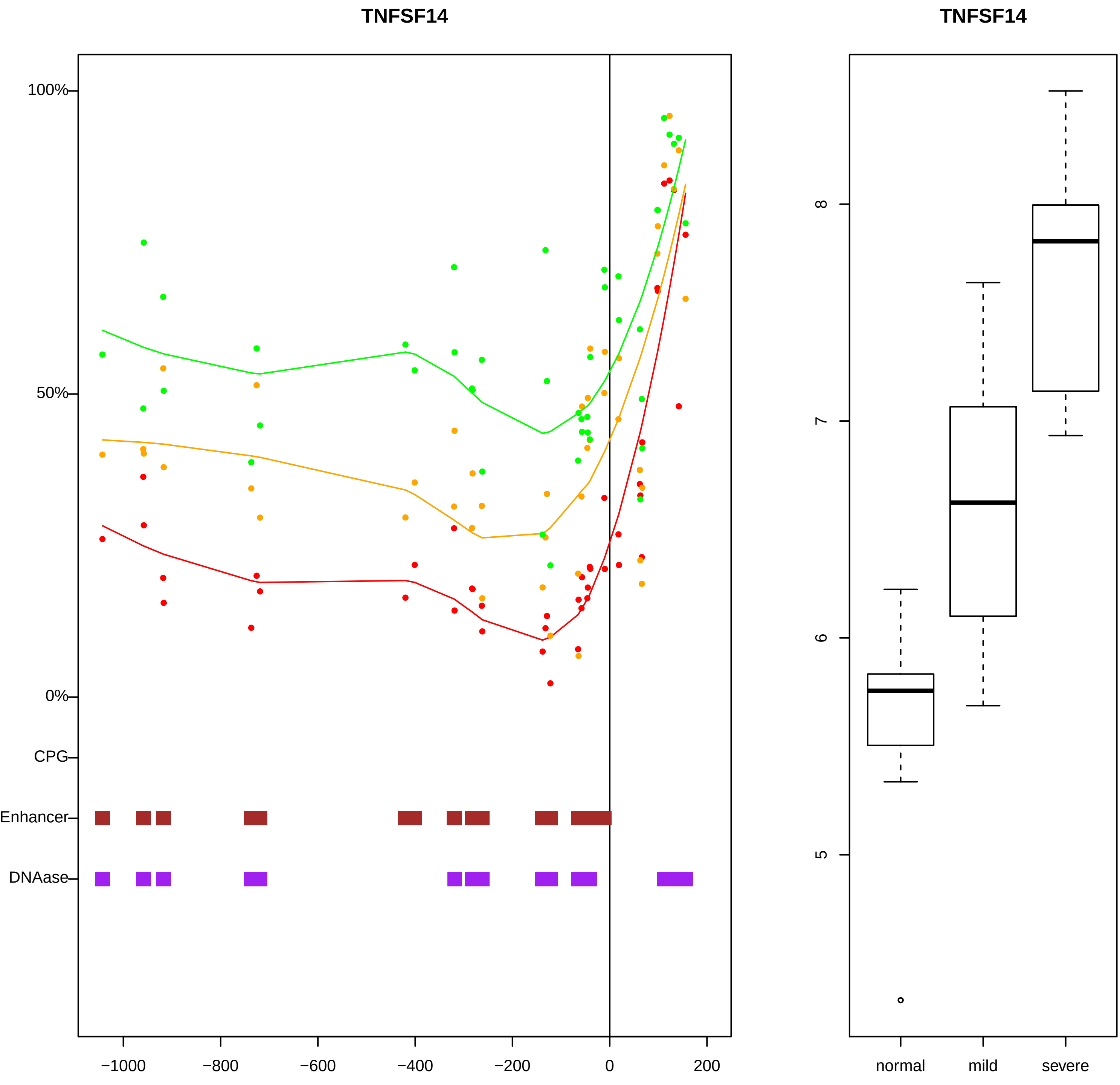
TNFSF14
100%
50%
0%
CPG
Enhancer
DNAase
−1000
−800
−600
−400
−200
0
200
TNFSF14
5
6
7
8
normal
mild
severe

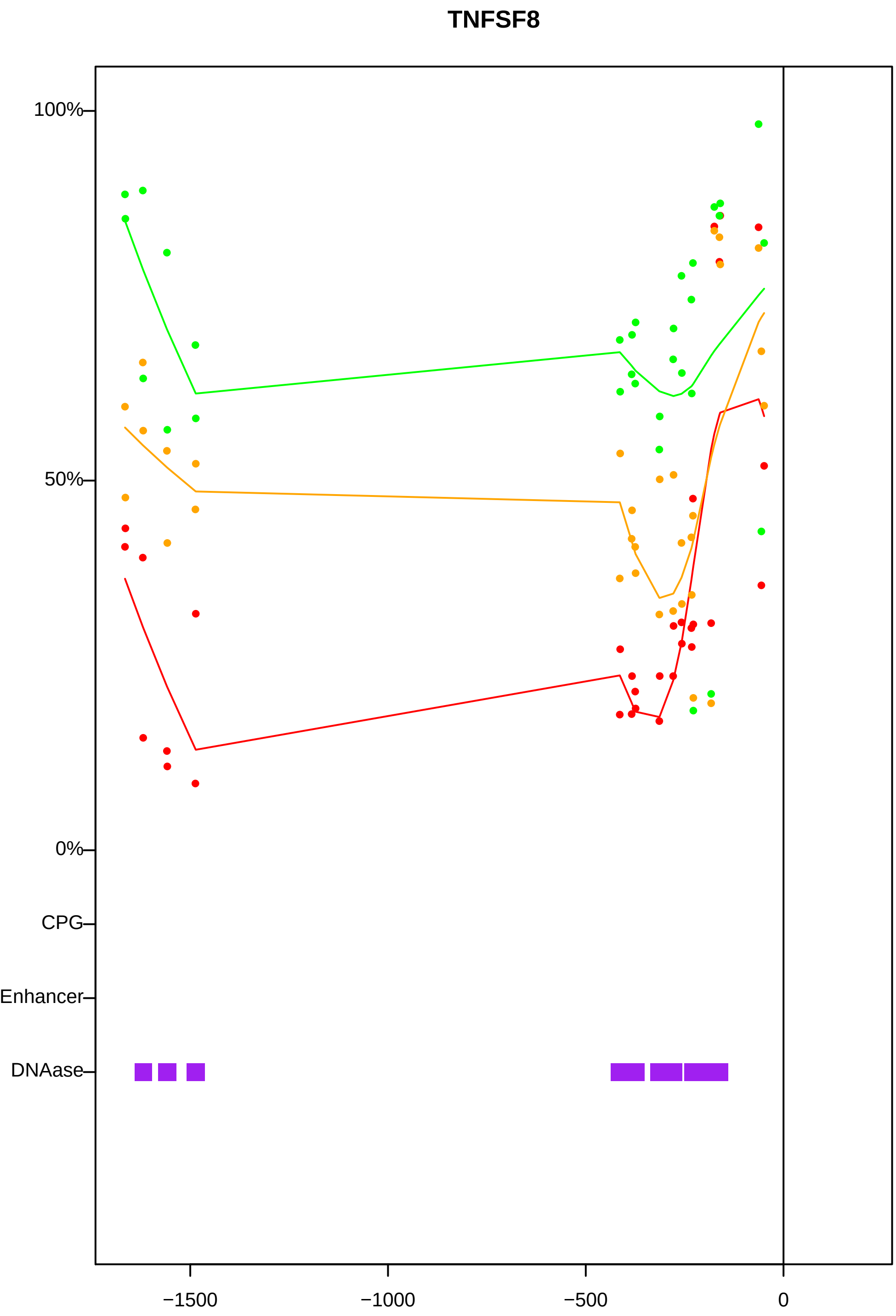
TNFSF8
100%
50%
0%
CPG
Enhancer
DNAase
−1500
−1000
−500
0

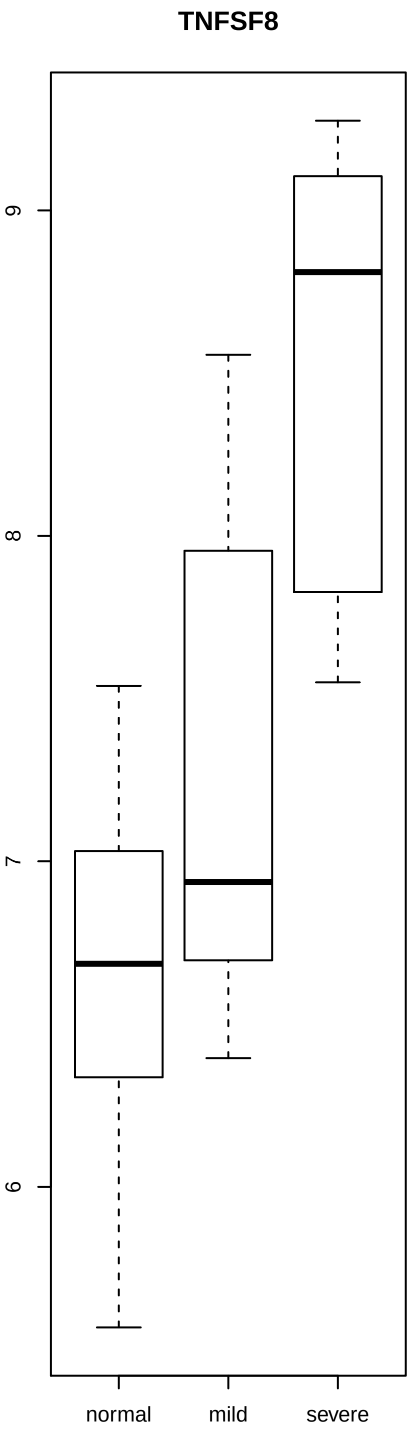
TNFSF8
9
8
7
6
normal
mild
severe

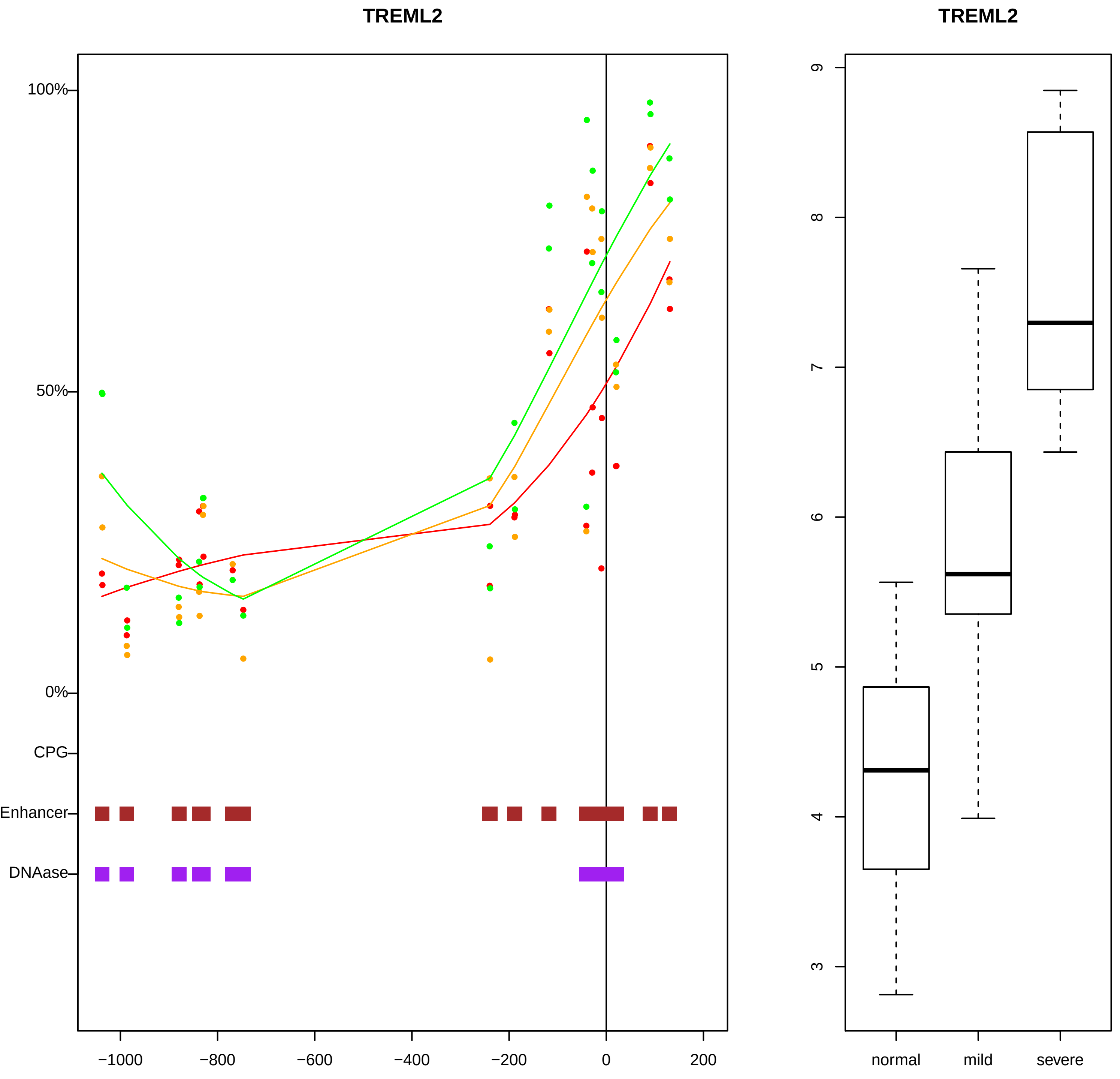
TREML2
100%
50%
0%
CPG
Enhancer
DNAase
−1000
−800
−600
−400
−200
0
200
TREML2
9
8
7
6
5
4
3
normal
mild
severe

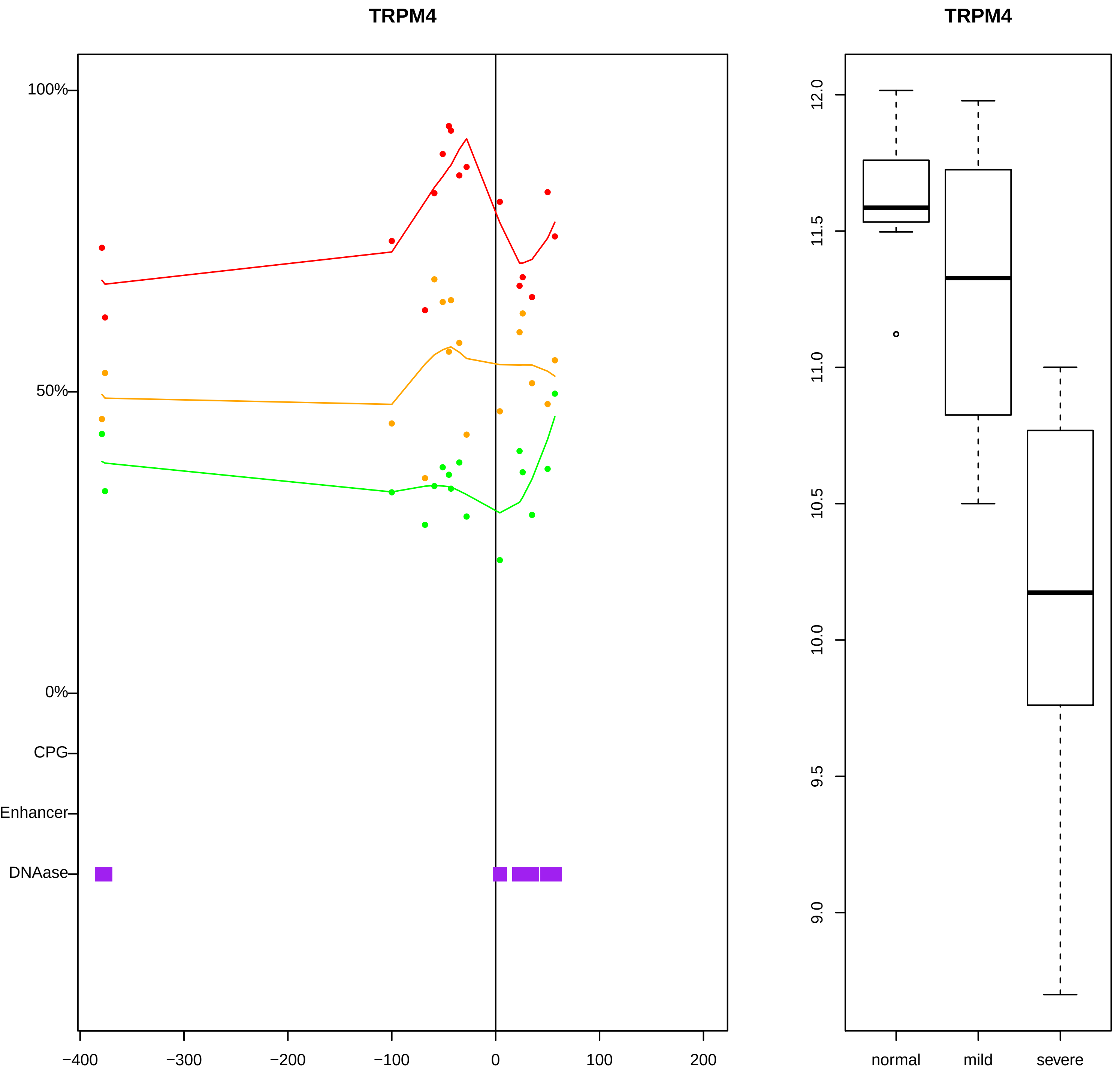
TRPM4
100%
50%
0%
CPG
Enhancer
DNAase
−400
−300
−200
−100
0
100
200
TRPM4
12.0
11.5
11.0
10.5
10.0
9.5
9.0
normal
mild
severe

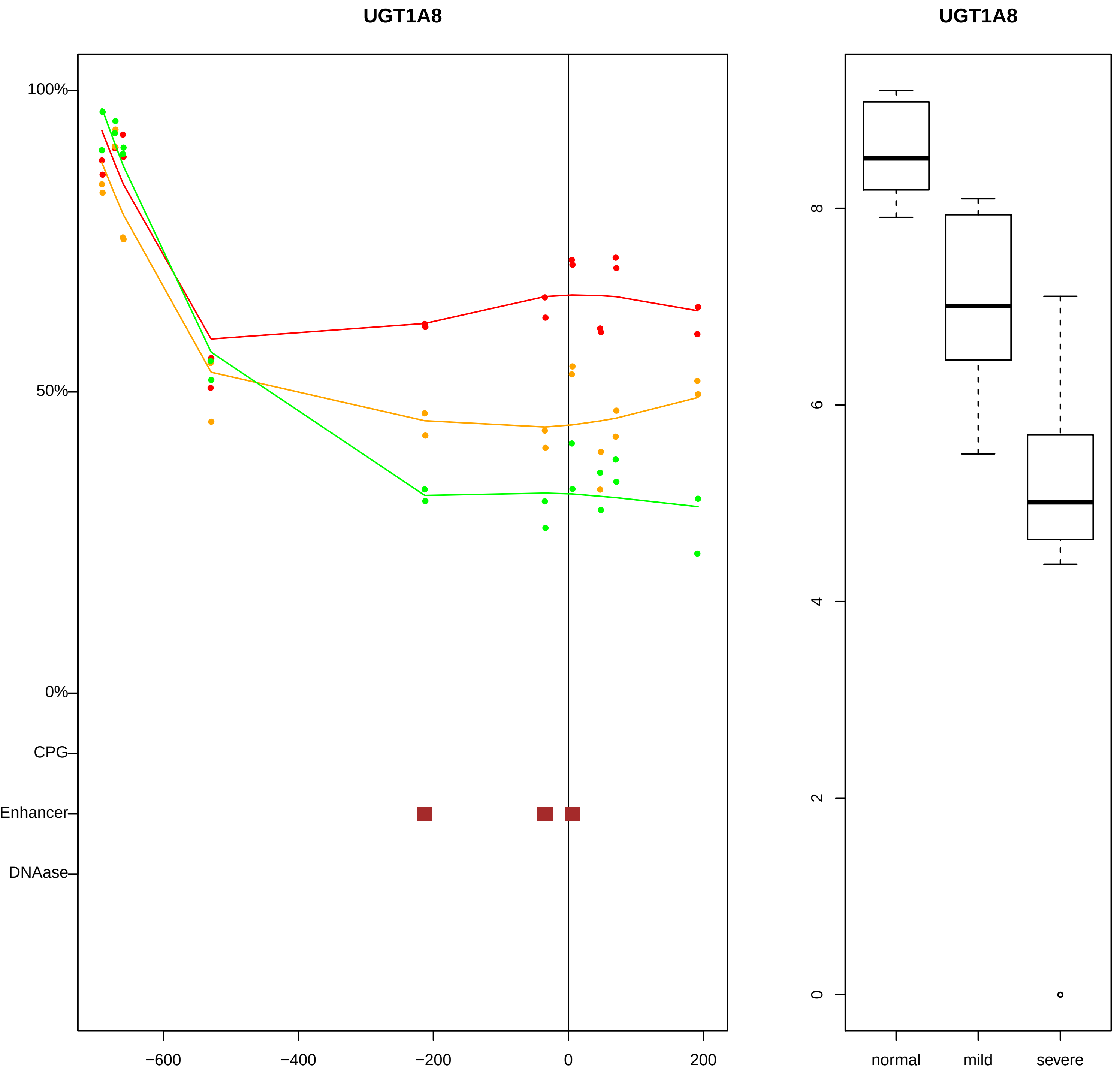

UGT1A8
100%
50%
0%
CPG
Enhancer
DNAase
−600
−400
−200
0
200
UGT1A8
0
2
4
6
8
normal
mild
severe
