# Supplementary material for: DNA hypo-methylation facilitates anti-inflammatory responses in severe ulcerative colitis
Source: PLoS One. 2021 Apr 1;16(4):e0248905. doi: 10.1371/journal.pone.0248905 (PMC8016308; doi:10.1371/journal.pone.0248905)
Supplement: S2 Table — (DOCX) [file pone.0248905.s003.docx]

**S2 Table:** **Differentially expressed IBD susceptibility genes in severe UC**

| **Gene symbol** | **log2 FC ≥ 1.0**  **p_adj._ < 0.05** |
| --- | --- |
| SELE | 3,59 |
| TNC | 3,36 |
| CXCR1 | 3,27 |
| FCGR3B | 3,12 |
| IL24 | 3,11 |
| SLC11A1 | 3,04 |
| HSPA6 | 2,79 |
| FCGR2A | 2,75 |
| CCR1 | 2,43 |
| CXCR2 | 2,42 |
| CCL2 | 2,40 |
| CXCL6 | 2,26 |
| FCGR3A | 1,93 |
| LRRK2 | 1,91 |
| SELL | 1,90 |
| LTF | 1,88 |
| ICAM1 | 1,79 |
| IL10 | 1,77 |
| OSMR | 1,74 |
| HCK | 1,74 |
| FOSL1 | 1,65 |
| NOD2 | 1,50 |
| FADS1 | 1,49 |
| FCGR2B | 1,48 |
| SELP | 1,42 |
| PLTP | 1,39 |
| CCR2 | 1,34 |
| DOK3 | 1,30 |
| SLAMF1 | 1,28 |
| NFIL3 | 1,27 |
| CD40 | 1,22 |
| IL18R1 | 1,19 |
| IL1R1 | 1,19 |
| TNFSF8 | 1,16 |
| CEBPB | 1,15 |
| TNFSF15 | 1,14 |
| IL12RB2 | 1,12 |
| GPR65 | 1,12 |
| CCR5 | 1,11 |
| SLAMF7 | 1,09 |
| PTPRC | 1,08 |
| CD48 | 1,06 |
| IRF4 | 1,04 |
| TNFRSF4 | 1,02 |
| MMP9 | 1,00 |
| GYPC | 1,00 |
| TST | -1,07 |
